# Supplementary figures and images for: Compliance with smoke-free laws in hospitality venues in Ethiopia: A cross-sectional observational study in 10 cities
Source: PLoS One. 2025 Feb 21;20(2):e0319079. doi: 10.1371/journal.pone.0319079 (PMC11844872; doi:10.1371/journal.pone.0319079)

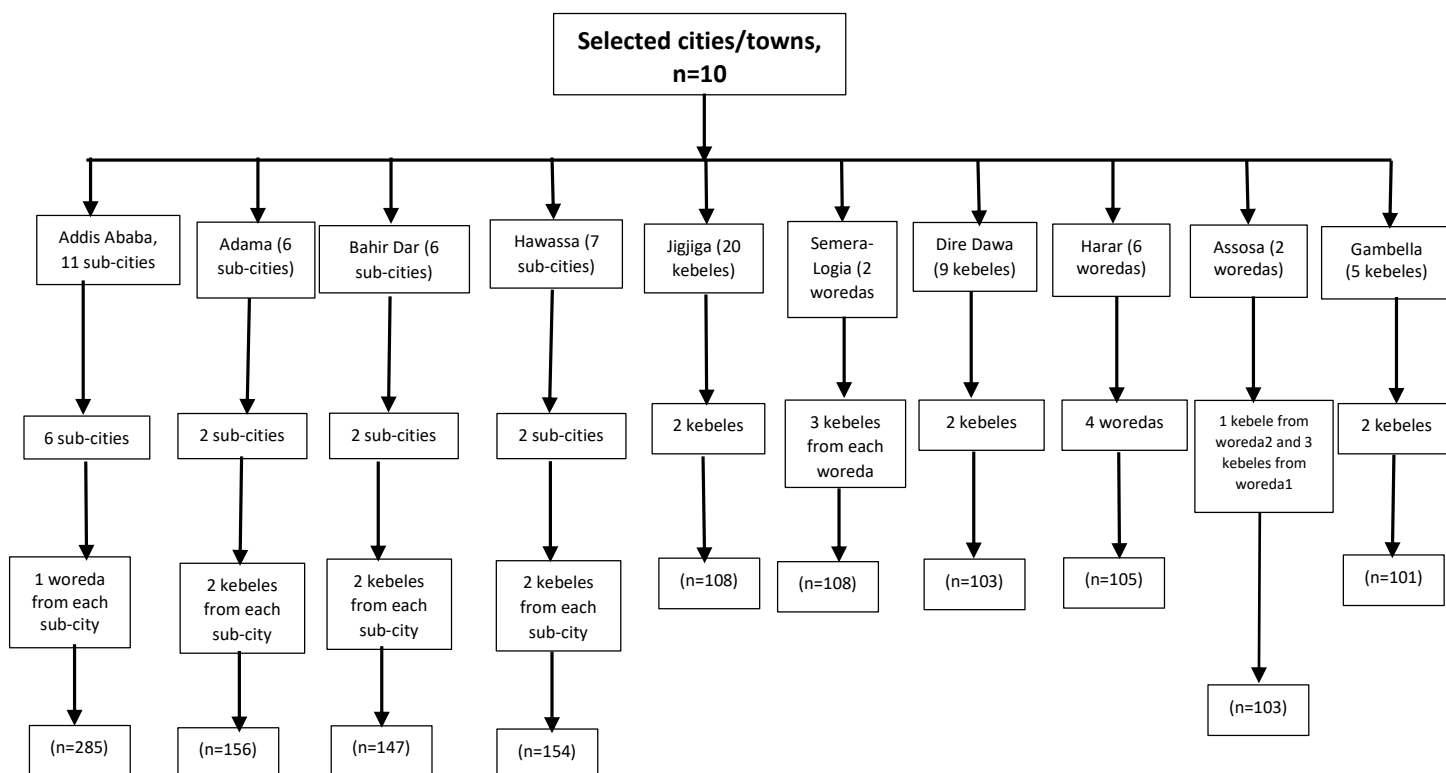

**S1 Fig. Multistage schematic sampling technique, Ethiopia, December 2022**

Supplement: S1 Fig — (PDF) [file pone.0319079.s001.pdf]
