# Supplementary material for: Compliance with smoke-free laws in hospitality venues in Ethiopia: A cross-sectional observational study in 10 cities
Source: PLoS One. 2025 Feb 21;20(2):e0319079. doi: 10.1371/journal.pone.0319079 (PMC11844872; doi:10.1371/journal.pone.0319079)
Supplement: S1 File — (PDF) [file pone.0319079.s002.pdf]

S1 File. Smoke-Free Study Tool

SECTION I: Hospitality Venue Identifier

Variable Name	QUESTIONS	Response/response categories	Skip
HVID	Unique ID for the hospitality venue (HV)	☐☐☐☐	
HV101	Administrative Region	1. Addis Ababa<br>2. Oromia<br>3. Amhara<br>4. Sidama<br>5. Somali<br>6. Afar<br>7. Dire Dawa<br>8. Harari<br>9. Benishangul Gumuz<br>10. Gambela	
HV102	City/town	1. Addis Ababa<br>2. Adama<br>3. Bahir Dar<br>4. Hawassa<br>5. Jigjiga<br>6. Semera-Logia<br>7. Dire Dawa<br>8. Harar<br>9. Assosa<br>10. Gambela	
HV103	Zone/sub-city		
HV104	Woreda		
HV105	Kebele		
HV106	Date of the visit (DD/MM/YY)	└─┴─┘ └─┴─┘ └─┴─┘<br>**dd** **mm** **year**	
HV107	Types of hospitality venue	1. Restaurant<br>2. Café and restaurant<br>3. Bar and restaurant<br>4. Hotel<br>5. Grocery<br>6. Butcher house and restaurant<br>7. Bar<br>8. Nightclub/Lounge	
HV108	Does this venue sell alcoholic drinks?	0.No<br>1. Yes	
HV109	Does this venue sell food?	0.No<br>1. Yes	

HV110	Nature of the venue	1. Both indoor and outdoor facilities<br>2. Only indoor facilities<br>3. Only outdoor facilities	If the answer is "2"the outdoor facilities questions will be skipped.<br>If the answer is "3" skip to HV216

SECTIONS II: Compliance with Smoke-free Law in Ethiopia

Instructions: Use the Indoor observation checklist if the venue has indoor space. Use the outdoor observation checklist if the venue has outdoor space. Use both if the venue has both indoor and outdoor spaces.

Variable Name	QUESTIONS	Response/response categories	Skips
	**Indoor observation**		
HV201	Does this establishment offer tobacco products for sale in the indoor space?	0.No<br>1. Yes	If "No" go to question HV203
HV202	If yes to question HV201, what kinds of tobacco products are available for sale? Select all that apply	1. Cigarettes<br>2.Cigar<br>3.Shisha<br>4.Electronic cigarettes<br>5.Smokeless tobacco products<br>96. Others, specify	
HV203	Is anyone use tobacco products in the indoor place?	0.No<br>1. Yes	If "No" go to question HV207
HV204	If yes to question HV203, in which part of the house is observed?<br>Select all that apply	1. Main room<br>2. Corridor<br>3. Toilet<br>4. Porch<br>5. Others, please specify	
HV205	If yes to question HV203, what are the tobacco products?<br>Select all that apply	1. Cigarettes<br>2.Cigars<br>3.Shisha<br>4.Electronic cigarette<br>5.Smokeless tobacco products<br>96. Others, specify	

HV206	If no to question number HV203, do you smell cigarette smoke in indoor space?	0.No<br>1. Yes	
HV207	Do you observe any ashtrays visible indoors?	0.No<br>1. Yes	
HV208	Do you observe any lighter?	0.No<br>1.Yes	
HV209	Do you observe any shisha equipment?	0.No<br>1.Yes	
HV210	If no to question number HV2083, do you smell shisha smoke in indoor space?		
HV211	Did you observe cigarette butts visible indoors?	0.No<br>1. Yes<br>2. I can't observe	
HV212	Did you see a designated indoor smoking area? (Choose "YES" if you see a designated area, even if no one is smoking in it.)	0.No<br>1. Yes	
HV213	Does the venue post in a visible manner a "SMOKING IS PROHIBITED" or "NO SMOKING" sign in a relevant spot in indoor places, or premises?	0.No<br>1. Yes	
HV214	Is anyone use tobacco products in the outdoor place within 10 meters from any door, window, or air intake mechanism?	0.No<br>1. Yes	
HV215	Record any other comments regarding your indoor observation.		
	**Outdoor observation**		
HV216	Does this establishment offer tobacco products for sale in the outdoor space?	0. No<br>1. Yes	If "No" go to question number HV218
HV217	If yes to question HV214, what kinds of tobacco products are available for sale?		
a.	Cigarettes	0. No<br>1. Yes	
b.	Cigars	0. No<br>1. Yes	
c.	Shisha	0. No<br>1. Yes	
d.	Electronic nicotine delivery systems	0. No	
e.	Smokeless tobacco products	1. Yes	
X	Other, specify	________________	
HV218	Is anyone use tobacco products in outdoor place?	0. No<br>1. Yes	If "No" go to question number HV220
HV219	If yes to question HV216, what are the tobacco products?		

a.	Cigarette	0.No<br>1. Yes	
b.	Cigar	0.No<br>1. Yes	
c.	Shisha	0.No<br>1.Yes	
D	Electronic nicotine delivery systems	0.No<br>1. Yes	
e.	Smokeless tobacco products	0.No<br>1.Yes	
96.	Others, specify		
HV220	If no to question number HV218, do you smell cigarette smoke in the outdoor space?	0.No<br>1.Yes	
HV221	Does the venue have an outdoor designated smoking area (Choose “YES” if you see a designated area, even if no one is smoking in it.)	0.No<br>1. Yes	
HV222	Are there any ashtrays visible in outdoor space?	0.No<br>1.Yes	
HV223	Did you observe cigarette butts outdoors?	0.No<br>1. Yes	
HV224	Does the venue post in a visible manner a “SMOKING IS PROHIBITED” or “NO SMOKING” signage in a relevant spot in outdoor places, or premises?	0. No<br>1. Yes	
HV225	Record any other comments regarding your outdoor observation		
