# Supplementary material for: Compliance with smoke-free laws in hospitality venues in Ethiopia: A cross-sectional observational study in 10 cities
Source: PLoS One. 2025 Feb 21;20(2):e0319079. doi: 10.1371/journal.pone.0319079 (PMC11844872; doi:10.1371/journal.pone.0319079)
Supplement: S2 File — (PDF) [file pone.0319079.s003.pdf]

S2 File. Operational definitions

Active smoking: means being in possession or control of a lit tobacco product, including cigarettes, cigars, and shisha, inside or outside of HVs at the time of data collection.

Adherence: encompasses how HVs comply with various provisions, such as displaying 'no smoking' signs, removing ashtrays and lighters, banning DSAs, absence of cigarette butts, prohibiting the sale of tobacco products, and forbidding tobacco use within a 10-meter radius of doorways, windows, or air intake mechanisms of public workplaces.

Bar: an establishment where alcoholic drinks and sometimes food is served to clients.

Bar and restaurant: an establishment that combines both a bar and a restaurant where customers can find both a bar area, where alcoholic beverages are served, and a restaurant section, where meals are prepared and served.

Butcher shop and restaurant: an establishment that combines a butcher shop and restaurant where customers can buy fresh meat, and can enjoy meals and drinks in an integrated restaurant.

Café: an establishment where simple meals and drinks (such as tea, coffee, and milk) are served to clients.

Café and restaurant: an establishment that combines both café and restaurant where customers can enjoy drinks (such as tea, coffee, and milk) and more extended meals.

Compliance: refers to the degree to which the HVs fully implementing tobacco control laws under the 2019 Ethiopian Tobacco Control Law (Proclamation No.1112/2019).

Designated smoking area (DSA): refers to the part of an HV that has been set or designated as a smoking area by the owner, manager, or other individual in charge of the HV.

Grocery: a small store that primarily retails a general range of alcoholic drinks such as liquor, wine, and beer.

Hospitality venue: refers to an establishment registered under the regulation of the Government of Ethiopia where food and beverages are sold and consumed, namely hotels, restaurants, bars, bars and restaurants, café and restaurant, butcher shop and restaurant, grocery, nightclub and lounges.

Hotel: an establishment offering lodging, food and beverage, and as may be needed, recreational, conference and similar facilities to clients.

Indoor space: any space that is covered by a roof or enclosed by one or more walls or sides, regardless of the material used for the structure. The definition encompasses outdoor extensions such as porches, verandahs, and similar areas, whether they are permanent or temporary.

Lounge: a place where customers enjoy alcoholic beverages while listening to soothing music or watching television. In this study, however, lounge serves as a nightclub since the later is regarded as illegal.

Nightclub: a place of entertainment open to clients at night usually serving food and liquor and providing music and space for dancing.

'No smoking' signage: refers to any written, graphical or pictorial warning posted in the local language in indoors or outdoors of HVs and alerts people that smoking is prohibited in the area.

Outdoor space: any space outside of any HV that is not enclosed, excluding any verandah, exterior wall or a window facing outward in any such venue.

Restaurant: an establishment offering food and beverage services to clients.

Tobacco product: means a product entirely or partly made of the tobacco leaf as a raw material which are manufactured to be used for smoking, chewing, sucking, or snuffing.

True compliance: refers to the law's effectiveness in preventing active smoking in prohibited areas and serves as a key indicator of its success.
