# Supplementary material for: Compliance with smoke-free laws in hospitality venues in Ethiopia: A cross-sectional observational study in 10 cities
Source: PLoS One. 2025 Feb 21;20(2):e0319079. doi: 10.1371/journal.pone.0319079 (PMC11844872; doi:10.1371/journal.pone.0319079)
Supplement: Attachment — Submitted filename: renamed_20a8f.pdf [file pone.0319079.s005.pdf]

PLOS ONE

Compliance with smoke-free laws in hospitality venues in Ethiopia: a cross-sectional observational study in 10 cities

--Manuscript Draft--

Manuscript Number:	PONE-D-24-30728
Article Type:	Research Article
Full Title:	Compliance with smoke-free laws in hospitality venues in Ethiopia: a cross-sectional observational study in 10 cities
Short Title:	Smoke-free Law Compliance in Ethiopia
Corresponding Author:	Wakgari Deressa, PhD<br>Addis Ababa University<br>Addis Ababa, ETHIOPIA
Keywords:	compliance; Hospitality venue; Smoke-free laws; Smoking; Ethiopia
Abstract:	Background<br>The 2019 Ethiopia's comprehensive tobacco control proclamation mandates 100% smoke-free public places and workplaces. Despite the proclamation, compliance remains uncertain, particularly at hospitality venues (HVs) such as restaurants, bars, nightclubs, and hotels. The aim of this study was to evaluate the extent of compliance with smoke-free laws in HVs and identify factors associated with non-compliance.<br><br>Methods<br>This cross-sectional observational study was conducted in 10 cities in Ethiopia—Addis Ababa, Adama, Assosa, Bahir Dar, Dire Dawa, Gambella, Harar, Hawassa, Jigjiga, and Semera-Logia—between December 5th and 28th, 2022. Data were collected electronically using smartphones, utilizing a pre-tested, standardized checklist and covert observation. The subjects were selected through multi-stage cluster sampling. A total of 1,370 HVs (hotels, restaurants, bars, bars and restaurants, café and restaurants, butcher houses and restaurants, groceries, nightclubs and lounges) were observed. Specific and composite compliance indicators were computed for indoor and outdoor spaces. Poisson regression analyses identified factors associated with indoor active smoking and non-compliance. Statistical significance was set at P<0.05. Data were analyzed using SPSS version 26.<br><br>Results<br>The average indoor compliance was 76.2% (95% CI: 75.3-77.1). Bars (60.8%) and nightclubs/lounges (57.6%) had the lowest indoor compliance rates. Indoor active smoking was observed in 32.2% of HVs, mainly in nightclubs/lounges (68.6%). About 35% of HVs displayed 'no smoking' signage indoors. Only 14.2% of indoor spaces were fully compliant. Outdoor compliance averaged at 70.5% (94% CI:68.5-72.3). Active smoking was observed in 46.5% of outdoor spaces, and only 33.1% of HVs posted 'no smoking' signage outdoors. 'No smoking' signage was significantly associated with lower indoor active smoking (adjusted prevalence ratio [adjPR] 0.76; 95% CI:0.64-0.89) and lower non-compliance (adjPR 0.43; 0.37-0.50).<br><br>Conclusions<br>The study shows satisfactory overall compliance with smoke-free laws but significant variability across cities and HV types. Enhanced enforcement in low-compliance areas like bars and nightclubs is crucial. Increased 'no smoking' signage, public education, regular monitoring, and stricter penalties are recommended. Future research should include air quality assessments to measure SHS exposure levels objectively.
Order of Authors:	Wakgari Deressa, PhD
	Selamawit Hirpa, PhD
	Terefe Gelibo Argefa
	Yifokire Tefera
	Selam Abraham Kassa, PhD

Powered by Editorial Manager® and ProduXion Manager® from Aries Systems Corporation

	Rachel Kitonyo-Devotsu, PhD
	Winnie Awuor, PhD
	Baharu Zewdie, MSc
	Noreen Dadirai Mdege, PhD
**Additional Information:**	
**Question**	**Response**
**Financial Disclosure**<br><br>Enter a financial disclosure statement that describes the sources of funding for the work included in this submission. Review the submission guidelines for detailed requirements. View published research articles from *PLOS ONE* for specific examples.<br><br>This statement is required for submission and **will appear in the published article** if the submission is accepted. Please make sure it is accurate.<br><br>**Funded studies**<br>Enter a statement with the following details:<br>• Initials of the authors who received each award<br>• Grant numbers awarded to each author<br>• The full name of each funder<br>• URL of each funder website<br>• Did the sponsors or funders play any role in the study design, data collection and analysis, decision to publish, or preparation of the manuscript?<br><br>Did you receive funding for this work?	Yes
Please add funding details.<br>as follow-up to "**Financial Disclosure**<br><br>Enter a financial disclosure statement that describes the sources of funding for the work included in this submission. Review the submission guidelines for detailed requirements. View published research articles from *PLOS ONE* for specific examples.<br><br>This statement is required for submission	This study was supported with funding from the Bill and Melinda Gates Foundation (grant number INV-009670). The findings and conclusions contained in the study are those of the authors and do not necessarily reflect the positions and policies of the donor.

and **will appear in the published article** if the submission is accepted. Please make sure it is accurate.<br><br>**Funded studies**<br>Enter a statement with the following details:<br>• Initials of the authors who received each award<br>• Grant numbers awarded to each author<br>• The full name of each funder<br>• URL of each funder website<br>• Did the sponsors or funders play any role in the study design, data collection and analysis, decision to publish, or preparation of the manuscript?<br><br>Did you receive funding for this work?"	
Please select the country of your main research funder (please select carefully as in some cases this is used in fee calculation).<br>as follow-up to "**Financial Disclosure**<br><br>Enter a financial disclosure statement that describes the sources of funding for the work included in this submission. Review the submission guidelines for detailed requirements. View published research articles from *PLOS ONE* for specific examples.<br><br>This statement is required for submission and **will appear in the published article** if the submission is accepted. Please make sure it is accurate.<br><br>**Funded studies**<br>Enter a statement with the following details:<br>• Initials of the authors who received each award<br>• Grant numbers awarded to each author<br>• The full name of each funder<br>• URL of each funder website<br>• Did the sponsors or funders play any role in the study design, data collection and analysis, decision to publish, or preparation of the manuscript?	UNITED STATES MINOR OUTLYING ISLANDS - UM

Did you receive funding for this work?"	
**Competing Interests**<br><br>Use the instructions below to enter a competing interest statement for this submission. On behalf of all authors, disclose any competing interests that could be perceived to bias this work—acknowledging all financial support and any other relevant financial or non-financial competing interests.<br><br>This statement is required for submission and **will appear in the published article** if the submission is accepted. Please make sure it is accurate and that any funding sources listed in your Funding Information later in the submission form are also declared in your Financial Disclosure statement.<br><br>View published research articles from *PLOS ONE* for specific examples.<br><br>**NO authors have competing interests**<br><br>Enter: *The authors have declared that no competing interests exist.*<br><br>**Authors with competing interests**<br><br>Enter competing interest details beginning with this statement:<br><br>*I have read the journal's policy and the authors of this manuscript have the following competing interests: [insert competing interests here]*<br><br>* typeset	The authors declare no competing interests.
**Ethics Statement**<br><br>Enter an ethics statement for this submission. This statement is required if the study involved:	The study protocol for the project titled ‘‘Compliance with Smoke-free Laws and Tobacco Advertisement, Promotion and Sponsorship Bans in Ethiopia" was approved the Institutional Review Board of the Ethiopian Public Health Association (EPHA) (Ref. no. EPHA/OG/201/22; dated November 18, 2022). Due to the covert observational nature of the study, individual informed consent from the HVs or individuals was not obtained. The observation checklists did not include names, addresses, or any other

Powered by Editorial Manager® and ProduXion Manager® from Aries Systems Corporation

• Human participants<br>• Human specimens or tissue<br>• Vertebrate animals or cephalopods<br>• Vertebrate embryos or tissues<br>• Field research<br><br>Write "N/A" if the submission does not require an ethics statement.<br><br>General guidance is provided below. Consult the submission guidelines for detailed instructions. **Make sure that all information entered here is included in the Methods section of the manuscript.**	identifying information, ensuring anonymity, privacy, and data confidentiality.

Powered by Editorial Manager® and ProduXion Manager® from Aries Systems Corporation

**Format for specific study types**<br><br>**Human Subject Research (involving human participants and/or tissue)**<br>• Give the name of the institutional review board or ethics committee that approved the study<br>• Include the approval number and/or a statement indicating approval of this research<br>• Indicate the form of consent obtained (written/oral) or the reason that consent was not obtained (e.g. the data were analyzed anonymously)<br><br>**Animal Research (involving vertebrate animals, embryos or tissues)**<br>• Provide the name of the Institutional Animal Care and Use Committee (IACUC) or other relevant ethics board that reviewed the study protocol, and indicate whether they approved this research or granted a formal waiver of ethical approval<br>• Include an approval number if one was obtained<br>• If the study involved *non-human primates*, add *additional details* about animal welfare and steps taken to ameliorate suffering<br>• If anesthesia, euthanasia, or any kind of animal sacrifice is part of the study, include briefly which substances and/or methods were applied<br><br>**Field Research**<br><br>Include the following details if this study involves the collection of plant, animal, or other materials from a natural setting:<br>• Field permit number<br>• Name of the institution or relevant body that granted permission	
**Data Availability**<br><br>Authors are required to make all data underlying the findings described fully available, without restriction, and from the time of publication. PLOS allows rare exceptions to address legal and ethical concerns. See the PLOS Data Policy and FAQ for detailed information.	Yes - all data are fully available without restriction

A Data Availability Statement describing where the data can be found is required at submission. Your answers to this question constitute the Data Availability Statement and **will be published in the article**, if accepted.<br><br>**Important:** Stating 'data available on request from the author' is not sufficient. If your data are only available upon request, select 'No' for the first question and explain your exceptional situation in the text box.<br><br>Do the authors confirm that all data underlying the findings described in their manuscript are fully available without restriction?	
**Describe where the data may be found in full sentences. If you are copying our sample text, replace any instances of XXX with the appropriate details.**<br><br>• If the data are **held or will be held in a public repository**, include URLs, accession numbers or DOIs. If this information will only be available after acceptance, indicate this by ticking the box below. For example: *All XXX files are available from the XXX database (accession number(s) XXX, XXX.).*<br>• If the data are all contained **within the manuscript and/or Supporting Information files**, enter the following: *All relevant data are within the manuscript and its Supporting Information files.*<br>• If neither of these applies but you are able to provide **details of access elsewhere**, with or without limitations, please do so. For example:<br><br>*Data cannot be shared publicly because of [XXX]. Data are available from the XXX Institutional Data Access / Ethics Committee (contact via XXX) for researchers who meet the criteria for access to confidential data.*<br><br>*The data underlying the results presented in the study are available from (include the name of the third party*	Data are available with the first author

and contact information or URL).<br>• This text is appropriate if the data are owned by a third party and authors do not have permission to share the data.<br><br>* typeset	
Additional data availability information:	Tick here if your circumstances are not covered by the questions above and you need the journal's help to make your data available.

Powered by Editorial Manager® and ProduXion Manager® from Aries Systems Corporation

Manuscript Click here to access/download;Manuscript;SFE Manuscript.docx

Compliance with smoke-free laws in hospitality venues in Ethiopia: a cross-sectional observational study in 10 cities

Wakgari Deressa[1*], Selamawit Hirpa[1], Terefe Gelibo Argefa[2,3], Yifokire Tefera[1], Selam Abraham Kassa[2], Rachel Kitonyo-Devotsu[2], Winnie Awuor[2], Baharu Zewdie[4], Noreen Dadirai Mdege[2,5,6]

[1]Department of Preventive Medicine, School of Public Health, College of Health Sciences, Addis Ababa University, Addis Ababa, Ethiopia

[2]Development Gateway: an IREX Venture, Washington DC, USA

[3]ICAP at Columbia University, Mailman School of Public Health, Addis Ababa, Ethiopia

[4]Tobacco Control Unit, Ethiopian Food and Drug Authority, Addis Ababa, Ethiopia

[5]Department of Health Sciences, University of York, York YO10 5DD, UK

[6]Centre for Research in Health and Development, York, UK

*Corresponding author (Wakgari Deressa: deressaw@gmail.com); P. O. Box 1176

Short title: Smoke-free Law Compliance in Ethiopia

Abstract

Background

The 2019 Ethiopia's comprehensive tobacco control proclamation mandates 100% smoke-free public places and workplaces. Despite the proclamation, compliance remains uncertain, particularly at hospitality venues (HVs) such as restaurants, bars, nightclubs, and hotels. The aim of this study was to evaluate the extent of compliance with smoke-free laws in HVs and identify factors associated with non-compliance.

Methods

This cross-sectional observational study was conducted in 10 cities in Ethiopia—Addis Ababa, Adama, Assosa, Bahir Dar, Dire Dawa, Gambella, Harar, Hawassa, Jigjiga, and Semera-Logia—between December 5th and 28th, 2022. Data were collected electronically using smartphones, utilizing a pre-tested, standardized checklist and covert observation. The subjects were selected through multi-stage cluster sampling. A total of 1,370 HVs (hotels, restaurants, bars, bars and restaurants, café and restaurants, butcher houses and restaurants, groceries, nightclubs and lounges) were observed. Specific and composite compliance indicators were computed for indoor and outdoor spaces. Poisson regression analyses identified factors associated with indoor active smoking and non-compliance. Statistical significance was set at P<0.05. Data were analyzed using SPSS version 26.

Results

The average indoor compliance was 76.2% (95% CI: 75.3-77.1). Bars (60.8%) and nightclubs/lounges (57.6%) had the lowest indoor compliance rates. Indoor active smoking was observed in 32.2% of HVs, mainly in nightclubs/lounges (68.6%). About 35% of HVs displayed ‘no smoking’ signage indoors. Only 14.2% of indoor spaces were fully compliant. Outdoor

compliance averaged at 70.5% (94% CI:68.5-72.3). Active smoking was observed in 46.5% of outdoor spaces, and only 33.1% of HVs posted 'no smoking’ signage outdoors. ‘No smoking’ signage was significantly associated with lower indoor active smoking (adjusted prevalence ratio [adjPR] 0.76; 95% CI:0.64-0.89) and lower non-compliance (adjPR 0.43; 0.37-0.50).

Conclusions

The study shows satisfactory overall compliance with smoke-free laws but significant variability across cities and HV types. Enhanced enforcement in low-compliance areas like bars and nightclubs is crucial. Increased 'no smoking’ signage, public education, regular monitoring, and stricter penalties are recommended. Future research should include air quality assessments to measure SHS exposure levels objectively.

Keywords: Compliance; Hospitality venue; Smoke-free laws; Smoking; Ethiopia

Introduction

According to the Global Adult Tobacco Survey (GATS) conducted in 2016 in Ethiopia, 3.7% (approximately 2.5 million) of adults (6.2% of men and 1.2% of women) were current cigarette smokers [1], and the smoking prevalence is predicted to rise [2-4]. Exposure of non-smokers to the secondhand tobacco smoke (SHS) is a major concern [5]. From Ethiopia's 2016 GATS, 31% of adults aged 15 years and older who visited restaurants, and 60% who visited bars/nightclubs, were exposed to SHS from tobacco in the 30 days preceding the survey [1]. It has been demonstrated that comprehensive smoke-free legislation effectively reduces SHS exposure among customers, bartenders and workers at hospitality venues (HVs) [6] reduces smoking among adolescents and young people [7]. However, studies in sub-Saharan Africa have indicated a lack of knowledge and low levels of compliance with smoke-free laws in HVs [8-10].

Ethiopia enacted a new comprehensive tobacco control law in 2019 (Proclamation No.1112/2019) [11]. The proclamation requires 100% smoke-free indoor public places and workplaces including HVs. Smoking and the use of tobacco products is prohibited in all indoor workplaces, public places, public institutions, modes of transportation, and communal areas. The proclamation also prohibits designated smoking areas (DSAs) in public places, as well as smoking or tobacco use within 10 meters of any public place or workplace doorway, window, or air intake mechanism. Furthermore, the proclamation mandates the owner of the public place or another authorized person to post a 'no smoking' signage and prohibit tobacco use in any part of indoor workplaces, and indoor hospitality settings.

A recent scoping review on SHS and smoke-free environments in Ethiopia identified only two studies that assessed compliance with smoke-free laws [12]. One of the two studies only studied five government hospitals in Addis Ababa [13] and was conducted prior to the enactment of

Proclamation No.1112/2019 [11]. The second study was conducted in 2021 in public places such as those providing food and beverages, schools, health facilities, government offices, youth centers, parks and transit facilities in four regions [10].

Studies indicate a high prevalence of indoor smoking in HVs [8, 14, 15], with workers in bars and nightclubs experiencing higher levels of SHS exposure [16, 17] and a greater risk of lung cancer mortality [18]. Compliance is a key pillar of tobacco control policy implementation; however, there is a scarcity of evidence on the extent of compliance with smoke-free laws in Ethiopia, particularly in HVs. Therefore, we conducted an assessment of compliance with smoke-free laws in HVs in Ethiopia to provide decision-makers with the evidence they need to strengthen their efforts in protecting non-smokers from exposure to SHS. In this study, HV is defined as an establishment registered under the regulation of the Government of Ethiopia where food and beverages are sold and consumed, namely hotels, restaurants, bars, bars and restaurants, café and restaurant, butcher shop and restaurant, grocery, nightclub and lounges.

Methods

Study design, setting and population

This cross-sectional study used covert observations to determine compliance with smoke-free laws at HVs in 10 purposively selected major regional and chartered cities in Ethiopia: Addis Ababa, Adama, Assosa, Bahir Dar, Dire Dawa, Gambella, Harar, Hawassa, Jigjiga, and Semera-Logia (where Semera and Logia are two separate nearby cities merged as one study area). The selected cities are characterized by diverse demographic and geographic features, high population density, fast economic development, and high smoking prevalence [1]. The Tigray Regional State was not included in the study because of security problem at the time of the study.

Sample size and sampling procedures

We estimated the total minimum sample size required for the study using a single proportion sample size formula. We considered a study conducted in Uganda, which found that 82% of the surveyed HVs complied with 'no active smoking' in the venues [8]. Based on 95% confidence level, 3% margin of error, design effect of two, and 5% non-response rate, a minimum of 1300 HVs were required for the study. The sample size was allocated to the study cities and the selected sub-cities, woredas and kebeles within them proportionally to the estimated number of the HVs.

Our sampling procedures followed recommendations for studies on compliance with smoke-free laws [19]. Four of the 10 cities (Addis Ababa, Adama, Bahir Dar and Hawassa) are sub-divided into sub-cities, which are further sub-divided into woredas/kebeles. Two to six sub-cities, with the highest density of registered HVs, were selected from the list of sub-cities in each of these four cities in consultation with the city's tobacco control law enforcement team (S1 File). Similarly, from the list of kebeles in each selected sub-city in Adama, Bahir Dar, and Hawassa, two kebeles with the highest density of registered HVs were chosen. In the remaining cities, i.e., Jigjiga, Semera-Logia, Dire Dawa, Harar, Assosa and Gambella, where there are no sub-cities/woredas, we selected 2-6 kebeles/woredas, focusing on those with the highest concentration of HVs in each city.

We obtained the list of registered HVs in each selected kebele/woreda from the relevant offices. However, due to data incompleteness, we supplemented these lists by walking from street to street within the selected kebele/woreda, mapping, and listing existing HVs. We used these lists to purposively select 2-4 neighborhoods (or villages), each consisting of multiple streets and having the highest density of HVs, from each participating kebele/wodera as sites for data collection. To ensure representation of various categories of HVs within the chosen kebele/woreda, the assigned

sample size for each kebele/woreda was distributed proportionally across the different types of HVs.

Eligibility criteria

All HVs chosen for this study and providing services during the time of data collection were included in the study.

Data collection tools and procedures

This study used a standardized observation checklist to guide covert observations. The observation checklist was developed based on the “How-to-Guide for Conducting Compliance Studies” for Smoke-Free Law [19]. We adapted the checklist considering the provisions of the Ethiopian Tobacco Control Proclamation (1112/2019) [11] and the 2021 tobacco control directive [20]. The tool was initially developed in English, then translated into Amharic, and back-translated into English to ensure consistency. The data collection tool (S2 File) was reviewed and enriched by tobacco control experts in Ethiopia, including those from the Ethiopian Food and Drug Authority (EFDA).

Data were collected by 24 trained data collectors, with one pair of data collectors in each city, except for Addis Ababa, which had three pairs of data collectors. There were 10 field supervisors, one for each city. A three-day training was held in Addis Ababa for all data collectors and supervisors. The data were collected electronically using Open Data Kit (ODK) between December 5th and 28th, 2022, during peak business hours (17:00-24:00).

After scanning the outdoor area and entering the selected HV, the data collectors took a seat as customers, preferably in the main room. In some HVs, they also ordered beverages or food. The

specific areas of the HVs that were covertly observed included main entrance, porch, main room, and two additional rooms, depending on the size of the venue. Other areas observed were lobby areas, corridor areas, hand washing and toilet areas. The data collectors entered the data into the smartphone/tablet while they were in or near the HVs without the knowledge of the HV owners or staff members. This approach improved the reliability and validity of the collected data [21].

Smoke-free compliance indicators

Indoor space compliance: The following seven smoke-free specific indicators were used to assess the indoor space compliance: 1) no one was seen smoking tobacco product in the venue's indoor space (0=no, 1=yes), 2) no ashtrays or other instruments used to hold cigarette ash inside (0=no, 1=yes), 3) no lighter visible inside (0=no, 1=yes), 4) display of 'no smoking' signage seen (0=no, 1=yes), 5) absence of designated smoking area (DSA) (0=no, 1=yes), 6) absence of shisha equipment seen (0=no, 1=yes), 7) nobody used tobacco products in the outdoor place within 10 meters from any door, window, or air intake mechanism (0=no, 1=yes).

Outdoor space compliance: The following four smoke-free specific indicators were used to assess the outdoor space compliance: 1) no one was seen smoking tobacco product in the venue's outdoor space (0=no, 1=yes), 2) no ashtray or other instrument used to hold cigarette ash in the outdoor space (0=no, 1=yes), 3) display of 'no smoking' signage in the outdoor space (0=no, 1=yes), and 4) absence of DSA in the outdoor space (0=no, 1=yes).

Data quality control

Field personnel received training, and the checklists underwent pre-testing to ensure accuracy and reliability. Data were collected electronically using ODK. Field supervisors reviewed and checked at least 5% of the data collected by each data collector. Supervisors were actively involved in data

collection alongside collectors, resolving any inconsistencies through discussions. Each day, collected data were uploaded to a central server, where they underwent daily reviews for completeness and consistency, with immediate feedback from the data manager.

Operational definitions

Active smoking means someone being in possession or control of a lit tobacco product, including cigarettes, cigars, and shisha, inside or outside of HVs at the time of data collection. Compliance refers to the degree to which the HVs fully implementing tobacco control laws under the 2019 Ethiopian Tobacco Control Law (Proclamation No.1112/2019). Further operational definitions used in this study are attached in S3 File.

Data processing and analysis

Descriptive analysis was used to summarize the characteristics of the HVs, as well as compliance estimates for each compliance indicator. We computed the overall average compliance with the smoke-free laws across all HVs by adding the overall compliance estimates for the seven compliance indicators together and dividing the sum by seven. This method, previously employed in tobacco control compliance studies [22-24], was used to determine the average compliance.

We also classified each HV as 'fully compliant', 'good compliance', 'moderately compliant', 'poorly compliant' with smoke-free laws. An HV was categorized as 'fully compliant' for indoor space if it complied with all seven indicators, 'good compliance' for complying with 5-6 indicators, 'moderately compliant' for complying with 3-4 indicators, and 'poor compliance' if it met ≤ 2 indicators. For outdoor areas, we summed up the overall compliance estimates for the four compliance indicators and divided the sum by four to obtain overall outdoor compliance. An HV

was classified as 'fully compliant' for outdoor area if it complied with all four indicators, 'good compliance' if it met three indicators, and 'poor compliance' if it only complied with ≤2 indicators.

Sub-analyses were carried out by study city and HV types. Continuous variables were described using means and standard deviations, while categorical variables were characterized using frequencies and proportions. The study investigated factors such as city, type of HVs, and the presence of 'no smoking' signage associated with indoor active smoking (yes/no) and 'below-average indoor compliance' with smoke-free laws (dependent variables) using Poisson regression analyses with log link function and robust variance [25] and calculated adjusted prevalence ratios (adjPR) with 95% confidence intervals (CIs).

The average compliance value was used as a cut-off point to classify HV into categories of above or below average compliance. Values falling below the average indicate below-average compliance, whereas those above the average show above-average compliance. Collinearity between cities and HV types was assessed by calculating the Variance Inflation Factor (VIF), and no evidence of multicollinearity was found (VIF < 2). We calculated standard errors, taking into account the complex sample design, which involved adjusting for stratification by city and clustering of HVs at the kebele/cluster level. $P<0.05$ was used for statistical significance. Data were analyzed with SPSS version 26 (IBM SPSS Statistics for Windows, Armonk, NY, USA).

Ethics considerations

The study protocol for the project titled ‘‘Compliance with Smoke-free Laws and Tobacco Advertisement, Promotion and Sponsorship Bans in Ethiopia” was approved the Institutional Review Board of the Ethiopian Public Health Association (EPHA) (Ref. no. EPHA/OG/201/22; dated November 18, 2022). Due to the covert observational nature of the study, individual informed consent from the HVs or individuals was not obtained. The observation checklists did not include names, addresses, or any other identifying information, ensuring anonymity, privacy, and data confidentiality.

Results

Characteristics of the HVs

Data were collected from a total of 1,370 HVs in 10 cities. About 76% (n=1,041) of the 1,370 HVs only had indoor spaces, whereas 23.9% (n=327) had both indoor and outdoor spaces, while two (0.1%) had only outdoor areas. Addis Ababa had the highest number of venues (20.8%), followed by Adama (11.4%), Hawassa (11.3%), and Bahir Dar (10.7%) (Table 1). The remaining six cities were represented by between 7.4% and 7.9% of the HVs. Hotels accounted for 19.3% of all venues, followed by bars and restaurants (17.4%), cafés and restaurants (14.8%), and restaurants (14.7%).

Table 1. Types of hospitality venues by city

City	Hospitality venue, n (%)								Total, n (%)
	Hotel	Bar & restaurant	Café & restaurant	Restaurant	Grocery	Butcher house & restaurant	Bar	Nightclub /lounge	
**Addis Ababa**	27 (9.5)	73 (25.6)	37 (13.0)	42 (14.8)	30 (10.5)	4 (1.4)	45 (15.8)	27 (9.5)	285 (20.8)
**Adama**	28 (17.9)	38 (24.4)	32 (20.5)	10 (6.4)	16 (10.3)	22 (14.1)	7 (4.5)	3 (1.9)	156 (11.4)
**Hawassa**	23 (14.9)	53 (34.4)	17 (11.0)	18 (11.7)	21 (13.6)	1 (0.6)	14 (9.1)	7 (4.5)	154 (11.3)
**Bahir Dar**	21 (14.3)	9 (6.1)	17 (11.6)	38 (25.9)	19 (12.9)	13 (8.8)	18 (12.2)	12 (8.2)	147 (10.7)
**Jigjiga**	46 (42.6)	0	22 (20.4)	22 (20.4)	7 (6.5)	11 (10.2)	0	0	108 (7.9)
**Semera-Logia**	23 (21.3)	1 (0.9)	47 (43.5)	37 (34.3)	0	0	0	0	108 (7.9)
**Harar**	14 (13.3)	11 (10.5)	19 (18.1)	0	4 (3.8)	49 (46.7)	2 (1.9)	6 (5.7)	105 (7.7)
**Dire Dawa**	18 (17.5)	21 (20.4)	2 (1.9)	5 (4.9)	29 (28.2)	8 (7.8)	12 (11.7)	8 (7.8)	103 (7.5)
**Assosa**	33 (32.0)	15 (14.6)	8 (8.8)	4 (3.9)	25 (24.3)	10 (9.7)	1 (1.0)	7 (6.8)	103 (7.5)
**Gambella**	31 (30.7)	17 (16.8)	3 (3.0)	25 (24.8)	16 (15.8)	6 (5.9)	3 (3.0)	0	101 (7.4)
**Total, n (%)**	**264 (19.3)**	**238 (17.4)**	**204 (14.8)**	**201 (14.7)**	**167 (12.2)**	**124 (9.0)**	**102 (7.4)**	**70 (5.1)**	**1,370 (100.0)**

Indoor active smoking

Active smoking was observed in indoors of 441 [32.2% (95% CI:30-35)] HVs, with 97.3% (n=430) from cigarettes. Shisha use was rare (4.8%) in the indoor spaces, mainly in nightclubs/lounges. Active smoking was the highest in the indoor spaces of HVs in Semera-Logia (70.4%) and Dire Dawa (61.2%). The highest rates of observed active smoking were in the nightclubs/lounges (68.6%) and bars (65.7%). The proportion of HVs that posted 'no smoking

signage' in indoor spaces was 35.2% (95% CI:33.0-38.0), with the highest proportion in Semera-Logia (66.7%), whilst this was lowest in Jigjiga (8.3%).

Compliance with smoke free laws in indoor spaces

The overall average indoor compliance with smoke-free laws in HVs based on all seven indicators was 76.2% (95% CI: 75.3-77.1), with 23.8% non-compliance (Table 2). Overall average compliance in indoor spaces was the highest in Jigjiga (83.9%), followed by Addis Ababa (79.4%), Harar (78.8%), Adama (78.5%), and the lowest was observed in Dire Dawa (66.6%). The highest level of compliance with smoke-free laws in indoor spaces was found in cafés and restaurants (83.8%). Bars (60.8%) and nightclubs/lounges (57.6%), on the other hand, had the lowest average compliance for indoor spaces. There were almost no DSAs (98.2%) and no shisha equipment (98.0%) observed in indoor spaces of the venues across the cities.

Table 2. Compliance with smoke-free laws in indoor spaces by cities and HVs

City (n=1368)	Smoke-free compliance indicator, n (%)							Average** compliance, %
	**No active smoking**	**No ashtray**	**No lighter**	**'No smoking' signage**	**No DSA***	**No shisha equipment**	**No one used tobacco products within 10m of any air intake mechanism**	
Jigjiga (n=108)	100 (92.6)	108 (100.0)	108 (100.0)	9 (8.3)	108 (100.0)	108 (100.0)	93 (86.1)	**83.9**
Addis Ababa (n=284)	209 (73.6)	271 (95.4)	255 (89.8)	152 (53.5)	284 (100.0)	276 (97.2)	131 (46.1)	**79.4**
Harar (n=105)	70 (66.7)	104 (99.0)	105 (100.0)	57 (54.3)	105 (100.0)	105 (100.0)	33 (31.4)	**78.8**
Adama (n=156)	117 (75.0)	145 (92.9)	148 (94.9)	50 (32.4)	143 (91.7)	154 (98.7)	100 (64.1)	**78.5**
Bahir Dar (n=146)	113 (77.4)	137 (93.8)	130 (89.0)	19 (13.0)	143 (97.9)	141 (96.6)	111 (76.0)	**77.7**
Gambella (n=101)	65 (63.7)	94 (93.1)	89 (88.1)	16 (15.8)	101 (100.0)	99 (98.0)	61 (60.4)	**74.3**
Assosa (n=103)	69 (67.0)	103 (100.0)	90 (87.4)	33 (32.0)	102 (99.0)	102 (99.0)	31 (30.4)	**73.5**
Hawassa (n=154)	112 (72.7)	141 (91.6)	121 (78.6)	45 (29.2)	152 (98.7)	149 (96.8)	68 (44.2)	**73.1**
Semera-Logia (n=108)	32 (29.6)	95 (88.0)	106 (98.1)	72 (66.7)	102 (94.4)	107 (99.1)	16 (14.8)	**70.1**
Dire Dawa (n=103)	40 (38.8)	99 (96.1)	68 (66.0)	28 (26.9)	103 (100.0)	99 (96.1)	43 (41.7)	**66.6**
P-value	<0.001	<0.001	<0.001	<0.001	<0.001	0.260	<0.001	**<0.001**
**Hospitality venue (n=1368)**								
Café & restaurant (n=204)	166 (81.4)	198 (97.1)	201 (98.5)	87 (42.6)	204 (100.0)	204 (100.0)	137 (67.2)	**83.8**
Hotel (n=264)	181 (68.6)	254 (96.2)	241 (91.3)	124 (47.0)	253 (95.8)	262 (99.2)	147 (55.7)	**79.1**
Butcher house & restaurant (n=123)	94 (76.4)	120 (97.6)	121 (98.4)	36 (29.3)	123 (100.0)	123 (100.0)	63 (51.2)	**79.0**
Restaurant (n=201)	148 (73.6)	190 (94.5)	195 (97.0)	52 (25.9)	198 (98.5)	201 (100.0)	113 (56.2)	**78.0**
Bar & restaurant (n=237)	173 (73.0)	229 (96.6)	216 (91.1)	96 (40.5)	233 (98.3)	234 (98.7)	112 (47.2)	**77.9**
Grocery (n=167)	108 (64.7)	162 (97.0)	142 (85.0)	36 (21.6)	166 (99.4)	166 (99.4)	70 (41.9)	**72.7**
Bar (n=102)	35 (34.7)	85 (83.3)	62 (61.4)	30 (29.7)	99 (97.1)	94 (92.2)	29 (28.7)	**60.8**
Nightclub/lounge (n=70)	22 (31.4)	59 (84.3)	42 (60.0)	20 (28.6)	67 (95.7)	56 (80.0)	16 (22.9)	**57.6**
P-value	<0.001	<0.001	<0.001	<0.001	0.009	<0.001	<0.001	**<0.001**
Overall compliance, n (%)	**927 (67.8)**	**1297 (94.8)**	**1220 (89.2)**	**481 (35.2)**	**1343 (98.2)**	**1340 (98.0)**	**687 (50.2)**	**76.2**

**DSA - Designated Smoking Area; **The overall average compliance was calculated by adding up the values of 'specific compliance indicators' and dividing them by the total number of indicators.*

Only 14.2% of the indoor areas were fully compliant, and full compliance was the highest in Addis Ababa (22.5%) (Table 3).

Table 3. Indoor compliance with smoke-free laws by cities and HVs

City	Indoor compliance, n (%)*			
	**Fully compliant**	**Good**	**Moderate**	**Poor**
Addis Ababa	64 (22.5)	164 (57.7)	48 (16.9)	8 (2.8)
Adama	29 (18.6)	96 (61.5)	25 (16.0)	6 (3.8)
Hawassa	28 (18.2)	81 (52.6)	36 (23.4)	9 (5.8)
Bahir Dar	8 (5.5)	112 (76.7)	24 (16.4)	2 (1.4)
Jigjiga	9 (8.3)	95 (88.0)	4 (3.7)	0
Semera-Logia	8 (7.4)	61 (56.5)	37 (34.3)	2 (1.9)
Dire Dawa	9 (8.7)	47 (45.6)	43 (41.7)	4 (3.9)
Harar	16 (15.2)	71 (67.6)	18 (17.1)	0
Assosa	15 (14.6)	61 (59.2)	26 (25.2)	1 (1.0)
Gambella	8 (7.9)	69 (68.3)	23 (22.8)	1 (1.0)
**Hospitality venue**				
Restaurant	19 (9.5)	149 (74.1)	32 (15.9)	1 (0.5)
Café & restaurant	54 (26.5)	128 (62.7)	22 (10.8)	0
Bar & restaurant	40 (16.9)	154 (65.0)	39 (16.5)	4 (1.7)
Hotel	51 (19.3)	160 (60.6)	50 (18.9)	3 (1.1)
Grocery	10 (6.0)	107 (64.1)	48 (28.7)	2 (1.2)
Butcher house & restaurant	14 (11.4)	91 (74.0)	18 (14.6)	0
Bar	5 (4.9)	42 (41.2)	42 (41.2)	13 (12.7)
Nightclub/lounge	1 (1.4)	26 (37.1)	33 (47.1)	10 (14.3)
**Total, n (%)**	**194 (14.2)**	**857 (62.6)**	**284 (20.8)**	**33 (2.4)**

**Fully compliant: compliance with seven indicators; Good compliance: compliance with 5-6 indicators; Moderate compliance: compliance with 3-4 indicators; Poor compliance: compliance with one or two indicators.*

Outdoor active smoking

Active smoking was observed in the outdoor space of 46.5% (95% CI:41.0-52.0) of the venues; and this was true for 65.7% of HVs in Semera-Logia and 51.6% of HVs in Adama, compared to 37.9% of HVs in Addis Ababa and 13.8% of HVs in Jigjiga. Active smoking in outdoor spaces was observed in 71.4% of the bars, 57.1% of the nightclubs/lounges, and 56.9% the restaurants. 'No smoking' signage was observed in the outdoor spaces of 33.1% (95% CI:28.0-38.0) of the

HVs, with the highest proportions found in Semera-Logia (59.3%) and Addis Ababa (44.8%). Bars, groceries, nightclubs/lounges, and butcher shops and restaurants had the lowest rates of ‘no smoking’ signage. The presence of ashtrays (1.2%) and DSAs (3.6%) was very low in outdoor spaces.

Compliance with smoke-free laws in outdoor spaces

The overall average compliance with smoke-free laws in outdoor areas of HVs was 70.4% (95% CI:68.5-72.3), and this ranged from 66.2% in Gambella to 77.1% in Bahir Dar (Table 4). Bars (57.14%), nightclub/lounge (64.3%) had the lowest total average outdoor compliance compared to hotels (71.3%), and cafés and restaurants (76.2%) (p=0.041).

Table 4. Compliance with smoke-free laws in outdoor spaces by cities and HVs

City (n=329)	Outdoor smoke-free compliance indicator, n (%)				Average** compliance, %
	No ashtray	No DSA*	No active smoking	'No smoking' signage	
Bahir Dar (n=12)	12 (100.0)	12 (100.0)	12 (100.0)	1 (8.3)	**77.1**
Addis Ababa (n=29)	27 (96.4)	29 (100.0)	18 (62.3)	13 (44.8)	**75.0**
Assosa (n=21)	21 (100.0)	21 (100.0)	12 (57.8)	9 (42.9)	**75.0**
Semera-Logia (n=108)	107 (99.1)	106 (98.1)	37 (34.3)	64 (59.3)	**72.7**
Jigjiga (n=29)	29 (100.0)	29 (100.0)	25 (86.2)	0	**71.6**
Hawassa (n=37)	36 (97.3)	34 (91.9)	21 (56.8)	9 (24.3)	**67.6**
Dire Dawa (n=32)	32 (100.0)	32 (100.0)	18 (56.3)	3 (9.4)	**66.4**
Gambella (n=17)	17 (100.0)	15 (88.2)	11 (64.7)	2 (11.8)	**66.2**
Harar (n=13)	13 (100.0)	13 (100.0)	7 (53.8)	1 (7.7)	**65.4**
Adama (n=31)	31 (100.0)	26 (83.9)	15 (48.4)	7 (22.6)	**63.7**
P-value	0.844	0.003	<0.001	<0.001	**0.057**
**Hospitality venue (n=329)**					
Café & restaurant (n=63)	63 (100.0)	62 (98.4)	34 (54.0)	33 (52.4)	**76.2**
Hotel (n=102)	100 (98.0)	95 (93.1)	58 (56.9)	38 (37.3)	**71.3**
Butcher house & restaurant (n=21)	21 (100.0)	21 (100.0)	13 (61.9)	3 (10.0)	**69.1**
Restaurant (n=58)	57 (98.3)	57 (98.3)	25 (43.1)	21 (36.2)	**69.0**
Bar & restaurant (n=51)	51 (100.0)	48 (94.1)	29 (58.0)	12 (23.5)	**68.1**
Grocery (n=20)	20 (100.0)	20 (100.0)	12 (60.0)	1 (5.0)	**66.3**
Nightclub/lounge (n=7)	7 (100.0)	7 (100.0)	3 (42.9)	1 (14.3)	**64.3**
Bar (n=7)	7 (100.0)	7 (100.0)	2 (28.6)	0	**57.1**
P-value	0.942	0.411	0.514	<0.001	**0.041**
Overall compliance, n (%)	***325 (98.8)***	***317 (96.4)***	***176 (53.5)***	***109 (33.1)***	***70.4***

**DSA - Designated Smoking Area; **The overall average compliance was calculated by adding up the values of 'specific compliance indicators' and dividing them by the total number of indicators*

About 16.0% of outdoor spaces in the HVs were fully compliant, whereas about one-third (32.8%) were found to be in poor compliance with smoke-free laws (Table 5).

Table 5. Outdoor compliance with smoke-free laws by cities and HVs

City	Outdoor compliance, n (%)*		
	**Fully compliant**	**Moderate**	**Poor**
Addis Ababa	8 (27.6)	13 (44.8)	8 (27.6)
Adama	3 (9.7)	11 (35.5)	17 (54.8)
Hawassa	6 (16.2)	15 (40.5)	16 (43.2)
Bahir Dar	1 (8.3)	11 (91.7)	0
Jigjiga	0	25 (86.2)	4 (13.8)
Semera-Logia	24 (22.2)	52 (48.1)	32 (29.6)
Dire Dawa	2 (6.3)	17 (53.1)	13 (40.6)
Harar	0	8 (61.5)	5 (38.5)
Assosa	7 (33.3)	7 (33.3)	7 (33.3)
Gambella	1 (5.9)	10 (58.8)	6 (35.3)
**Hospitality venue**			
Restaurant	8 (13.8)	29 (50.0)	21 (36.2)
Café & restaurant	15 (23.8)	37 (58.7)	11 (17.5)
Bar & restaurant	7 (13.7)	25 (49.0)	19 (37.3)
Hotel	18 (17.6)	51 (50.0)	33 (32.4)
Grocery	1 (5.0)	11 (55.0)	8 (40.0)
Butcher house & restaurant	2 (9.5)	12 (57.1)	7 (33.3)
Bar	0	2 (28.6)	5 (71.4)
Nightclub/lounge	1 (14.3)	2 (28.6)	4 (57.1)
**Total, n (%)**	**52 (15.8)**	**169 (51.4)**	**108 (32.8)**

**Fully compliant: compliance with four indicators; Moderate compliance: compliance with three indicators;*
Poor compliance: compliance with one or two indicators.

Factors associated with indoor smoking and below-average compliance

Table 6 shows the association between the independent variables (city, HV type, and presence of 'no smoking' signage) with indoor active smoking and below-average indoor compliance using multivariable Poisson regression model. Compared to HVs in Addis Ababa, those in Semera-Logia (adjusted prevalence ratio (adjPR) 5.73; 95% CI: 4.37-7.51) and Dire Dawa (adjPR 2.10; 95% CI:1.65-2.68) had significantly higher prevalence of indoor smoking. Bars and nightclubs/lounges had approximately six times higher prevalence of active smoking than cafés and restaurants. Similarly, bars, nightclubs/lounges had significantly higher below-average indoor compliance than cafés and restaurants. HVs with 'no smoking' signage posted showed significantly lower indoor smoking (adjPR 0.76; 95% CI:0.64-0.89) and had lower rates of below-average compliance (adjPR 0.43; 95% CI:0.37-0.50).

Table 6. Multivariable Poisson regression predictors of 'active smoking' and 'below-average indoor compliance'

	Indoor active smoking		Below average indoor compliance	
**City**	**Adjusted Prevalence Ratio (95% CI)***	**p-value**	**Adjusted Prevalence Ratio (95% CI)***	**p-value**
Addis Ababa	1.00 (Reference)		1.00 (Reference)	
Adama	1.24 (0.90-1.67)	0.185	1.07 (0.85-1.35)	0.561
Hawassa	1.08 (0.80-1.46)	0.600	1.22 (1.01-1.46)	0.035
Bahir Dar	0.85 (0.62-1.18)	0.344	0.79 (0.63-0.99)	0.048
Jigjiga	0.40 (0.20-0.81)	0.011	0.47 (0.31-0.73)	0.001
Semera-Logia	5.73 (4.37-7.51)	<0.001	3.59 (2.89-4.48)	<0.001
Dire Dawa	2.10 (1.65-2.68)	<0.001	1.38 (1.14-1.68)	0.001
Harar	1.78 (1.29-2.45)	<0.001	1.52 (1.20-1.93)	0.001
Assosa	1.40 (1.01-1.94)	0.047	1.70 (1.39-2.08)	<0.001
Gambella	1.65 (1.19-2.28)	0.003	1.45 (1.16-1.80)	0.001
**Hospitality venue**				
Café & restaurant	1.00 (Reference)		1.00 (Reference)	
Hotel	2.37 (1.78-3.15)	<0.001	1.92 (1.52-2.43)	<0.001
Butcher house & restaurant	1.82 (1.20-2.76)	0.004	1.84 (1.38-2.44)	<0.001
Restaurant	1.55 (1.15-2.10)	<0.001	1.59 (1.26-2.01)	<0.001
Bar & restaurant	2.35 (1.68-3.29)	<0.001	2.10 (1.64-2.68)	<0.001
Grocery	2.85 (2.01-4.03)	<0.001	2.23 (1.74-2.83)	<0.001
Bar	6.09 (4.47-8.29)	<0.001	3.84 (3.03-4.87)	<0.001
Nightclub/lounge	6.02 (4.40-8.24)	<0.001	3.41 (2.67-4.35)	<0.001
**'No smoking' signage posted (yes/no)**	0.76 (0.64-0.89)	0.001	0.43 (0.37-0.50)	<0.001
Intercept	0.075 (0.054-0.104)	<0.001	0.101 (0.077-0.131	<0.001

*CI:Confidence

Discussion

Our study found an average indoor compliance with smoke-free laws in 76.2% of the HVs, with 14.2% fully compliant, 62.6% good compliance, 20.8% moderately compliant, and 2.4% poor compliance. The average outdoor compliance was 70.4%, with 15.8% fully compliant, and about one-third (32.8%) poorly compliant. Active smoking was observed in 32.2% of indoor spaces and 46.5% of outdoor spaces. The presence of 'no smoking' signage was 35.2% in indoors and 33.1% in outdoors. Bars and nightclubs/lounges had the lowest compliance.

These findings align with a study recently conducted in 12 towns in Ethiopia showing 12.3% full compliance in public places [10]. It is worth noting that the other study [10] assessed public places different from those in our study, such as government buildings, educational facilities, health care facilities, local drinking houses, bus terminals, and transport stations. Studies also identified variability in full compliance rates across institution types [10]. For instance, Ghana reported a 70% compliance rate [26], while Bangladesh showed 58.1% indoors and 27.4% outdoors [24]. Nepal [23] and India [22] had a compliance rate of 26.3% and 80.3% in HVs, respectively.

Our study also showed regional differences in compliance within Ethiopia, with higher rates in cities like Addis Ababa, Jigjiga, Adama, and Harar, and lower in Dire Dawa and Semera-Logia. These regional differences could be attributed, in part, to the local tobacco control environment, such as different levels of implementation of the smoke-free law. This suggests varying local tobacco control environments and highlights the need for enhanced implementation and enforcement of smoke-free laws.

Indoor active smoking was observed in 32.2% of HVs, higher than a previous study conducted in Ethiopia in 2021, where active smoking was observed in 7% of the bars, restaurants, and cafés,

and in 10% of hotels [10]. This discrepancy could be due to different observation times and locations. A study in Ghana found active smoking in 34.5% of restaurants and 63.6% of bars and pubs [14], while in Uganda, active smoking was 18% in HVs [8]. Thus, implementation and enforcement of smoke-free laws, particularly in HVs, differ from area to area or across HV types within a country, as well as from country to country.

The higher prevalence of outdoor smoking suggests that smokers may be aware of indoor smoking bans and shift to outdoor spaces. Another possible explanation is that if one or more HV customers are uncomfortable by the smell of cigarette smoke from someone else in indoors, HV managers may encourage smokers to smoke outside. Bars and nightclubs/lounges had the highest rates of indoor and outdoor smoking. Effective enforcement is possible, as seen in Punjab, India, with a 98% compliance rate [22]. The presence of ‘no smoking’ signage was low in our study (35% in indoors and 33% in outdoor spaces), but higher than a previous study in Ethiopia, where just 17% of HVs and 29% of hotels posted ‘no smoking’ signage [10]. Ghana reported 49.5% signage presence [14], with similar findings in other studies [22, 23]. Our findings confirm that ‘no smoking’ signage was associated with lower indoor smoking rates and higher compliance.

The findings of this study have significant policy implications for tailored enhancement of tobacco control efforts in Ethiopia, especially in cities and HVs with lower compliance. The observed variability in compliance across different cities and types of HVs indicates the need to consider local contexts. Strengthening the implementation of Proclamation No. 1112/2019 [11] and Directive No. 771/2021 [20] is crucial. Increasing the visibility and enforcement of ‘no smoking’ signage could be an effective policy measure. Future research should include objective air quality measurements to assess secondhand smoke exposure. This could involve using objective

particulate matter ($PM_{2.5}$) concentration measurements instead of relying solely on subjective assessments to study SHS exposure [27, 28].

Strengths and limitations

This study is the first large-scale investigation of HVs across 10 major cities in Ethiopia, ensuring diverse context representations. The use of covert observations enhanced data authenticity, providing accurate compliance assessments. The inclusion of multiple indoor and outdoor compliance indicators offers a detailed understanding of compliance levels, from visible 'no smoking' signage to absence of smoking activities. However, the study had limitations. Incomplete lists of registered HVs may have led to sampling bias, and the cross-sectional design limits causality conclusions. Despite these limitations, the study provides critical baseline data and highlights areas needing targeted efforts for improving compliance with smoke-free laws.

Conclusions

While there is good compliance with smoke-free laws in Ethiopian HVs, inconsistencies remain, particularly in cities, bars, and lounges/nightclubs. Stronger enforcement, targeted interventions, and public health campaigns are required to educate the public and HV owners on SHS risks and tobacco control laws, alongside enhanced enforcement of 'no smoking' signage. Regular compliance monitoring and stricter penalties for non-compliance are essential. Additionally, expanding the focus to informal venues could enhance compliance understanding. These insights are pivotal for shaping future tobacco control policies and fostering smoke-free environments in Ethiopia.

Acknowledgements

We would like to thank the study team members from Addis Ababa University School of Public Health, Development Gateway: An IREX Venture, Tobacco Control Data Initiative Ethiopia, Ethiopian Food and Drug Authority, and Regional Tobacco Control Regulatory Offices for their valuable support in this study. We are also grateful to all of our data collectors, supervisors, and coordinators who participated in this study.

Author contributions

Conceptualization: SAK, TGA, NM, RKD, WA, WD, SH

Data curation: WD, SH, TGA

Formal analysis: WD

Funding acquisition: SAK, TGA, NM, RKD, WA

Investigation: WD, SH. YT

Methodology: WD, SH, TGA, SAK NM, RKD, WA, YT, BZ

Project administration: WD, SH

Supervision: WD, SH, YT, BZ

Writing – original draft: WD

Writing – review and editing: WD, SH, NM, TGA, YT, RKD, WA, SAK, BZ.

Funding

This study was supported with funding from the Bill and Melinda Gates Foundation (grant number INV-009670). The findings and conclusions contained in the study are those of the authors and do not necessarily reflect the positions and policies of the donor.

Data availability statement

Data is provided within the manuscript or supplementary information files. In addition, the full datasets used and analyzed in the current study are available from the corresponding author upon reasonable request.

Consent for publication

Not applicable

Competing interests

The authors declare no competing interests.

References

1. Ethiopian Public Health Institute. Global Adult Tobacco Survey: Country Report 2016. 2016. Addis Ababa, Ethiopia.
2. Defar A, Getachew T, Teklie H, et al. Tobacco use and its predictors among Ethiopian adults: A further analysis of Ethiopian NCD STEPS survey-2015. Ethiopian J Health Dev. 2017;31(Special Issue):331-339.
3. Central Statistical Agency and ICF International. Ethiopia Demographic and Health Survey 2016. Addis Ababa, Ethiopia.
4. Mengesha SD, Teklu KT, Weldetinsae A, et al. Tobacco use prevalence and its determinate factor in Ethiopia- finding of the 2016 Ethiopian GATS. *BMC Public Health* 2022; **22:**555. https://doi.org/10.1186/s12889-022-12893-8.
5. World Health Organization. Guidelines for implementation of Article 8 of the WHO Framework Convention on Tobacco Control. WHO, 2013. Geneva, Switzerland.
6. Garritsen HH, da Costa Senior YY, Rozema AD, Kunst AE, Kuipers MAG. Association Between Smoke-Free Legislation in Hospitality Venues and Smoking Behavior of Young People: A Systematic Review. Nicotine Tob Res. 2022;24(6):807-812. doi: 10.1093/ntr/ntab129.
7. Hawkins SS, Bach N, Baum CF. Impact of Tobacco Control Policies on Adolescent Smoking. J Adolesc Health. 2016;58(6):679-85. doi: 10.1016/j.jadohealth.2016.02.014.
8. Gravely, S., Nyamurungi, K. N., Kabwama, S. N., Okello, G., Robertson, L., Heng, K., Ndikum, A. E., Oginni, A. S., Rusatira, J. C., Kakoulides, S., Huffman, M. D., Yusuf, S., & Bianco, E. (2018). Knowledge, opinions and compliance related to the 100% smoke-free law in hospitality venues in Kampala, Uganda: cross-sectional results from the *KOMPLY* Project. *BMJ open*, *8*(1), e017601. https://doi.org/10.1136/bmjopen-2017-017601.
9. Singh A, Dobbie F, Kinnunen TI, Okello G, Semple S, Okyere PB, Logo DD, Lartey KF, McNeil A, Britton J, Bauld L, Owusu-Dabo E. Adherence to smoke-free policies in Ghana: Findings from a cross-sectional survey of hospitality venue owners and staff. Tob Prev Cessat. 2021;7:4. doi: 10.18332/tpc/131058.

10. Mengesha SD, Shimeles B, Zewdie B, Alermu A, Gerba H, Gartner CE. Smoke-free law compliance and predictive factors in Ethiopia: observational assessment of public places and workplaces. Tob Control. 2023:tc-2022-057750. doi: 10.1136/tc-2022-057750.

11. The Federal Democratic Republic of Ethiopia (FDRE), Federal Negarit Gazette Proclamation No.1112/2019. A Proclamation to Provide for Food and Medicine Administration. 28th February 2019. Addis Ababa, Ethiopia.

12. Argefa TG, Kassa SA, Mdege ND. Second-Hand Tobacco Smoke Exposure and Smoke-Free Environments in Ethiopia: A Scoping Review and Narrative Synthesis. Int. J. Environ. Res. Public Health 2022, 19, 8404. https://doi.org/10.3390/ijerph19148404.

13. Tadesse, T. and B. Zawdie, Non-compliance and associated factors against smoke-free legislation among health care staff in governmental hospitals in Addis Ababa, Ethiopia: An observational cross-sectional study. BMC Public Health, 2019. 19(1):1-11.

14. Singh A, Okello G, Semple S, Dobbie F, Kinnunen TI, Lartey KF, Logo DD, Bauld L, Ankrah ST, McNeill A, Owusu-Dabo E. Exposure to secondhand smoke in hospitality settings in Ghana: Evidence of changes since implementation of smoke-free legislation. Tob Induc Dis. 2020;18:44. doi: 10.18332/tid/120934.

15. Karimi KJ, Ayah R, Olewe T. Adherence to the Tobacco Control Act, 2007: presence of a workplace policy on tobacco use in bars and restaurants in Nairobi, Kenya. BMJ Open. 2016;6(9):e012526. doi: 10.1136/bmjopen-2016-012526.

16. Maskarinec MP, Jenkins RA, Counts RW, Dindal AB. Determination of exposure to environmental tobacco smoke in restaurant and tavern workers in one US city. J Expo Anal Environ Epidemiol. 2000;10(1):36-49. doi: 10.1038/sj.jea.7500069.

17. Jones MR, Wipfli H, Shahrir S, Avila-Tang E, Samet JM, Breysse PN, Navas-Acien A; FAMRI Bar Study Investigators. Secondhand tobacco smoke: an occupational hazard for smoking and non-smoking bar and nightclub employees. Tob Control. 2013;22(5):308-14. doi: 10.1136/tobaccocontrol-2011-050203.

18. Siegel M, Skeer M. Exposure to secondhand smoke and excess lung cancer mortality risk among workers in the "5 B's": bars, bowling alleys, billiard halls, betting establishments, and bingo parlours. Tob Control. 2003;12(3):333-8. doi: 10.1136/tc.12.3.333.

19. International Union Against Tuberculosis and Lung Disease, Campaign for Tobacco-Free Kids, and Johns Hopkins Bloomberg School of Public Health. Assessing Compliance with Smoke-Free Laws: A"How-to" Guide for Conducting Compliance Studies. Second Edition. 2014; 1–36. https:// theunion.org/technical-publications/assessing-compliance-with-smoke-free-laws.

20. Ethiopian Food Medicine and Healthcare Administration and Control Authority (FMHACA). Tobacco Control Directive No. 771/2021. 2021. Addis Ababa, Ethiopia.

21. Petticrew M, Semple S, Hilton S, Creely KS, Eadie D, Ritchie D, Ferrell C, Christopher Y, Hurley F. Covert observation in practice: lessons from the evaluation of the prohibition of smoking in public places in Scotland. BMC Public Health. 2007;7:204. doi: 10.1186/1471-2458-7-204.

22. Goel S, Sharma D, Gupta R, Mahajan V. Compliance with smoke-free legislation and smoking behaviour: observational field study from Punjab, India. Tob Control. 2018;27(4):407-413. doi: 10.1136/tobaccocontrol-2016-053559.

23. Basnet LB, Budhathoki SS, Adhikari B, et al. Compliance with the smoke-free public places legislation in Nepal: A cross-sectional study from Biratnagar Metropolitan City. PLoS One. 2022;17(3):e0264895. doi: 10.1371/journal.pone.0264895.

24. Chowdhury SR, Sunna TC, Das DC, Chowdhury MR, Mahmud HMM, Hossain A. Compliance with smoke-free legislation in public places: An observational study in a northeast city of Bangladesh. PLoS One. 2023;18(4):e0283650. doi: 10.1371/journal.pone.0283650.

25. Barros AJ, Hirakata VN. Alternatives for logistic regression in cross-sectional studies: an empirical comparison of models that directly estimate the prevalence ratio. BMC Med Res Methodol. 2003;3:21. doi: 10.1186/1471-2288-3-21.

26. Singh A, Dobbie F, Kinnunen TI, Okello G, Semple S, Okyere PB, Logo DD, Lartey KF, McNeil A, Britton J, Bauld L, Owusu-Dabo E. Adherence to smoke-free policies in Ghana: Findings from a cross-sectional survey of hospitality venue owners and staff. Tob Prev Cessat. 2021;7:4. doi: 10.18332/tpc/131058.

27. Semple S, Ibrahim AE, Apsley A, Steiner M, Turner S. Using a new, low-cost air quality sensor to quantify second-hand smoke (SHS) levels in homes. Tob Control. 2015; 24(2):153-8. doi: 10.1136/tobaccocontrol-2013-051188.

28. Dobson R, Semple S. "How do you know those particles are from cigarettes?": An algorithm to help differentiate second-hand tobacco smoke from background sources of household fine particulate matter. Environ Res. 2018;166:344-347. doi: 10.1016/j.envres.2018.06.019.

Click here to access/download
Supporting Information
S1 Multistage schematic sampling technique.pdf

Click here to access/download
Supporting Information
S2 Smoke-Free Study Tool.pdf

Click here to access/download
Supporting Information
S3 Operational definitions.pdf
