# Supplementary material for: Compliance with smoke-free laws in hospitality venues in Ethiopia: A cross-sectional observational study in 10 cities
Source: PLoS One. 2025 Feb 21;20(2):e0319079. doi: 10.1371/journal.pone.0319079 (PMC11844872; doi:10.1371/journal.pone.0319079)
Supplement: Attachment — Submitted filename: Response to Reviewers.pdf [file pone.0319079.s006.pdf]

14 October 2024

Point-by-Point Responses

PONE-D-24-30728: Compliance with smoke-free laws in hospitality venues in Ethiopia: a cross-sectional observational study in 10 cities

Reviewers' comments:

Reviewer #1: Investigating the level of implementation to tobacco control law and regulations is a very important task for health policy and public health researchers. This study as the first large scale study looking at hospitality venue smoke-free regulations in Ethiopia, is a valuable contribution to the area. The design, sampling and procedures used in the assessment are up to par with the existing international literature in the area and were reported clearly and concisely in the manuscript. I have made a few comments and requests in the procedures sections of the paper (can be seen in the attached document) to clarify some points but overall, the lit review, design, methods and results sections are well written. However, the discussion section needs a bit more work in terms of concrete policy change and/or implementation process suggestions. To do so, this section also needs to give a bit of background information about the current state of affairs in terms of implementation reinforcement (by whom, how, how frequently are these reinforcements carried out?), especially if there is need for concern of an increase in lack of compliance. The discrepancy reported between this study findings and a previous similar study may indicate such a concern and that needs to be discussed a bit more in detail (suggestions as to how is made in the text attached). Yet, overall, a good sound study, reported successfully.

Thank you.

RESPONSE: *Thank you for your thorough review and valuable feedback on our manuscript. We appreciate your recognition of our study's contribution to the field of tobacco control, as well as your positive assessment of our design, sampling, and methodology. In response to your suggestions, we have carefully revised the manuscript based on your comments. Specifically, we have expanded the discussion section to include detailed background on the current state of enforcement practices in Ethiopia, addressing who is responsible for enforcement, how it is conducted, and its frequency. We also elaborated on potential concerns regarding non-compliance, particularly the discrepancies between our findings and previous studies, and incorporated more concrete policy recommendations for improving tobacco control law implementation. We appreciate your valuable input, which has helped strengthen our manuscript.*

Was there a "walking protocol" in use?

RESPONSE: *Thank you for your question. While we did not specifically refer to a "walking protocol," we utilized a comprehensive field manual to guide all aspects of the fieldwork, including the sampling procedures and data collection activities. This manual ensured*

consistency in the way observers approached and assessed each hospitality venue (HV). It provided detailed instructions on how to conduct observations, what indicators to look for, and the specific areas to assess within each HV (both indoor and outdoor spaces), ensuring a systematic and standardized approach across all study sites.

What was the average length of each observation in each venue (indicate mean and range in minutes please). Did hotel observations take longer since they have a larger size and how did you observe hotel rooms in a covert manner? If hotel rooms were not observed, may be indicating hotel observations as "hotel public areas" would be more appropriate.

RESPONSE: *Thank you for your comment. Data collectors spent approximately 30 minutes at each venue, as outlined in our Standard Operating Procedure (SOP). This observation duration was consistent across venues, regardless of venue size. We have clarified this in the revised manuscript. Regarding the time data, we have two time points: Timestart and Timesubmitted. Timestart indicates when observations began, while Timesubmitted reflects when the data was uploaded, which often occurred the day after completing observations. As such, we cannot precisely determine the exact observation duration from these timestamps. Importantly, hotel rooms were not observed; only public areas like lobbies, corridors, and verandahs were assessed.*

How about cigarette butt litter on the ground around the entrances? Were these part of the observation as well?

RESPONSE: *Thank you and now we have included this variable in the analysis, and also another variable 'tobacco product sales in the indoor'.*

This discrepancy is significant and important to discuss further especially when previously, the overall full compliance rates were reported to be similar in these same two studies. An alarming reason can be that the compliance has dropped over time, which would be a cause for concern and needs to be considered. So we need more info to weigh this unwanted possibility: When authors mention "locational difference" what do they mean? Different cities? If not, different HVs , with such high observational numbers, should not create such a large discrepancy, unless the selection procedures in one or the other study is biased towards observing/not observing. The discrepancy could also be due to seasonal differences (summer vs winter?), can you report dates of data collection for the study mentioned (ref 10)?

RESPONSE: *Thank you and now we have adequately addressed this concern in the revised manuscript that our study only focused on hospitality venues. The previous study "*Mengesha SD, Shimeles B, Zewdie B, Alermu A, Gerba H, Gartner CE. Smoke-free law compliance and predictive factors in Ethiopia: observational assessment of public places and workplaces. Tob Control. 2023:tc-2022-057750. doi: 10.1136/tc-2022-057750*" included government buildings, educational institutions, bars, restaurants and café, food and drinking establishments, healthcare facilities, and hotels. The study was conducted during September to October 2021, a year before our study (Dec 2022). The overall presence of active smoking was 7.7% (7% for bars, restaurants & café, 9.7% for hotels) There is no Most data collection time was done during day, and early evening for HVs.*

Reviewer #2: The paper aims to evaluate the level of compliance with smoke-free laws in hospitality venues (HVs) in Ethiopia. Using the largest sample of venues studied to date, the research investigates the extent to which smoking is absent from both indoor and outdoor HVs in this country. Additionally, it assesses the degree to which these venues adhere to enabling

legal requirements, such as displaying signage and suppressing designated smoking areas.

This paper makes a valuable contribution to the international understanding of smoke-free law compliance. It provides a comprehensive assessment in a diverse, under-researched context, thus filling a critical gap in the global literature about low-income countries. The study's strengths lie in its large-scale investigation across multiple cities, the use of covert observations for data accuracy, and the inclusion of both indoor and outdoor compliance indicators.

RESPONSE: *Thank you for your thoughtful review and for recognizing the value of our study in advancing the global understanding of smoke-free law compliance, particularly in low-income settings like Ethiopia. We appreciate your positive feedback on the strengths of our research, including the large-scale sample, use of covert observations, and comprehensive assessment of both indoor and outdoor compliance indicators. Your comments reinforce the significance of our work, and we are grateful for your support in helping us highlight these important findings.*

Nevertheless, I offer the following comments in the hope of further strengthening the paper. My main concern revolves around the concept of "compliance with smoke-free laws," an issue in this paper and in some scientific literature. For me, compliance should solely indicate the extent to which the law succeeds in preventing smoking where it's prohibited (the law's objective).

While the law might include other requirements, like signage, meant to aid in smoking prevention, high adherence to these doesn't guarantee a decrease in smoking. Many factors influence the success of a smoking ban. Therefore, it's crucial to strictly reserve "compliance with the law" to describe the law's effectiveness in banning smoking.

RESPONSE: *Thank you for your detailed and insightful comments, especially regarding the concept of "compliance with smoke-free laws." We appreciate your perspective on defining compliance strictly as the law's effectiveness in preventing smoking where it is prohibited. We recognize the importance of distinguishing between direct compliance with smoking bans and adherence to supporting requirements, such as signage, which may not directly guarantee smoking reduction.*

We have carefully addressed your concerns in the revised manuscript. Specifically, we clarified the distinction between "compliance with smoke-free laws" and adherence to legal requirements such as signage and DSAs. We have now focused the definition of compliance more strictly on the law's effectiveness in preventing smoking in prohibited areas, while treating adherence to other requirements of the smoke-free law as supporting factors rather than direct indicators of compliance. We appreciate your input in strengthening the clarity and precision of our work and believe the revisions better reflect the law's primary objectives.

In this regard, the paper deviates from a strict definition of compliance in the following ways:
1. Compliance definition: The paper uses a too-broad definition of compliance, encompassing not only the direct observation of smoking (or lack thereof) but also other factors stipulated by the law, such as the presence of 'no smoking' signage, the absence of ashtrays and lighters, and

the absence of designated smoking areas (DSAs). While these factors may undoubtedly contribute to a smoke-free environment, they do not directly measure the core objective of the law, which is to prevent smoking in prohibited areas. As indicated before, one thing is compliance with the aim of the law, and another is adherence to the requirements of the law.

RESPONSE: *Thank you for your detailed and insightful comments, especially regarding the concept of "compliance with smoke-free laws". We appreciate your perspective on defining compliance strictly as the law's effectiveness in preventing smoking in prohibited areas. We recognize the importance of distinguishing between direct compliance with smoking bans and adherence to legal requirements, such as signage, which may not directly guarantee smoking reduction.*

Ethiopia's Tobacco Control Proclamation No. 1112/2019 clearly outlines a comprehensive approach to tobacco control that goes beyond simply preventing smoking in prohibited areas. It mandates both direct measures (e.g., banning smoking in prohibited areas) and enabling measures (e.g., signage, removal of ashtrays and lighters, abandoning DSA and smoking-related aids such as shisha equipment, and no one to use tobacco products within 10m of any air intake mechanism) in prohibited areas to create a smoke-free environment. These enabling factors are crucial to ensuring that the law's core objective—preventing smoking in public and workplace environments—is realized.

However, Ethiopia's comprehensive tobacco control law does not differentiate between active smoking and smoking-enabling factors. Instead, it mandates full compliance with a range of requirements, including the prohibition of active smoking, removal of smoking-related items (such as ashtrays and lighters), the presence of 'no smoking' signage, and the prohibition of DSAs. It is clear that these enabling factors are integral to creating and maintaining a smoke-free environment, and the law treats all these components as equally important to compliance. Therefore, our definition of compliance reflects the comprehensive nature of the law, which treats both the direct prevention of smoking and the removal of enabling factors as equally essential. We hope this clarifies our approach, and we appreciate your input in helping us make this clearer in the manuscript.

In response to your suggestions, we have added the definition of Compliance directly into the last paragraph of the Introduction section of our manuscript. This definition clarifies the context in which compliance is assessed in our study, in addition to its inclusion in the supplementary file. We believe that this change enhances the clarity and comprehensibility of our work.

2. Compliance in Outdoor Spaces: If compliance is meant to solely indicate the law's success in preventing smoking where prohibited, then we need to clarify which outdoor areas are covered by the smoking ban. I understand that the outdoor ban applies only within a 10-meter radius of doorways, windows, or air intakes in public places or workplaces. Thus, observing smoking in outdoor areas of HVs may not violate the law. While the analysis of outdoor smoking is valuable, which I recommend keeping, it shouldn't be considered an assessment of compliance with the core objective of the law.

RESPONSE: *Thank you for your valuable insights regarding compliance in outdoor spaces. We appreciate your request for clarification on the scope of the smoking ban. As you correctly noted, the 2019 Ethiopian Tobacco Control Law explicitly prohibits smoking within a 10-meter radius of doorways, windows, or air intakes of public places and workplaces, and any observed smoking in these areas constitutes a direct violation of the law. While our analysis of outdoor smoking includes observations within this 10-meter radius, we believe it is essential to assess these behaviors to provide a comprehensive view of compliance and public health implications.*

3. Analysis of adherence to enabling requirements of the law: The authors analyze the "factors associated with indoor smoking and below-average compliance." Their analysis is, however, muddled by the lack of clarity about the compliance concept.

a. First, Table 6, which examines the prevalence ratios of "indoor active smoking" and "below-average compliance," encounters a methodological challenge due to the inherent relationship between these two variables. As the text notes, the "below-average compliance" indicator is partially calculated by considering the presence of active smoking indoors. As a result, the 95%CI of PRs overlap for most cities and venue types. I suggest that the "below-average compliance" variable be transformed into a "below-average adherence to enabling requirements" excluding active smoking. By the way, I would also exclude the variable related to the use of tobacco products outdoors within 10 meters from any door, window, or air intake. It conceptually belongs to compliance with outdoor requirements, and it is not explicitly about smoking but the use of any tobacco product, including non-combustible (although I understand non-combustible use is not frequent in the country).

RESPONSE: *Thank you for your insightful comments regarding our analysis of the Average Compliance. We have excluded all analyses based on average compliance, below-average compliance, and below-average adherence from our manuscript, recognizing the lack of clarity surrounding these concepts. We agree that the relationship between "indoor active smoking" and these compliance measures introduces methodological challenges, particularly since the latter is influenced by the presence of active smoking indoors. We appreciate your constructive feedback, which has significantly improved the quality of our manuscript.*

b. The paper only analyzes three variables (city, venue type, and signage) for their association with indoor active smoking. The results indicate that no-smoking signs are linked to the absence of indoor smoking. This is a valuable finding, suggesting to authorities that enforcing this specific requirement in HVs can improve compliance with the law's objective. However, the paper misses an opportunity to explore the relationship between other enabling factors mentioned in the law (absence of ashtrays, lighters, designated smoking areas, and shisha equipment) and actual smoking behavior. Understanding the effectiveness of enforcing these additional factors could provide even more targeted guidance for improving compliance.

I recommend expanding the analysis to estimate the association between all five enabling factors and indoor active smoking, further stratifying the results by venue type. This would provide enforcement authorities with more specific, actionable information about which enabling factors are most likely to prevent indoor smoking in different HV types, facilitating the development of more tailored and effective enforcement strategies.

RESPONSE: *Thank you for your thoughtful and valuable feedback on our analysis. We appreciate your suggestion to expand the exploration of enabling factors in relation to indoor active smoking. We have revised our analysis to include additional variables, such as the presence of ashtrays, lighters, DSA, shisha equipment, tobacco product sales, and outdoor smoking within a 10-meter radius, in the regression model. These variables have yielded important findings that enhance our understanding of factors associated with indoor smoking behavior. Additionally, we included cigarette butts as an outcome variable, which provided further valuable insights. Initially, cigarette butts and tobacco product sales were missed from our analysis, but they are now included in both the manuscript and the results based on an article from the Bulletin of WHO:* doi: http://dx.doi.org/10.2471/BLT.15.158238, *and your suggested two articles:* doi:10.3390/ijerph13121228 and doi: 10.1136/tobaccocontrol-2017-053920.

We believe that these expanded analyses offer a more comprehensive view of the factors influencing indoor smoking and can provide enforcement authorities with more targeted guidance. By understanding the impact of various enabling factors, enforcement efforts can be tailored to specific hospitality venue types to improve compliance with smoke-free laws effectively. We are confident that these additions strengthen the manuscript and align with your recommendation for more actionable insights.

In addition, I offer the following minor comments:

Introduction

• P4 L80: The authors describe the smoke-free requirements of Proclamation No. 1112/2019 as applicable to HVs. L84 specifies that the Proclamation also prohibits using any tobacco product "within 10 meters of any public place or workplace doorway, window, or air intake mechanism." The authors should clarify that the Proclamation prohibits the use of any tobacco product in any outdoor space within that distance.

RESPONSE: *Thank you for your valuable feedback regarding the clarification of smoke-free requirements under Proclamation No. 1112/2019. In response to your suggestion, we have revised the manuscript to explicitly state that the Proclamation prohibits the use of any tobacco product in all outdoor spaces within the 10-meter radius of any public place or workplace doorway, window, or air intake mechanism. This clarification enhances the understanding of the law's scope and its application to HVs in our study.*

Methods

• P5 L111: The authors indicate they selected ten major regional and chartered cities in Ethiopia. To understand the study's results' applicability, it would be informative to know the percentage of the national population living in these areas.

RESPONSE: *Thank you for your comment. We have now added the total population size of Ethiopia (about 120m) and approximately 7 m residing in the 10 selected cities, ranging from over 4 million in Addis Ababa to fewer than 100,000 each in Assosa, Gambella, and Semera-Logia. This population accounts for about 6% of Ethiopia's total population of 120 million in 2022.*

• P6 L139 & S1: In S1, the authors provide a visual aid to explain the selection of sub-cities, woredas, or kebeles. However, L139 states that they "used these lists (of HVs after mapping) to purposively select 2-4 neighborhoods (or villages)," which seem to be the Primary Sampling Units (PSU). I suggest including the number of PSUs (villages) per previous sampling level (sub-cities, woredas, or kebeles) in S1.

RESPONSE: *The sampling structure in S1 was applied uniformly across all study areas. However, the selection of hospitality venues (HVs) in each kebele did not necessarily require the selection of specific villages within those kebeles. We conducted a thorough mapping and listing of HVs in each selected kebele. In Addis Ababa, within the selected woredas, we treated former kebeles as sampling units, as Addis Ababa no longer had a kebele structure during our study. This approach was also applied in Harar city. In cities like Dire Dawa and Gambella, there was no woreda structure, and in Harar, no kebele structure. In most areas, systematic or simple random sampling was used to select the required sample of HVs based on the mapping and listing, whereas in other cases, such as Harar, all identified HVs were included due to their smaller number. The sample distribution to woredas and kebeles was based on these lists.*

We have revised the paragraph to clarify that HVs were selected either through systematic or random sampling based on the comprehensive mapping lists. In areas where the number of HVs was small, all identified HVs were included to ensure full representation.

• P6 L142: The authors state that "to ensure representation of various categories of HVs within the chosen kebele/woreda, the assigned sample size for each kebele/woreda was distributed proportionally across the different types of HVs." This suggests that the study did not collect information on all HVs within each PSU or village but rather on the assigned sample size for each kebele/woreda. Please clarify how the sample size was calculated and its value for each kebele/woreda. Also, explain if the assigned sample was distributed proportionally to the number of HVs per type.

RESPONSE: *The process for selecting the HVs and distributing the sample size across kebeles and woredas was conducted systematically to ensure proportional representation of various types of HVs. As clarified above, in each selected kebele or woreda, we performed detailed mapping and listing of all HVs. The sample size for each kebele or woreda was determined based on the total number of HVs identified in that area, and the assigned sample was distributed proportionally across the different HV types (e.g., hotels, restaurants, cafes) to reflect their actual presence in each kebele or woreda. In areas where the number of HVs was small, all HVs were included.*

• P7 L150: "Data collection tools and procedures": I suggest indicating whether data collectors were instructed to remain for a minimum time at each venue. If not, consider reporting the mean average time of data collection visits in the results section, if you have it. In P22 L396, you discuss that differences between your results and those of other studies "could be due to different observation times and locations". If "observation times" refers to times of day, also consider including the duration of observation visits as a possible reason for differences, as longer visits increase the chance of observing smoking events.

RESPONSE: *Thank you for your comment. The data collectors spent approximately 30 minutes at each venue, and we have included this information in the revised manuscript. We have only two time points:* ***Timestart*** *and* ***Timesubmitted***. ***Timesubmitted*** *refers to when the data collectors uploaded their observations to the server, which does not necessarily coincide with when they finished their observation*

at the venue. In most cases, data submission occurred the next day after ensuring that the data was ready, though there were instances where the submission time matched the end of the observation. Therefore, we cannot determine the exact duration of time spent at each venue. However, our SOP required that the data collectors remain approximately 30 minutes at each venue, observing both indoors and outdoors.

• P8 L174 Smoke-free compliance indicators: Redraft this section according to the stricter concept of compliance discussed above. Compliance will be measured only through the observance or not of smoking, where prohibited by law. Observing signage, ashtrays, lighters, designated smoking areas, and shisha equipment would measure adherence to other law requirements.

RESPONSE: *Thank you for your insightful feedback regarding the smoke-free compliance indicators. In response, we have revised the section to align with the stricter definition of compliance as you suggested. The updated indicators now clearly differentiate between true compliance—measured solely by the observation of active smoking where it is prohibited—and adherence to other requirements of the law, including the newly added indicator for cigarette butts.*

Smoke-free compliance indicators:
Indoor space compliance
The following smoke-free law indicator assessed true compliance indicator:

1. *Observation of active smoking: No one was seen smoking a tobacco product in the venue's indoor space (0 = no, 1 = yes).*

The following indicators assessed indoor adherence to the law's requirements and enabling factors:

2. *Ashtrays: No ashtrays or other instruments used to hold cigarette ash were present inside (0 = no, 1 = yes).*
3. *Lighters: No lighter was visible inside (0 = no, 1 = yes).*
4. *Signage: A display of 'no smoking' signage was observed (0 = no, 1 = yes).*
5. *DSA: No DSA present (0 = no, 1 = yes).*
6. *Shisha equipment: No shisha equipment was seen (0 = no, 1 = yes).*
7. *Cigarette butts: No cigarette butts were observed indoors (0 = no, 1 = yes).*
8. *Tobacco product sale: No tobacco products sale in indoors (0 = no, 1 = yes)*
9. *Outdoor smoking within 10m: No one observed smoking within 10m of any air intake (0 = no, 1 = yes)*

Outdoor space compliance
The following smoke-free law indicator assessed true compliance indicator:

1. *Observation of smoking: No one was seen smoking a tobacco product in the venue's outdoor space (0 = no, 1 = yes).*

The following indicators assessed outdoor adherence to the law's requirements and enabling factors:

2. *Ashtrays: No ashtrays or other instruments used to hold cigarette ash were present (0 = no, 1 = yes).*
3. *Signage: A display of 'no smoking' signage was observed (0 = no, 1 = yes).*
4. *Cigarette butts: No cigarette butts were observed outdoors (0 = no, 1 = yes).*
5. *DSA: Absence of DSA in the outdoor space (0 = no, 1 = yes).*

• P8 L180. I am also unclear about why you want to know for indoor compliance if "nobody used tobacco products in the outdoor place within 10 meters from any door, window, or air intake mechanism." Firstly, tobacco products can include non-combusted products (that don't

emit smoke). Secondly, this appears to be the only HV outdoor space where smoking is prohibited by law.

RESPONSE: *Thank you for your valuable feedback regarding our inclusion of the variable related to smoking within 10 meters of doors, windows, or air intake mechanisms as part of our indoor compliance assessment. While this smoking behavior occurs outdoors, we argue that its inclusion is conceptually justified due to the significant risk of secondhand smoke (SHS) exposure for individuals inside the venue. It is known that smoke can enter indoor spaces through openings, and thus monitoring smoking behavior in this area is essential for assessing potential risks to patrons and staff at hospitality setting. Our definition of indoor compliance extends beyond just monitoring smoking within enclosed indoor spaces; it aims to evaluate the overall effectiveness of smoke-free legislation in protecting indoor air quality.*

By tracking smoking behavior near entry points such as entrances and windows of the HVs, we provide a more comprehensive understanding of how well these laws are enforced and the extent to which they mitigate SHS exposure. Ultimately, the goal of smoke-free legislation is to safeguard public health by reducing exposure to SHS in all forms, and including this variable aligns our analysis with this objective. We believe that retaining this variable will strengthen our study and provide a clearer picture of indoor compliance and SHS exposure risks. In this study, indicator 'no one was seen smoking in the outdoor area within 10 meters of any door, window, or air intake mechanism' was used as one of the enabling factors for the indoor compliance. Our focus was on tobacco smoking and not all tobacco products that can include non-combusted products. We have corrected the definition of out 'outdoor smoking within 10 meters' accordingly throughout the manuscript.

• P8 L183. If I am correct about the prohibition of smoking in the outdoor place within 10 meters from any door, window, or air intake mechanism being the only outdoor no-smoking requirement applicable to HVs, then compliance with the law should only refer to smoking in these areas and the enabling factor requirements only applicable to these limited 10 meters outdoor areas. If the observance of active smoking can be placed within these 10 meter areas, then I would call it compliance. Otherwise, I would not call the indicators about the observation of smoking outdoors and their facilitators "compliance indicators" since the law requires none of the four indicators you propose. I would call them "exposure to SHS outdoors and related indicators."

RESPONSE: *Thank you for your continued feedback on this matter. We have addressed these concerns in detail in our previous response regarding the inclusion of 'outdoor smoking' within 10m in our analysis. We appreciate your understanding as we aim to clarify our focus on comprehensive understanding of indoor compliance.*

• P9 L197. Operational definitions: You define compliance as "the degree to which the HVs fully implement Proclamation No.1112/2019. However, such Proclamation does not seem to ban smoking in all outdoor spaces of HVs. Hence, the clarification I suggest above is whether you are measuring adherence to the law requirements or the law's direct impact (true compliance) on smoking in indoor and outdoor spaces of HVs.

RESPONSE: *Thank you for your valuable insights regarding the operational definitions. We have revised the definitions accordingly.*

Also, the law's and the S3 definition of outdoor spaces in hospitality venues is ambiguous, potentially impacting the study's interpretation. The study's limitations section should acknowledge this ambiguity, highlighting how hybrid spaces and temporary structures complicate the categorization of indoor versus outdoor areas. This lack of clarity could affect the accurate compliance assessment with outdoor smoking regulations, potentially influencing the study's findings.

RESPONSE: *Thank you. We have reflected the definition of outdoor spaces and highlighted its effect on assessing compliance in the Limitation section.*

• P9 L206. Data processing and analysis. I find the computation of the overall "average compliance" with the smoke-free laws across all HVs problematic in several ways. First, it includes true compliance and adherence to enabling factors, which should be measured separately. Second, it is not related to specific venues, assuming that adherence is an average. Second, it can mask the underlying non-adherence to key requirements. Suppose your analysis determines that no smoking signage is the most predictive enabler of no smoking in venues. If most venues adhere to every other requirement but not the signage, you may have a high overall average adherence to the law despite a low adherence to the key signage requirement. I suggest dropping overall average adherence by type of HV and city.

RESPONSE: *Thank you for your valuable feedback regarding the computation of the overall "Average Compliance" with the smoke-free laws across all HVs. We appreciate your insights and have carefully considered your concerns. In response, we have decided to drop the "Overall Average Compliance" analysis from our manuscript, as we recognize the issues you raised. Specifically, we agree that combining true compliance and adherence to enabling factors may obscure critical differences between these measurements and mask underlying non-compliance to key requirements. We also acknowledge that treating adherence as an average across different venue types and cities can dilute our understanding of specific compliance issues, particularly when certain essential factors, such as the presence of 'no smoking signage', are not consistently adhered to. Your suggestions have significantly improved the clarity and focus of our analysis, and we are grateful for your guidance.*

• P9 L211. Data processing and analysis. The overall "indoor compliance" metric mixes true compliance and adherence to enabling factors. I suggest splitting the compliance indicator into a true compliance indicator where the primary outcome analyzed is active smoking and adherence to enabling requirements, whose enforcement may best predict the level of smoking indoors and outdoors. This may help the enforcement authority focus on the enforcement efforts to meet the most predictive requirements of success (no smoking).

RESPONSE: *Thank you for your insightful comments regarding the classification of HVs based on overall compliance with smoke-free laws. We acknowledge the importance of distinguishing between true compliance, indicated by active smoking, and adherence to enabling factors, which can inform enforcement efforts. However, we believe that our current classification system effectively highlights the overall status of HVs by providing a comprehensive view of both compliance and adherence. By categorizing HVs as 'fully compliant,' 'good compliance,' 'moderately compliant,' and 'poorly compliant,' we present a good understanding of the overall smoke-free environment.*

Results
Tables. To facilitate reading and comparing table values, arrange the city names and venue types in the same order in all tables.

RESPONSE: *Thank you. We have corrected and arranged the city names and venue types across all the Tables consistently.*

Redraft the results, including two outcome variables (compliance and adherence to legal enabling requirements), and determine which of the enabling factors best predicts compliance.

RESPONSE: *Thank you for your valuable feedback regarding the need to clarify our results by distinguishing between compliance and adherence to legal enabling requirements. We have taken your suggestion into account and have revised the manuscript accordingly. In the updated results section, we now clearly present two distinct outcome variables: true compliance, indicated by active smoking, and adherence factors. Additionally, we have conducted further analyses to determine which enabling factors best predict active smoking and presence of cigarette butts. These changes enhance the clarity of our findings and provide more actionable insights for enforcement authorities. We appreciate your guidance in strengthening our manuscript.*

Discussion.
P 21 L371.- I suggest restructuring the discussion of the main findings according to the suggested redrafted results.

RESPONSE: *Thank you for your suggestion regarding the restructuring of the discussion section. In the revised manuscript, we have restructured the discussion of our main findings to align with the newly redrafted results. This restructuring enhances the clarity and coherence of our arguments, allowing for a more logical flow between the results and their implications. We appreciate your feedback, which has been instrumental in improving the overall quality of our discussion.*

I suggest expanding the discussion to consider the literature on facilitators and barriers to smokers' compliance with smoking bans. See for example, doi:10.3390/ijerph13121228 and doi: 10.1136/tobaccocontrol-2017-053920

RESPONSE: *Thank you for your valuable suggestion to expand the discussion on the facilitators and barriers to smokers' compliance with smoking bans. We have incorporated relevant literature, including the studies you suggested, to provide a more comprehensive understanding of these factors in our revised manuscript.*

Conclusions
P23 L434. I disagree with the author's assessment of good compliance with smoke-free laws in Ethiopia, particularly considering the low prevalence of tobacco smoking in the country. Non-compliance with smoke-free laws should be considered a violation of fundamental human rights and a breach of the WHO Framework Convention on Tobacco Control. In light of this, any instance where more than 5% of venues exhibit smoking in prohibited areas should be considered problematic compliance. Similarly, non-adherence to key enabling factors in more than 5% of venues is equally concerning.

RESPONSE: *We appreciate your valuable feedback regarding our conclusions on compliance with smoke-free laws in Ethiopia. We acknowledge your concerns about the implications of non-compliance, and we have revised our conclusions to emphasize that any instance of smoking in prohibited areas or non-adherence to enabling factors is indeed problematic and should be addressed seriously in the context of public health and human rights.*
