# Supplementary material for: Compliance with smoke-free laws in hospitality venues in Ethiopia: A cross-sectional observational study in 10 cities
Source: PLoS One. 2025 Feb 21;20(2):e0319079. doi: 10.1371/journal.pone.0319079 (PMC11844872; doi:10.1371/journal.pone.0319079)
Supplement: Attachment — Submitted filename: Point-by_Point_Responses_R1_Revised.pdf [file pone.0319079.s009.pdf]

08 January 2025

<u>Point-by-Point Responses</u>

PONE-D-24-30728R1: Compliance with smoke-free laws in hospitality venues in Ethiopia: a cross-sectional observational study in 10 cities

Reviewers' comments:

Reviewer #2:

LINE	ORIGINAL IN THE DOCUMENT WITH TRACK CHANGES	COMMENTS
96-97	However, studies in sub-Saharan Africa have indicated a lack of knowledge and low levels of compliance with smoke-free laws in HVs [8-10].	It is not clear what the “lack of knowledge” refers to. Lack of knowledge of the health effects of SHS? Lack of knowledge of compliance? If this second situation is what it refers to, then it does make sense that studies don´t know what compliance is AND at the same time, they know that compliance is low. Please clarify.
	Author’s Responses: Thank you for your insightful comments. We have clarified the statement to indicate that the lack of knowledge refers to the requirements of smoke-free laws, leading to low compliance levels among hospitality venue staff in sub-Saharan Africa. We appreciate your thorough review and constructive feedback.	
99	Studies indicate a high prevalence of indoor smoking in HVs [8, 11, 12],	Studies where? In Africa? Worldwide? Please clarify
	Author’s Responses: Thank you for your valuable comments and suggestions. To clarify, the studies referenced indicate a high prevalence of indoor smoking in hospitality venues specifically in sub-Saharan Africa, including Uganda, Ghana, and Kenya. We have updated our statement to reflect this geographical context and created a separate paragraph for clarity.	
122-123	One of the two studies only studied five government hospitals in Addis Ababa [20]	Avoid repetition of the word study for better style. Perhaps write: “One of the two studies was restricted to five government hospitals in Addis Ababa.”
	Author’s Responses: Thank you for your helpful comment. We have revised the sentence to avoid the repetition of the word "study" as you suggested. We appreciate your constructive feedback and the opportunity to improve the style and clarity of our manuscript.	
142-143	In this study, we assessed the implementation of smoke-free laws, focusing on two related concepts: true compliance and adherence to the law’s requirements.	The two related concepts are compliance and adherence to the law. There is no true compliance as opposed to false compliance. I suggest writing “… focusing on two related concepts: compliance with the smoking ban and adherence to its other requirements.” Please do not use “true” to refer to compliance.
	Author’s Responses: Thank you for your valuable comments and suggestions. We have revised the sentence to avoid the term "true compliance" and to reflect the concepts more accurately. The updated sentence now reads: "In this study, we assessed the implementation of smoke-free laws, focusing on two related concepts: compliance with the smoking ban and adherence to the law’s requirements. Compliance refers to the law’s effectiveness in preventing active smoking in prohibited areas and serves as a key indicator of its success." We appreciate your constructive feedback and the opportunity to improve the clarity and precision of our manuscript.	

195	We then used the comprehensive mapped lists to select HVs, either through systematic or random sampling.	Please clarify when you used systematic sampling and when you used random sampling.
	Author's Responses: Thank you for your insightful comments. We have carefully reviewed your comments and provided additional details to clarify our sampling methods and criteria. Specifically, systematic sampling was used in cities such as Hawassa and Adama, while random sampling was applied in Addis Ababa and Bahir Dar. We appreciate your thorough review and the opportunity to enhance the clarity of our manuscript.	
196	In areas where the total number of HVs was small,…	You may want to clarify your criteria for a small n. For example, you could write, "In areas where the total number of HVs was small (n<x),…"
	Author's Responses: In smaller cities like Harar, where the total number of HVs was particularly low, we included all identified HVs in the study. The selection method was based on the local context, and all identified HVs were included when the mapped HVs were less than or equal to the allocated sample size. Therefore, there is no specific 'n' threshold that can be stated. We have not made changes to the original paragraph but have provided this clarification to address your comments.	
227	Data collectors observed compliance with the presence of "no smoking" signage at the main entrance and inside the venue,…	The use of "compliance" is not appropriate. I suggest rephrasing as "Data collectors observed if "no smoking" signage was properly displayed at the main entrance and inside the venue,…".
	Author's Responses: Thank you for your valuable comment. We have accommodated your suggestion and revised the sentence accordingly.	
229 & 231	They also noted if anyone was smoking within 10 meters of any main door, window, or air intake mechanism. They also asked the waiter/waitress if …..	For style reasons, avoid repeating "they also" in L231. Perhaps use "In addition they…"
	Author's Responses: Thank you for your valuable comment. We have accommodated your suggestion and revised the sentence accordingly.	
245	9) no one was seen smoking tobacco products in the outdoor area within 10- meters from any door, window, or air intake mechanism (0=no, 1=yes)	Remind the reader that indicator nine was obtained even if the venue did not have an outdoor area to serve the public.
	Author's Responses: Thank you for your valuable comment. We have clarified that indicator nine was obtained for all HVs, regardless of whether the venue had an outdoor area to serve the public.	
247	The first indicator measures the true compliance with smoke-free law, …	As indicated before, I suggest dropping the term true to refer to compliance. I suggest rewriting. "The first indicator measures the compliance with smoke-free law,…"
	Author's Responses: Thank you for your valuable comment. We have removed the 'true' from the statement.	
250	Outdoor space compliance: The following five smoke-free specific indicators were used to assess the outdoor space compliance and adherence:	Conceptually, these indicators can't measure compliance or adherence to the law because the law does not ban smoking outdoors. I suggest rewriting: "Outdoor Smoking: We also conducted an assessment of smoking-related activity in all outdoor spaces where smoking is permitted. The following five indicators were used: …"
	Author's Responses: Thank you for your valuable comment. We have clarified that outdoor space refers to any area outside of any HV that is not enclosed but serves the public, where smoking is prohibited. Smoking is not permitted	

<table>
<tr><td></td><td colspan="2">in these outdoor public spaces. The updated sentence now reads: "The following five smoke-free specific indicators were used to assess outdoor space compliance and adherence, defined as any area outside of any HV that is not enclosed but serves the public, where smoking is prohibited: 1).....". A total of 329 HVs included in the sample had such outdoor spaces in addition to indoor spaces.</td></tr>
<tr><td>332
344</td><td>Section on Indoor active smoking and enabling factors
Section on Compliance and adherence with smoke- free laws in indoor areas</td><td>These two sections contain the same information. The first presents it as noncompliance and nonadherence, and the second as compliance and adherence. I suggest keeping only one, perhaps as “Compliance with the law and adherence to the law requirements in indoor areas,” since Table 2 presents compliance and adherence data.</td></tr>
<tr><td></td><td colspan="2">Author’s Responses: Thank you for your valuable comment. We appreciate your feedback regarding the sections on indoor active smoking and enabling factors, and compliance and adherence with smoke-free laws in indoor areas. We would like to clarify the distinction and similarities between these two sections.

In the first section, titled "Indoor active smoking and enabling factors," we aim to provide a detailed textual description of the prevalence of indoor active smoking and the factors that enable it. This section includes data with 95% CIs to highlight the extent of the issue without using tables. This narrative description is essential for understanding the context and underlying reasons for noncompliance. The second section, titled "Compliance and adherence with smoke-free laws in indoor areas," focuses on presenting compliance and adherence data using Table 2. This section provides an assessment of how well the laws are being followed, based on specific indicators.

By maintaining both sections, we ensure a comprehensive understanding of the issue. The first section sets the stage by describing the prevalence and enabling factors of indoor active smoking, while the second section provides a clear and structured presentation of compliance and adherence data. These sections complement each other, offering both a narrative and a data using the Table, which is critical for a thorough analysis. Therefore, we believe that keeping both sections is important for providing a complete picture of the situation. We hope this explanation justifies our decision to retain both sections in the manuscript.</td></tr>
<tr><td>353</td><td>Results</td><td>Make it more straightforward when you talk about compliance and when you do about adherence.</td></tr>
<tr><td></td><td colspan="2">Author’s Responses: Thank you for your valuable feedback on our RESULT section. We understand the importance of clearly distinguishing between compliance and adherence in our manuscript. We have made every effort to address your comment by ensuring that each instance of compliance and adherence is presented in a straightforward and distinct manner. Specifically, we have carefully revised the text to avoid any overlap or ambiguity between the two concepts, and have clearly defined each term in the context of our study. Compliance is discussed in terms of how well the HVs follow the smoke-free laws, such as the absence of smoking activity in prohibited areas. Adherence is addressed in terms of the venues' alignment with the broader requirements of the smoke-free laws, such as displaying ‘no smoking’ signage, preventing the presence of smoking-related paraphernalia to maintain SFE.</td></tr>
<tr><td>363</td><td>Table 2. Compliance and adherence with smoke-free laws indicators in indoor areas by cities and HVs</td><td>I suggest tweaking the title of Table 2 and saying, “Compliance with the law and adherence to the law requirements in indoor areas by city and HV type.”</td></tr>
<tr><td></td><td colspan="2">Author’s Responses: Thank you for your valuable feedback and we have accommodated it.</td></tr>
<tr><td>283 &
377</td><td>Data processing and analysis
Table 3. Indoor compliance level with smoke-free laws by cities and HVs</td><td>Now that you have adopted the concepts of compliance with the law and adherence to its requirements, the classification of HVs into levels of compliance and Table 3 does not make much sense. Compliance should be measured ONLY by seeing active smoking. Then, you could classify levels of adherence to the requirements of</td></tr>
</table>

		the law as 'fully adherent,' 'good adherence,' 'moderately adherent,' and 'poorly adherent. '<br><br>I suggest rewriting the piece in the section on data processing and analysis as follows:<br>We classified each city and HV type as compliant with the law when no active smoking was detected and as noncompliant when it was detected. In addition, Indoor venues were classified as 'highly adherent,' 'moderately adherent, and 'poorly adherent' with the legal smoke-free requirements when they met 7-8, 5-6, and > 5 of these requirements, respectively.<br>Accordingly, I also suggest rewriting Table 3 with a new appropriate title and only three levels of adherence. Also, rewrite lines 374-376 to describe results and adherence from Table 3.
	Author's Responses: Thank you for your valuable comments and suggestions. We have revised our manuscript to separately analyze Compliance and Adherence to Smoke-Free law requirements for indoors and outdoors. Specifically, we have made the following changes:<br>1. *Updated the Data Analysis section to clarify the distinction between compliance and adherence.*<br>2. *Revised Table 3 and its accompanying text to reflect the new classification of adherence levels.*<br>3. *Revised Table 5 and its accompanying text to align with the new approach.*<br>*We appreciate your insights, which have greatly improved the clarity and quality of our manuscript. However, we still maintained the classification of Indoor Adherence level by four: Fully compliant (8 points), highly adherent (7 points), moderately adherent (5-6 points) and poorly adherent (<5 points), The frequencies in each category are larger and we wanted to keep the original classification.*	
287 & 417		As indicated before, given that the law doesn't ban smoking in outdoor areas, it doesn't make sense to talk about compliance with or adherence to the law in these areas. For this reason, II would suppress lines 287-291 in methods and lines 417-418 along with Table 5 in results.
	Author's Responses: Thank you for your valuable comment. We have clarified above that outdoor space refers to any area outside of any HV that is not enclosed but serves the public, where smoking is prohibited. Smoking is not permitted in these outdoor public spaces. Therefore, we would like to maintain our original descriptions in the manuscript.	
383 & 394	Section on Outdoor active smoking and enabling factors<br>Section on Compliance and adherence with smoke-free laws in outdoor spaces	These two sections contain almost the same information. The first presents it as negative behavior (smoking) and "no smoking" signage, while the second is as positive behavior (non-smoking) and absence of ashtrays. I suggest keeping Table 4 and retitling it as "Smoke-free indicators in outdoor areas by city and HV type" and combining both sections to describe no-smoking behavior, as well as the other four indicators.<br><br>*Author's Responses: Thank you for your valuable comment. We appreciate your feedback regarding the sections on outdoor smoking behavior and smoke-free indicators in outdoor areas. We would like to clarify the distinction and similarities between these two sections.*<br><br>*In the first section, titled "Outdoor smoking behavior and enabling factors," we aim to provide a detailed textual description of the prevalence of outdoor smoking and the factors that enable it. This section includes data with 95% CIs to highlight the extent of the issue without using a Table. This narrative*

		description is essential for understanding the context and underlying reasons for noncompliance.<br><br>*The second section, titled "Smoke-free indicators in outdoor areas by city and HV type," focuses on presenting compliance and adherence data using Table 4. This section provides an assessment of how well the laws are being followed, based on specific indicators such as the absence of ashtrays and the presence of "NO SMOKING" signage.*<br><br>*By maintaining both sections, we ensure a comprehensive understanding of the issue. The first section sets the stage by describing the prevalence and enabling factors of outdoor smoking behavior, while the second section provides a clear and structured presentation of compliance and adherence data. These sections complement each other, offering both a narrative and a data, which is critical for a thorough analysis.*<br><br>*Therefore, we believe that keeping both sections is important for providing a complete picture of the situation. We hope this explanation justifies our decision to retain both sections in the manuscript.*
		By the way, the results for the NoDSA indicator are not described in the results section text. Should the NoDSA requirement for outdoor areas be analyzed at all? While a NoDSA requirement for indoor spaces is clearly positive, its value for outdoor areas where smoking is permitted is more controversial. On the one hand, it may transmit that non-smokers need to be protected in DSA outdoors (which is positive for public health). At the same time, because they are ineffective in protecting non-smokers from SHS, they may be considered an excuse not to ban smoking outdoors (which is negative for public health). For these reasons, I wonder if the NoDSA indicator should not be analyzed at all and should be dropped.
		Author's Responses: Thank you for your insightful comments. We have already included the results for the NoDSA indicator in the results section text in the first statement to provide a comprehensive view of adherence to the smoke-free law in outdoor spaces.<br><br>*We would like to clarify that the NoDSA variable is equally important for our analysis as it is clearly stated in the smoke-free law for Ethiopia. The law mandates the absence of DSAs in both indoor and outdoor public spaces to protect non-smokers from exposure to secondhand smoke. Therefore, it is essential to include and analyze the NoDSA requirement for outdoor areas, as compliance with this aspect of the law is critical for ensuring a SFE.*<br><br>*Thank you for your more elaboration about the DSA issues in general.*
427	Section on Factors associated with active smoking and cigarette butts in indoor places	The section analyzes the factors (law requirements) associated with active smoking and cigarette butts separately. The authors do not justify why in addition to active smoking, they also investigate the association with the finding of butts. Please justify it.<br>Also, I wonder why they don't investigate the association between active smoking and finding butts.

	Author's Responses: Cigarette butts are a practical proxy for assessing active smoking in smoke-free environment studies, as they provide evidence of recent smoking activity that direct observation might miss. However, their presence is not used as a predictor of active smoking, as both measures are interrelated and could exhibit multicollinearity, complicating statistical analyses. Despite this, cigarette butt counts remain a valuable, cost-effective tool for evaluating non-compliance with smoke-free policies, identifying smoking hotspots, and complementing other methods like air quality monitoring. Their accuracy can still be influenced by environmental factors such as cleaning and the persistence of butts over time. In addition, investigating cigarette butts separately from active smoking allows us to capture different dimensions of non-compliance. Active smoking provides a snapshot of current behavior, whereas cigarette butts offer a retrospective record of smoking activity, which can be critical for identifying persistent problem areas and understanding patterns over time. This dual approach enables a more comprehensive assessment of adherence to smoke-free laws.<br><br>*The reason we have not investigated the association between active smoking and finding butts is due to the potential for multicollinearity, as both variables are indicators of smoking activity and may not provide additional independent information. Instead, we focus on separately analyzing these factors to avoid statistical complications and to provide clear, distinct insights into each aspect of non-compliance.*	
441-442	… before and after adjustment.	Please describe in the text or Table 6 the variables for which you adjusted.
	Author's Responses: Thank you for your valuable comment. We appreciate your detail attention. We would like to clarify that all the adjusted variables are already presented in Table 6 (City, HV type, the 7 adherence factors). Therefore, we believe it is unnecessary to describe them again in the text. We hope this explanation justifies our decision to retain the current format.	
454	Table 6.	The last six rows of Table 6 reflect factors that represent law violations. The row on smoking signs should also be phrased and analyzed as a violation of the law (absence of signs) so that the PRs can show the same direction as the other violators and make them more comparable.
	Author's Responses: Thank you for your valuable comment. We appreciate your insight regarding the presentation of the "No Smoking Signage" variable in Table 6. We would like to clarify that most of the literature assessing the relationship between No Smoking Signage and Active Smoking examines the effect of the presence of No Smoking Signage on active smoking. This is typically presented as a positive indicator rather than a violation.<br><br>*Presenting No Smoking Signage in this manner allows us to better illustrate how its presence reduces or affects active smoking. Changing the phrasing to reflect it as a violation (absence of signs) may obscure this important relationship. Therefore, we believe it is more informative to maintain the current presentation to align with existing literature and to clearly communicate the impact of No Smoking Signage on reducing the prevalence of active smoking in HVs.*	
474	The higher prevalence of outdoor smoking in our study suggests that smokers may be shifting to outdoor areas in response to indoor smoking bans.	In my view, the data does not necessarily suggest the migration of smokers. I suggest rephrasing it as a hypothesis to be studied. The data would suggest a migration of smokers only if the number of venues with outdoor smoking had increased after the entry into force of the indoor ban. Otherwise, the prevalence of smoking might have been high before the indoor ban. Since the study does not present the before and after the ban data, "suggests" is too strong a word.
	Author's Responses: Thank you for your valuable comment. We acknowledge that our data does not necessarily confirm the migration of smokers to outdoor areas, as it does not include the before-and-after comparisons of the indoor ban. Therefore, we have rephrased the statement as a hypothesis to be studied. The revised statements now read: "The higher prevalence of outdoor smoking in our study raises the hypothesis that smokers may be shifting to outdoor areas in response to indoor smoking bans. Furthermore, it is plausible that HV managers may encourage outdoor smoking if indoor smoking makes customers uncomfortable". We appreciate your feedback and the opportunity to enhance the accuracy and clarity of our manuscript.	

366<br>390	While adherences were notably low for cigarette butts<br>However, these findings must be viewed alongside lower compliance rates for key indicators like active smoking and cigarette butts,	In L366, the presence of cigarette butts is presented as an adherence factor, while in L391, it is presented as a key indicator for compliance. Please, choose one term to depict the presence of cigarette butts and use it consistently.
	Author's Responses: Thank you. We have removed the 'cigarette butts' in L391 to be consistent throughout the manuscript.	
485-488	Interestingly, our study found exceptionally high adherence rates regarding the absence of DSAs and shisha equipment, indicating that smoke-free legislation is effective in specific contexts where enforcement and public health messaging are prioritized,	I would use a more tentative language since you do not have a before-after design to indicate if SF legislation has reduced DSAs and shisha equipment. "suggesting that smoke-free legislation might be effective"
	Author's Responses: Thank you for your valuable comment. We have made the modification based on your comment.	
506	The presence of 'no smoking' signage was significantly associated with reduced active smoking rates and the prevalence of cigarette butts.	This statement is factually incorrect. First, the association is not with smoking rates but the prevalence of venues with active smoking in violation of the ban. Second, the aPR for active smoking is 1.3 (presented as 0.77) and not significant for the presence of butts. An aPR of 1.3 (95% CI 1.1-1.5) is not that impactful, particularly compared to the other violation factors. Given that the following sentence is offered as a contrast, this sentence seems involuntarily erroneous. Please correct.
	Author's Responses: Thank you for your valuable comment. We apologize for the oversight in our statement. We have corrected it to accurately reflect the data. The revised statement now reads: "The presence of 'no smoking' signage was significantly associated with reduced prevalence of venues with active smoking in violation of the ban'. We appreciate your feedback and the opportunity to improve the accuracy of our manuscript.	
515	Our study showed a significant association between such signage and decreased active smoking rates and cigarette butt prevalence.	The same error as above is repeated here.
	Author's Responses: Thank you for your valuable comment. We apologize for the repetition of the previous error in our statement. We have corrected it to accurately reflect the data.	
518	These results highlight the necessity for … enhanced visual enforcement, such as prominent 'no smoking' signage.	Given the above two comments, this conclusion does not seem warranted.
	Author's Responses: Thank you for your valuable comment. We acknowledge that the aPR for venues with active smoking, associated with the presence of 'no smoking' signage is 1.3 (95% CI 1.1-1.5), indicating a moderate impact. Based on this finding, while the effect may not be highly impactful compared to other violation factors, we still see value in context-specific enforcement strategies and enhanced visual enforcement. Therefore, we have revised our statement to better reflect this context.	
528	In our study, full compliance with smoke-free laws was observed….	To be coherent with the distinction between compliance and adherence, the sentence should be written as "In our study, compliance with smoke-free law along with full adherence to its requirements was observed…"
	Author's Responses: Thank you for your valuable comment. We have revised the statement to be coherent with the distinction between compliance and adherence.	

555-565	The findings of this study have significant policy implications for enhancing tobacco control efforts in Ethiopia,… Increasing the visibility and enforcement of ‘no smoking’ signage can serve as an effective policy measure….	In my view, the small association found in the study between the absence of “no smoking” signage and indoor active smoking does not justify the strong emphasis on enforcing such signage. According to Table 6, the three factors more strongly associated with the detection of active smoking in indoor places where smoking is banned are smoking within 10 m, the presence of lighters, and sales of tobacco products. Given that non-compliance with the “*no smoking within 10 m*” requirement of the law is high, around 50%, focusing on enforcement of this requirement seems advisable when designing implementation efforts, either through enforcement authorities or public education. Perhaps this advice should be clearly discussed.<br>If the authors still want to recommend the enforcement of signage, they could say that despite the results of this study, the international experience shows the importance of such signage.
	Author’s Responses: Thank you for your valuable comment. We appreciate your feedback regarding our emphasis on 'no smoking' signage. While our study found a moderate association between the presence of 'no smoking' signage and the reduced prevalence of active smoking in venues, we acknowledge that the aPR for active smoking is 1.3 (95% CI 1.1-1.5). Given this, we agree that focusing on the enforcement of the “no smoking within 10 m” requirement is crucial, especially since non-compliance with this aspect of the law is around 50%. Additionally, addressing the presence of lighters and the sales of tobacco products is essential for a comprehensive enforcement strategy.<br><br>*However, we still recommend the enforcement of 'no smoking' signage, as international experience demonstrates its importance. Clear signage and local authority involvement have been shown to significantly boost compliance rates in other countries. Therefore, we believe that a combination of context-specific enforcement strategies, including enhanced visual enforcement with prominent ‘no smoking’ signage, is essential for effective tobacco control. We have modified our paragraph to reflect these points and provide a balanced perspective.*	
577-585	Conclusions. … alongside enhanced enforcement of 'no smoking’ signage…	Same comment as above.
	Author’s Responses: Thank you for your valuable feedback. We have made revisions in our Conclusion to address your comments and expanded it based on our finding to ensure clarity.	

Reviewer #3:

Line	Original in the Cleaned Manuscript	Comment
3-4	Title: Compliance with smoke-free laws in hospitality venues in Ethiopia: a cross-sectional observational study in 10 cities	• Topic/title can be rewritten as **Compliance with smoke-free laws in hospitality venues and factors associated with non-compliance in.......**<br>• Your title and your ethical clearance letter are not exactly the same. Any modification?
	Author’s Responses:<br>*Topic/Title: Thank you for your valuable feedback on the manuscript title. We appreciate your suggestion to include factors associated with non-compliance in the title. While we acknowledge the importance of these factors, the primary focus of our study was on compliance with smoke-free laws in hospitality venues. Additionally, the suggested title may be too long for an article title.*	

	Therefore, we would prefer to retain the original title: "Compliance with smoke-free laws in hospitality venues in Ethiopia: a cross-sectional observational study in 10 cities." We believe this title accurately reflects the main objective of the study while keeping it concise and focused. Thank you again for your valuable feedback.<br><br>*Ethical Clearance Topic: Thank you for your valuable comment regarding the title discrepancy. We appreciate the opportunity to clarify this. The title approved by the IRB, "Compliance with Smoke-free Laws and Tobacco Advertisement, Promotion and Sponsorship Bans in Ethiopia," is an overarching topic that encompasses multiple objectives, each of which can develop into separate articles. For instance, an IRB approved PhD research proposal often has one umbrella title covering various objectives that may lead to different articles.*<br><br>*In our case, the IRB-approved title includes two main objectives: compliance with smoke-free laws at HVs in Ethiopia and compliance with TAPS laws at points of sale in Ethiopia. Each objective addresses different target populations. Therefore, the research article focuses specifically on one of these objectives, which is compliance with smoke-free laws in HVs, resulting in a different yet related title. We hope this explanation clarifies the reason for the title difference.*	
32	…. between December 5th and 28th, 2022.	• Why it took long period of time? Too late?
	Author's Responses:<br>*Thank you for your comment regarding the timeline of our study. We would like to provide some context to clarify the process. Our data collection was completed by the end of 2022. Following this, we undertook data cleaning and analysis, and produced technical reports for both projects: Compliance with Smoke-Free Laws in HVs and Compliance with TAPS at Points of Sale during 2023. Manuscript development commenced at the beginning of 2024. Our current manuscript on Compliance with Smoke-Free Laws was finalized and submitted to PLOS One on July 23, 2024. It is still under review in 2025.*<br><br>*Given this tightened timeline situation, involving meticulous data processing and multiple project outputs, we believe our manuscript is timely and addresses the current state of compliance with smoke-free laws in Ethiopia.*	
41	Among 1,370 HVs included in this study, a total of 1,368 indoor places and 329 outdoor spaces were observed.	• Why not 1370? Why not 100%? Revisit of HVs done? Involuntary? Carelessness from your side?
	Author's Responses:<br>*Thank you for your comment regarding the number of observed HVs. We would like to clarify the figures presented in our study. Among the 1,370 HVs included in this study, 1,368 had indoor spaces, 327 had both indoor and outdoor spaces, and 2 had only outdoor spaces. Therefore, the number of HVs with indoor spaces cannot be 1,370 because 2 HVs had only outdoor spaces. This accounts for the difference and we would like to ensure that our data is accurate. However, we made some tweaks to the statement to make it clearer. We hope this explanation clarifies the figures and addresses your concerns.*	
55-56	Compliance with smoke-free laws in HVs remains low, particularly in bars, nightclubs/lounges, and Semera-Logia, with high rates of active smoking both indoors and outdoors.	• Do you have operational definition for low? Medium? High? Reference?
	Author's Responses:	

	Thank you for your valuable comment. We understand your concern regarding the use of the term "low" without an operational definition. The WHO and the Ethiopian Tobacco Control Proclamation require 100% smoke-free indoor public places and workplaces, including HVs. Therefore, our use of the term "low" is in reference to this 100% compliance requirement. Additionally, it is a relative term compared to other HVs, as some HVs show better compliance than others.	
72-74	However, studies in sub-Saharan Africa have indicated a lack of knowledge and low levels of compliance with smoke-free laws in HVs [8-10].	• You start with global case (GATS) and then move to Ethiopia case and here you come to Sub-Saharan case? Why? Message flow is not in descending order. Rather it is zigzag. Why? Lacks coherence?
	Author's Responses: Thank you for your valuable comment. We have rearranged this statement in the revised manuscript.	
107-113	**True compliance** refers to the law's effectiveness in preventing active smoking in prohibited areas and serves as a key indicator of its success. While, **adherence** encompasses how HVs comply with various provisions, such as displaying 'no smoking' signage, removing ashtrays and lighters, banning DSAs, absence of cigarette butts, prohibiting the sale of tobacco products in HVs, and forbidding tobacco use within a 10-meter radius of doorways, windows, or air intake mechanisms of public workplaces.	• You can add these under the definition of terms. I think this is not the proper place.
	Author's Responses: Thank you for your insightful comment regarding the placement of the definitions for "compliance" and "adherence." We understand your suggestion to add these under the definition of terms section. However, we believe that these terms are critical for understanding the context of our manuscript, and introducing them early on will guide readers from the beginning.	
121	Ethiopia had a population of about 120 million in 2022.	• Is this included under methods section? Why? If so, what is your reference for 120 M population?
	Author's Responses: Thank you for your comment regarding the population reference in the METHODS section. The population figure is presented to provide context within the "Study design, setting and population" subsection. We believe it is important to include this information to give readers an understanding of the study's setting and demographic context.<br><br>*We would also like to mention that the inclusion of the population figure was a recommendation from one of the reviewers during the initial review process. Regarding the reference for the population figure, we used data from the Central Statistical Agency (CSA), which estimated Ethiopia's population to be about 120 million in 2022: Central Statistical Agency (CSA): Population Projections for Ethiopia 2007-2037. 2013, Addis Ababa, Ethiopia.*	
137	cities, with the highest density of registered HVs, were selected from the list of sub-cities in each	• Selection bias? Result can be biased finding?
	Author's Responses:	

	Thank you for your comment regarding the potential for selection bias in our study. We understand your concern and would like to provide some context. Our sampling and selection of primary and secondary sampling units, including villages/neighborhoods, were based on the "Guides of Assessment of HVs for Smoke-Free Environment Studies," which is currently a standard for such studies. We aimed to evaluate compliance in areas with higher densities of HVs, as these areas are more likely to reflect the effectiveness of smoke-free laws.<br><br>*We acknowledge the potential for selection bias; however, the goal of our study was to assess compliance in high-density HV areas, which is consistent with the guidance provided in the referenced document: International Union Against Tuberculosis and Lung Disease. Assessing Compliance with Smoke-Free Laws: A "How-to" Guide for Conducting Compliance Studies. Second Edition. 2014; 1–36. We hope this explanation addresses your concern and clarifies our methodology.*	
149-150	Additionally, for certain HV categories with a limited number of venues, all identified HVs were included in the sample.	• So, can you compare this finding with highest density of registered HVs case? Are they comparable?
	Author's Responses:<br>*Thank you for your comment regarding the inclusion of certain HV categories with a limited number of venues. We understand the importance of ensuring our findings are comparable across different HV density cases.*<br><br>*In our study, for HV categories with a limited number of venues, all identified HVs were included in the sample to ensure a comprehensive assessment. This approach was taken to capture the variability and ensure the robustness of our findings. While the methodology differs from the selection of HVs in high-density areas, both approaches are designed to provide a representative understanding of compliance with smoke-free laws. We hope this explanation clarifies our approach and addresses your concern.*	
154-155	All HVs chosen for this study and providing services during the time of data collection were included in the study.	• What about if they are involuntary? Why couldn't you arrive at 100% response rate? Any problem?
	Author's Responses:<br>*Thank you for your comment regarding the eligibility criteria and response rate. We would like to provide some clarification. This study was based on covert observations, and we did not require consent from HVs to be included in the study. Refusal was very minimal. The statement "All HVs chosen for this study and providing services during the time of data collection were included in the study" reflects that HVs not providing service during the survey period were excluded from the very beginning.*<br><br>*Therefore, the 100% response rate could not be achieved due to the exclusion of HVs that were not operational at the time of data collection, rather than any involuntary refusal or problems encountered during the study.*	
167-168	Data were collected by 24 trained data collectors, with one pair of data collectors in each city, except for Addis Ababa, which had three pairs of data collectors.	• What is the Data collectors educational background? Work experience?
	Author's Responses:	

	Thank you for your comment regarding the educational background and work experience of the data collectors. Data were collected by 24 experienced data collectors, paired in each city (except for Addis Ababa, which had three pairs). All data collectors had a minimum of a BSc degree. There were 10 field supervisors (MPH/MSc and PhD holders) from the universities of the selected cities, one for each city. Field supervisors and data collectors were fluent in local languages, Amharic, and English. Most had prior experience with national surveys and data collection using electronic systems like ODK.<br><br>*While we appreciate your interest in the details of our data collection team, we chose not to include this information in the manuscript to maintain focus on the study's findings and methodology. We hope this information addresses your concerns.*	
171	..between December 5th and 28th, 2022, during peak business hours (17:00-24:00).	• Peak working area can have an impact on the results of the finding. Can we conclude the result of the finding for other cases?
	Author's Responses:<br>*Thank you for your comment regarding the potential impact of conducting observations during peak business hours. Most studies on smoke-free environments in HVs are conducted during these peak hours (17:00-24:00) to capture the highest levels of activity and thus provide a comprehensive assessment of compliance with smoke-free laws.*<br><br>*While our findings are based on observations during these peak hours, we believe they provide a reliable indication of overall compliance due to the higher likelihood of detecting violations when HVs are busiest. However, we acknowledge that conducting observations at different times might yield additional insights, but grossly underestimates the result. We hope this explanation clarifies our methodology and addresses your concern.*	
173	In some HVs, they also ordered beverages or food.	• Not relevant. You can delete it.
	Author's Responses:<br>*Thank you for your comment regarding the statement about ordering beverages or food in some HVs. We would like to provide some context for its inclusion.*<br><br>*This issue is very important for studies based on covert observations, especially at HVs during late hours of the day. It is often necessary to order meals or drinks to avoid drawing attention and to blend in as regular customers. Sometimes, particularly during late hours, meals or tea/coffee/soft drinks are unavailable, and most clients order alcoholic drinks, which can be expensive. Although this was not part of our initial plan, we found it critical for the success of the study. Therefore, we believe that this statement is relevant, particularly for researchers conducting similar studies, to facilitate their planning. We hope this explanation clarifies the importance of the statement.*	
207	Field personnel received training, and the checklists underwent pre-testing to ensure accuracy	• Where was pretest done? On how many % of your sample size? In which region/sub city?
	Author's Responses:<br>*Thank you for your comment regarding the pre-testing of our data collection tools. We would like to provide detailed context to address your questions.*<br><br>*Data collectors and supervisors received a three-day training in Addis Ababa, covering the study's aim, data collection tools, electronic data collection with smartphones/tablets, smoke-free laws, sampling*	

	procedures of HVs, pre-testing, data quality, and research ethics. Specific articles from the WHO FCTC law and the Ethiopian Tobacco Control Proclamation (1112/2019) were presented and discussed by experts from EFDA and Addis Ababa FMHACA. Training was by the research team, including the Project Lead, Project Co-Lead, and other experienced quantitative researchers. They were fluent in local languages, Amharic, and English, and almost all had prior experience with national surveys and data collection using electronic systems like ODK.<br><br>*The pre-testing was conducted in Nefas-Silk Lafto Sub-city, Woreda 08. Each pair of data collectors observed at least 2 HVs for pre-testing from 18:00 to 21:00. In total, the data collection tools were pre-tested on 25 HVs after completing the three-day training in Addis Ababa. Data collectors collected data, and issues related to specific questions were discussed and addressed. Based on the findings from pre-testing, appropriate amendments and revisions were made.*<br><br>*As senior researchers and professors at universities and research institutes as being team members, we are well aware of the importance of training and pre-testing in research and also how to conduct it. We hope this detailed explanation clarifies our methodology and addresses your concerns.*	
220	Further operational definitions used in this study are attached in S2 File.	• What about True compliance and adherence compliance? Are they included there?
	Author's Responses:<br>*Thank you for your comment regarding the inclusion of definitions for "true compliance" and "adherence compliance." As previously mentioned, these terms are defined at the end of the Introduction section to guide readers from the beginning of the manuscript. Therefore, they are not included in the S2 File, which contains further operational definitions used in this study.*	
245-46	The study protocol for the project titled "Compliance with Smoke-free Laws and Tobacco Advertisement, Promotion and Sponsorship Bans in Ethiopia"	• Your topic/title was **Compliance with smoke-free laws in hospitality venues in Ethiopia. Is it entirely the same? Any modification?**
	Author's Responses:<br>*Thank you for your comment regarding the study protocol title. The study protocol was initially titled "Compliance with Smoke-free Laws and Tobacco Advertisement, Promotion and Sponsorship Bans in Ethiopia," which encompasses a broader scope as it has been described earlier. However, our manuscript specifically focuses on compliance with smoke-free laws in HVs in Ethiopia. While the protocol covers various aspects of tobacco control, our paper zooms in on the compliance within HVs. We hope this explanation clarifies the connection between the protocol title and the manuscript title and addresses your concern.*	
258	Addis Ababa had the highest number of venues (20.8%), …..	• Are you comparing AA with regional towns? Comparable? On what ground/basis?
	Author's Responses:<br>*Thank you for your comment regarding the comparison of Addis Ababa with regional towns. We would like to clarify that the paragraph is intended to describe the distribution of our sample by cities, not to compare Addis Ababa with other regional cities. The distribution of HVs follows the proportional sizes of the cities' populations. The details of the data are presented in Table 1, which shows the distribution of HVs across the cities. We hope this explanation clarifies our intent and addresses your concern.*	
268	(n=430) from cigarettes. Although shisha use was rare overall in indoor spaces (1.5%), but it was…..	• What was your professional suggestion for these shisha users? You keep silent at all? Why?

<table>
<tr><td></td><td colspan="2">Author’s Responses:
Thank you for your comment regarding shisha use in indoor spaces. Our study focused on observing compliance with smoke-free laws and documenting instances of active smoking, including shisha use. While we observed shisha use in nightclubs/lounges, our role was to document compliance, and we did not provide direct interventions or suggestion on shisha use in the RESULTS section. We hope this explanation clarifies our approach and addresses your concern.</td></tr>
<tr><td>372</td><td>….across various HVs and cities, which is higher than a previous Ethiopian study from 2021 that</td><td>• Your study and this study are after the proclamation was formulated. What do you think is the root cause for this gap?</td></tr>
<tr><td></td><td colspan="2">Author’s Responses:
Thank you for your comment regarding the gap in active smoking rates between our study and the previous Ethiopian study from 2021. The observed discrepancy may arise from differences in observation timing, variations in venue types, and the higher prevalence of smoking in the cities included in our study compared to the regions assessed in the previous study. To clarify, we have modified the original statement in the revised manuscript as follows:

“This discrepancy may arise from differences in observation timing, variations in venue types, and the higher prevalence of smoking in the cities included in our study”.</td></tr>
<tr><td>478</td><td>Conclusions</td><td>• This study aims to study/ evaluate the extent of compliance with smoke-free laws in HVs and identify factors associated with non-compliance. But, you don’t have no conclusion about the factors associated with non-compliance</td></tr>
<tr><td></td><td colspan="2">Author’s Responses:
Thank you for your comment regarding the conclusion of our study. We have revised the conclusion to explicitly address the factors associated with non-compliance, including the lack of enforcement of the 'no smoking within 10-meter' requirement, the presence of lighters, and the sale of tobacco products. We believe the revised conclusion now adequately addresses your concern.</td></tr>
<tr><td>480</td><td>….. and Semera-Logia, with high rates of active smoking both indoors and outdoors.</td><td>• Comparing AA with this site/area is not feasible and they are not comparable sites at all.</td></tr>
<tr><td></td><td colspan="2">Author’s Responses:
Thank you for your comment regarding the comparison of cities. We would like to clarify that we are not comparing the cities directly; rather, we are analyzing the data based on the sample obtained from various cities. Our intent is to present the distribution and compliance rates of HVs within our study sample. We hope this explanation clarifies our approach and addresses your concern.</td></tr>
</table>
