# Supplementary material for: Compliance with smoke-free laws in hospitality venues in Ethiopia: A cross-sectional observational study in 10 cities
Source: PLoS One. 2025 Feb 21;20(2):e0319079. doi: 10.1371/journal.pone.0319079 (PMC11844872; doi:10.1371/journal.pone.0319079)
Supplement: Attachment — Submitted filename: Ethiopia SFE 2 review.docx [file pone.0319079.s007.docx]

| **LINE** | **ORIGINAL IN THE DOCUMENT WITH TRACK CHANGES** | **COMMENTS** |
| --- | --- | --- |
| 96-97 | However, studies in sub-Saharan Africa have indicated a lack of knowledge and low levels of compliance with smoke-free laws in HVs [8-10]. | It is not clear what the “lack of knowledge” refers to. Lack of knowledge of the health effects of SHS? Lack of knowledge of compliance? If this second situation is what it refers to, then it does make sense that studies don´t know what compliance is AND at the same time, they know that compliance is low. Please clarify. |
| 99 | Studies indicate a high prevalence of indoor smoking in HVs [8, 11, 12], | Studies where? In Africa? Worldwide? Please clarify |
| 122-123 | One of the two studies only studied five government hospitals in Addis Ababa [20] | Avoid repetition of the word study for better style. Perhaps write: “One of the two studies was restricted to five government hospitals in Addis Ababa.” |
| 142-143 | In this study, we assessed the implementation of smoke-free laws, focusing on two related concepts: true compliance and adherence to the law’s requirements. | The two related concepts are compliance and adherence to the law. There is no true compliance as opposed to false compliance. I suggest writing “… focusing on two related concepts: compliance with the smoking ban and adherence to its other requirements.” Please do not use “true” to refer to compliance. |
| 195 | We then used the comprehensive mapped lists to select HVs, either through systematic or random sampling. | Please clarify when you used systematic sampling and when you used random sampling. |
| 196 | In areas where the total number of HVs was small,… | You may want to clarify your criteria for a small n. For example, you could write, “In areas where the total number of HVs was small (n<x),…” |
| 227 | Data collectors observed compliance with the presence of "no smoking" signage at the main entrance and inside the venue,… | The use of “compliance” is not appropriate. I suggest rephrasing as “Data collectors observed if "no smoking" signage was properly displayed at the main entrance and inside the venue,...”. |
| 229 & 231 | They also noted if anyone was smoking within 10 meters of any main door, window, or air intake mechanism. They also asked the waiter/waitress if t | For style reasons, avoid repeating “they also” in L231. Perhaps use “In addition they…” |
| 245 | 9) no one was seen smoking used tobacco products in the outdoor area within 10- meters from any door, window, or air intake mechanism (0=no,  1=yes) | Remind the reader that indicator nine was obtained even if the venue did not have an outdoor area to serve the public. |
| 247 | The first indicator measures the true compliance with smoke-free law, … | As indicated before, I suggest dropping the term true to refer to compliance. I suggest rewriting. “The first indicator measures the compliance with smoke-free law,…” |
| 250 | Outdoor space compliance: The following five smoke-free specific indicators were used to assess the outdoor space compliance and adherence: | Conceptually, these indicators can't measure compliance or adherence to the law because the law does not ban smoking outdoors. I suggest rewriting: “Outdoor Smoking: We also conducted an assessment of smoking-related activity in all outdoor spaces where smoking is permitted. The following five indicators were used: …” |
| 332  344 | Section on Indoor active smoking and enabling factors  Section on Compliance and adherence with smoke- free laws in indoor areas | These two sections contain the same information. The first presents it as noncompliance and nonadherence, and the second as compliance and adherence. I suggest keeping only one, perhaps as “Compliance with the law and adherence to the law requirements in indoor areas,” since Table 2 presents compliance and adherence data. |
| 353 | Results | Make it more straightforward when you talk about compliance and when you do about adherence. |
| 363 | Table 2. Compliance and adherence with smoke-free laws indicators in indoor areas by cities and HVs | I suggest tweaking the title of Table 2 and saying, “Compliance with the law and adherence to the law requirements in indoor areas by city and HV type.” |
| 283 &  377 | Data processing and analysis  Table 3. Indoor compliance level with smoke-free laws by cities and HVs | Now that you have adopted the concepts of compliance with the law and adherence to its requirements, the classification of HVs into levels of compliance and Table 3 does not make much sense. Compliance should be measured ONLY by seeing active smoking. Then, you could classify levels of adherence to the requirements of the law as ‘fully adherent,’ ‘good adherence,’ ‘moderately adherent,’ and ‘poorly adherent. ‘  I suggest rewriting the piece in the section on data processing and analysis as follows:  We classified each city and HV type as compliant with the law when no active smoking was detected and as noncompliant when it was detected. In addition, Indoor venues were classified as ‘highly adherent,’ ‘moderately adherent, and ‘poorly adherent’ with the legal smoke-free requirements when they met 7-8, 5-6, and > 5 of these requirements, respectively.  Accordingly, I also suggest rewriting Table 3 with a new appropriate title and only three levels of adherence. Also, rewrite lines 374-376 to describe results and adherence from Table 3. |
| 287 &  417 |  | As indicated before, given that the law doesn’t ban smoking in outdoor areas, it doesn't make sense to talk about compliance with or adherence to the law in these areas. For this reason, II would suppress lines 287-291 in methods and lines 417-418 along with Table 5 in results. |
| 383 & 394 | Section on Outdoor active smoking and enabling factors  Section on Compliance and adherence with smoke-free laws in outdoor spaces | These two sections contain almost the same information. The first presents it as negative behavior (smoking) and “no smoking” signage, while the second is as positive behavior (non-smoking) and absence of ashtrays. I suggest keeping Table 4 and retitling it as “Smoke-free indicators in outdoor areas by city and HV type” and combining both sections to describe no-smoking behavior, as well as the other four indicators.  By the way, the results for the NoDSA indicator are not described in the results section text. Should the NoDSA requirement for outdoor areas be analyzed at all? While a NoDSA requirement for indoor spaces is clearly positive, its value for outdoor areas where smoking is permitted is more controversial. On the one hand, it may transmit that non-smokers need to be protected in DSA outdoors (which is positive for public health). At the same time, because they are ineffective in protecting non-smokers from SHS, they may be considered an excuse not to ban smoking outdoors (which is negative for public health). For these reasons, I wonder if the NoDSA indicator should not be analyzed at all and should be dropped. |
| 427 | Section on Factors associated with active smoking and cigarette butts in indoor places | The section analyzes the factors (law requirements) associated with active smoking and cigarette butts separately. The authors do not justify why in addition to active smoking, they also investigate the association with the finding of butts. Please justify it.  Also, I wonder why they don't investigate the association between active smoking and finding butts. |
| 441-442 | … before and after adjustment. | Please describe in the text or Table 6 the variables for which you adjusted. |
| 454 | Table 6. | The last six rows of Table 6 reflect factors that represent law violations. The row on smoking signs should also be phrased and analyzed as a violation of the law (absence of signs) so that the PRs can show the same direction as the other violators and make them more comparable. |
| 474 | The higher prevalence of outdoor smoking in our study suggests that smokers may be shifting to outdoor areas in response to indoor smoking bans. | In my view, the data does not necessarily suggest the migration of smokers. I suggest rephrasing it as a hypothesis to be studied. The data would suggest a migration of smokers only if the number of venues with outdoor smoking had increased after the entry into force of the indoor ban. Otherwise, the prevalence of smoking might have been high before the indoor ban. Since the study does not present the before and after the ban data, “suggests” is too strong a word. |
| 366  390 | While adherences were notably low for cigarette butts  However, these findings must be viewed alongside lower compliance rates for key indicators like active smoking and cigarette butts, | In L366, the presence of cigarette butts is presented as an adherence factor, while in L391, it is presented as a key indicator for compliance. Please, choose one term to depict the presence of cigarette butts and use it consistently. |
| 485-488 | Interestingly, our study found exceptionally high adherence rates regarding the absence of DSAs and shisha equipment, indicating that smoke-free legislation is effective in specific contexts where enforcement and public health messaging are prioritized, | I would use a more tentative language since you do not have a before-after design to indicate if SF legislation has reduced DSAs and shisha equipment. “suggesting that smoke-free legislation might be effective” |
| 506 | The presence of 'no smoking' signage was significantly associated with reduced active smoking rates and the prevalence of cigarette butts. | This statement is factually incorrect. First, the association is not with smoking rates but the prevalence of venues with active smoking in violation of the ban. Second, the aPR for active smoking is 1.3 (presented as 0.77) and not significant for the presence of butts. An aPR of 1.3 (95% CI 1.1-1.5) is not that impactful, particularly compared to the other violation factors. Given that the following sentence is offered as a contrast, this sentence seems involuntarily erroneous. Please correct. |
| 515 | our study showed a significant association between such signage and decreased active smoking rates and cigarette butt prevalence. | The same error as above is repeated here. |
| 518 | These results highlight the necessity for … enhanced visual enforcement, such as prominent ‘no smoking’ signage. | Given the above two comments, this conclusion does not seem warranted. |
| 528 | In our study, full compliance with smoke-free laws was observed…. | To be coherent with the distinction between compliance and adherence, the sentence should be written as ”In our study, compliance with smoke-free law along with full adherence to its requirements was observed…” |
| 555-565 | The findings of this study have significant policy implications for enhancing tobacco control efforts in Ethiopia,… Increasing the visibility and enforcement of ‘no smoking’ signage can serve as an effective policy measure…. | In my view, the small association found in the study between the absence of “no smoking” signage and indoor active smoking does not justify the strong emphasis on enforcing such signage. According to Table 6, the three factors more strongly associated with the detection of active smoking in indoor places where smoking is banned are smoking within 10 m, the presence of lighters, and sales of tobacco products. Given that non-compliance with the “*no smoking within 10 m*” requirement of the law is high, around 50%, focusing on enforcement of this requirement seems advisable when designing implementation efforts, either through enforcement authorities or public education. Perhaps this advice should be clearly discussed.  If the authors still want to recommend the enforcement of signage, they could say that despite the results of this study, the international experience shows the importance of such signage. |
| 577-585 | Conclusions. … alongside enhanced enforcement of 'no smoking’ signage… | Same comment as above. |
