# Supplementary material for: Compliance with smoke-free laws in hospitality venues in Ethiopia: A cross-sectional observational study in 10 cities
Source: PLoS One. 2025 Feb 21;20(2):e0319079. doi: 10.1371/journal.pone.0319079 (PMC11844872; doi:10.1371/journal.pone.0319079)
Supplement: Attachment — Submitted filename: Manuscript 2.docx [file pone.0319079.s008.docx]

**Compliance with smoke-free laws in hospitality venues in Ethiopia: a cross-sectional observational study in 10 cities**

Wakgari Deressa^1*^, Selamawit Hirpa^1^, Terefe Gelibo Argefa^2,3^, Yifokire Tefera^1^, Selam Abraham Kassa^2^, Rachel Kitonyo-Devotsu^2^, Winnie Awuor^2^, Baharu Zewdie^4^, Noreen Dadirai Mdege^2,5,6^

^1^Department of Preventive Medicine, School of Public Health, College of Health Sciences, Addis Ababa University, Addis Ababa, Ethiopia

^2^Development Gateway: an IREX Venture, Washington DC, United States of America

^3^ICAP at Columbia University, Mailman School of Public Health, Addis Ababa, Ethiopia

^4^Tobacco Control Unit, Ethiopian Food and Drug Authority, Addis Ababa, Ethiopia

^5^Department of Health Sciences, University of York, York YO10 5DD, United Kingdom

^6^Centre for Research in Health and Development, York, United Kingdom

*Corresponding author

Email: [deressaw@gmail.com](mailto:deressaw@gmail.com) (WD)

Short title: Smoke-free Law Compliance in Ethiopia

# **Abstract**

**Background**

The 2019 Ethiopia's comprehensive tobacco control proclamation mandates 100% smoke-free public places and workplaces. Despite the proclamation, compliance remains uncertain, particularly at hospitality venues (HVs). The aim of this study was to evaluate the extent of compliance with smoke-free laws in HVs and identify factors associated with non-compliance.

**Methods**

This cross-sectional observational study was conducted in 10 cities in Ethiopia—Addis Ababa, Adama, Assosa, Bahir Dar, Dire Dawa, Gambella, Harar, Hawassa, Jigjiga, and Semera-Logia—between December 5^th^ and 28^th^, 2022. Data were collected electronically using smartphones, utilizing a pre-tested, standardized checklist and covert observation. The subjects were selected through multi-stage cluster sampling. A total of 1,370 HVs (hotels, restaurants, bars, bars and restaurants, café and restaurants, butcher houses and restaurants, groceries, and nightclubs/ lounges) were observed. Specific and composite compliance indicators were computed for indoor and outdoor spaces. Poisson regression analyses identified factors associated with indoor active smoking and non-compliance. Statistical significance was set at *P*<0.05. Data were analyzed using SPSS version 26.

**Results**

Among 1,370 HVs included in this study, a total of 1,368 indoor places and 329 outdoor spaces were observed. Active smoking was observed in 32.2% (95% CI:30-35) of indoor HVs, with the highest rates in nightclubs/lounges (68.6%) and bars (65.7%). Semera-Logia reported the highest prevalence of active smoking (70.4%). Adherence with ‘no smoking’ signage was low (35.2%), while ashtrays, lighters, and designated smoking areas (DSAs) were rarely present. Outdoor active smoking was observed in 46.5% (95% CI:41-52) of HVs. Only 12.5% of indoor and 10.3% of outdoor spaces were fully compliant. Venues in Semera-Logia were over twice as likely to have active smoking (adjusted prevalence ratio [aPR]: 2.71; 95% CI: 2.00–3.66) compared to Addis Ababa. Bars and nightclubs/lounges had significantly higher prevalence of active smoking than cafés/restaurants. ‘No smoking’ signs were associated with reduced prevalence of indoor active smoking (aPR: 0.77; 95% CI: 0.67–0.89), while lighters (aPR: 1.69; 95% CI: 1.41–2.02) and tobacco product sales (aPR: 1.58; 95% CI: 1.34–1.86) were associated with higher prevalence.

**Conclusion**

Compliance with smoke-free laws in HVs remains low, particularly in bars, nightclubs/lounges, and Semera-Logia, with high rates of active smoking both indoors and outdoors. Enhanced enforcement and targeted are needed to educate the public and HV owners about the risks of SHS and the importance of tobacco control laws.

## Introduction

According to the Global Adult Tobacco Survey (GATS) conducted in 2016 in Ethiopia, 3.7% (approximately 2.5 million) of adults (6.2% of men and 1.2% of women) were current cigarette smokers [1], and the smoking prevalence is predicted to rise [2-4]. Exposure of non-smokers to the secondhand tobacco smoke (SHS) is a major concern [5]. From Ethiopia’s 2016 GATS, 31% of adults aged 15 years and older who visited restaurants, and 60% who visited bars/nightclubs, were exposed to SHS from tobacco in the 30 days preceding the survey [1]. It has been demonstrated that comprehensive smoke-free legislation effectively reduces SHS exposure among customers, bartenders and workers at hospitality venues (HVs) [6]. Furthermore, it reduces smoking among adolescents and young people [7]. However, studies in sub-Saharan Africa have indicated a lack of knowledge and low levels of compliance with smoke-free laws in HVs [8-10].

Studies indicate a high prevalence of indoor smoking in HVs [8, 11, 12], with workers in bars and nightclubs experiencing higher levels of SHS exposure [13, 14] and a greater risk of lung cancer mortality [15]. This environment not only endangers workers but also affects vulnerable populations, as children living with a smoker have a significantly higher prevalence of asthma and wheeze, underscoring the urgent need to prevent SHS exposure to safeguard respiratory health [16, 17].

Ethiopia enacted a new comprehensive tobacco control law in 2019 (Proclamation No.1112/2019) [18]. The proclamation requires 100% smoke-free indoor public places and workplaces including HVs. Smoking and the use of tobacco products are prohibited in all indoor workplaces, public places, public institutions, modes of transportation, and communal areas. The proclamation also prohibits designated smoking areas (DSAs) in public places. Additionally, it prohibits the use of any tobacco product in outdoor spaces within a 10-meter radius of any public place or workplace doorway, window, or air intake mechanism, thereby ensuring a comprehensive smoke-free environment. Furthermore, the proclamation mandates that the owner of the public place or another authorized person must post a ‘no smoking’ signage and prohibit tobacco use or sale in any part of indoor workplaces. Adherence to the law’s requirements involves implementing comprehensive measures designed not only to prevent active smoking in prohibited areas but also to eliminate enabling factors that may undermine a smoke-free environment [18].

A recent scoping review on SHS and smoke-free environments in Ethiopia identified only two studies that assessed compliance with smoke-free laws [19]. One of the two studies only studied five government hospitals in Addis Ababa [20] and was conducted prior to the enactment of Proclamation No.1112/2019 [11]. The second study was conducted in 2021 in public places such as those providing food and beverages, schools, health facilities, government offices, youth centers, parks and transit facilities in four regions [10].

Compliance with smoke-free laws is a key pillar of tobacco control policy implementation; however, there is a scarcity of evidence on the extent of compliance with smoke-free laws in Ethiopia, particularly in HVs, which exacerbates the health risks associated with SHS exposure. Therefore, we conducted an assessment of compliance with smoke-free laws in HVs in Ethiopia to provide decision-makers with the evidence they need to strengthen their efforts in protecting non-smokers from exposure to SHS.

In this study, we assessed the implementation of smoke-free laws, focusing on two related concepts: true compliance and adherence to the law’s requirements. **True compliance** refers to the law’s effectiveness in preventing active smoking in prohibited areas and serves as a key indicator of its success. While, **adherence** encompasses how HVs comply with various provisions, such as displaying 'no smoking' signage, removing ashtrays and lighters, banning DSAs, absence of cigarette butts, prohibiting the sale of tobacco products in HVs, and forbidding tobacco use within a 10-meter radius of doorways, windows, or air intake mechanisms of public workplaces. By distinguishing between these concepts, this study aims to clarify the true effectiveness of smoke-free legislation.

# **Methods**

**Study design, setting and population**

This cross-sectional study used covert observations to determine compliance with smoke-free laws at HVs in 10 purposively selected major regional and chartered cities in Ethiopia: Addis Ababa, Adama, Assosa, Bahir Dar, Dire Dawa, Gambella, Harar, Hawassa, Jigjiga, and Semera-Logia (where Semera and Logia are two separate nearby cities merged as one study area). Ethiopia had a population of about 120 million in 2022. The selected cities are characterized by diverse demographic and geographic features, high population density, fast economic development, and high smoking prevalence [1]. They are home to a population of over seven million people. The Tigray Regional State was not included in the study because of security problem at the time of the study.

**Sample size and sampling procedures**

We estimated the minimum sample size required for the study using a single proportion sample size formula. We considered a study conducted in Uganda, which found that 82% of the surveyed HVs complied with absence of ‘active smoking’ in the venues [8]. Based on 95% confidence level, 3% margin of error, design effect of two, and 5% non-response rate, a minimum of 1,300 HVs were required for the study. The sample size was allocated to the study cities and the selected sub-cities, woredas and kebeles within them proportionally to the estimated number of the HVs.

Our sampling procedures followed recommendations for studies on compliance with smoke-free laws [21]. Four of the 10 cities (Addis Ababa, Adama, Bahir Dar and Hawassa) are sub-divided into sub-cities, which are further sub-divided into woredas/kebeles. Two to six sub-cities, with the highest density of registered HVs, were selected from the list of sub-cities in each of these four cities in consultation with the city’s tobacco control law enforcement team (S1 Fig). Similarly, from the list of kebeles in each selected sub-city in Adama, Bahir Dar, and Hawassa, two kebeles with the highest density of registered HVs were chosen. In the remaining cities, i.e., Jigjiga, Semera-Logia, Dire Dawa, Harar, Assosa and Gambella, where there are no sub-cities/woredas, we selected 2-6 kebeles/woredas, focusing on those with the highest concentration of HVs.

We obtained initial lists of registered HVs in each selected kebele/woreda from the relevant local offices. Due to incomplete data, we supplemented these lists by conducting on-site mapping, walking street by street within each selected kebele/woreda to identify and list additional HVs. We then used the comprehensive mapped lists to select HVs, either through systematic or random sampling. In areas where the total number of HVs was small, we included all identified HVs. Additionally, for certain HV categories with a limited number of venues, all identified HVs were included in the sample. The assigned sample size for each kebele/woreda was proportionally distributed across the different HV types to ensure representation.

**Eligibility criteria**

All HVs chosen for this study and providing services during the time of data collection were included in the study.

**Data collection tools and procedures**

This study used a standardized observation checklist to guide covert observations. The observation checklist was developed based on the “How-to-Guide for Conducting Compliance Studies” for Smoke-Free Law [21]. We adapted the checklist considering the provisions of the Ethiopian Tobacco Control Proclamation (1112/2019) [18] and the 2021 tobacco control directive [22]. The tool was initially developed in English, then translated into Amharic, and back-translated into English to ensure consistency. The data collection tool (S1 File) was reviewed and enriched by tobacco control experts in Ethiopia, including those from the Ethiopian Food and Drug Authority (EFDA).

Data were collected by 24 trained data collectors, with one pair of data collectors in each city, except for Addis Ababa, which had three pairs of data collectors. There were 10 field supervisors, one for each city. A three-day training was held in Addis Ababa for all data collectors and supervisors. The data were collected electronically using Open Data Kit (ODK) between December 5^th^ and 28^th^, 2022, during peak business hours (17:00-24:00).

After scanning the outdoor area and entering the selected HV, the data collectors took a seat as customers, preferably in the main room. In some HVs, they also ordered beverages or food. The specific areas of the HVs that were covertly observed included main entrance, porch, verandah, main room, and two additional rooms, depending on the size of the venue. Other areas observed were lobby areas, corridor areas, hand washing and toilet areas.

Data collectors observed compliance with the presence of "no smoking" signage at the main entrance and inside the venue, whether anyone was smoking a tobacco product indoors or outdoors, and the presence of ashtrays, lighters, cigarette butts, and shisha equipment. They also noted if anyone was smoking within 10-meter range of any main door, window, or air intake mechanism. They also asked the waiter/waitress if the venue sold tobacco products, posing as smokers. If the staff said yes, the data collectors asked to see the available products and their prices. They purchased cigarettes and requested an area to smoke in the venue to check for a DSA. On average, they spent about 30 minutes at each venue. The data collectors entered the data into the smartphone/tablet while they were in or near the HVs without the knowledge of the HV owners or staff members. This approach improved the reliability and validity of the collected data [23].

**Smoke-free compliance and adherence indicators**

**Indoor space compliance**: The following nine smoke-free specific indicators were used to assess the indoor area compliance and adherence: 1) no one was seen smoking a tobacco product in the venue’s indoor space (0=no, 1=yes), 2) no ashtrays or other instruments used to hold cigarette ash inside (0=no, 1=yes), 3) no lighter was visible inside (0=no, 1=yes), 4) display of ‘no smoking’ signage (0=no, 1=yes), 5) absence of DSA) (0=no, 1=yes), 6) absence of shisha equipment (0=no, 1=yes), 7) absence of cigarette butts, 8) no tobacco product sales (0=no, 1=yes) and 9) no one was seen smoking tobacco products in the outdoor area within 10-meter range from any door, window, or air intake mechanism (0=no, 1=yes). The first indicator measures the true compliance with smoke-free law, while the remaining indicators assess adherence to the law’s requirements.

**Outdoor space compliance**: The following five smoke-free specific indicators were used to assess the outdoor space compliance and adherence: 1) no one was seen smoking tobacco product in the venue's outdoor space (0=no, 1=yes), 2) no ashtray or other instrument used to hold cigarette ash in the outdoor space (0=no, 1=yes), 3) display of ‘no smoking’ signage in the outdoor space (0=no, 1=yes), 4) no cigarette butts seen outdoor, and 5) absence of DSA in the outdoor space (0=no, 1=yes).

**Data quality control**

Field personnel received training, and the checklists underwent pre-testing to ensure accuracy and reliability. Data were collected electronically using ODK. Field supervisors reviewed and checked at least 5% of the data collected by each data collector. Supervisors were also actively involved in data collection alongside data collectors, resolving any inconsistencies through discussions. Each day, collected data were uploaded to a central server at Addis Ababa University, where they underwent daily reviews for completeness and consistency, with immediate feedback from the data manager.

**Operational definitions**

Active smoking means someone being in possession or control of a lit tobacco product, including cigarettes, cigars, and shisha, inside or outside of HVs at the time of data collection. An HV is defined as an establishment that is registered under the regulation of the Government of Ethiopia where food and beverages are sold and consumed, namely hotels, restaurants, bars, bars and restaurants, café and restaurant, butcher shop and restaurant, grocery, nightclub and lounges. Further operational definitions used in this study are attached in S2 File.

**Data processing and analysis**

Descriptive analysis was used to summarize the characteristics of the HVs, as well as compliance estimates for each smoke-free compliance indicator. We classified each HV as ‘fully compliant’, ‘good compliance’, ‘moderately compliant’, and ‘poorly compliant’ with smoke-free laws. An HV was categorized as ‘fully compliant’ for indoor space if it complied with all nine indicators, ‘good compliance’ for complying with 7-8 indicators, ‘moderately compliant’ for complying with 5-6 indicators, and ‘poor compliance’ if it met less than four indicators. For outdoor areas, an HV was classified as ‘fully compliant’ for outdoor area if it complied with all five indicators, ‘good compliance’ if it met four indicators, ‘moderate’ compliance if it met three indicators, and ‘poor compliance’ if it only complied with less than three indicators.

Sub-analyses were carried out by study city and HV types. Continuous variables were described using means and standard deviations, while categorical variables were characterized using frequencies and proportions. Chi-squared test was used to compare percentages between the study cities and venue types. The study investigated factors such as city, type of HVs, and the adherence factors associated with indoor active smoking (yes/no) and presence of cigarette butts (yes/no) using Poisson regression analyses with log link function and robust variance [24] and calculated crude prevalence ratios (cPR) and adjusted prevalence ratios (adjPR) with 95% confidence intervals (CIs). Collinearity between cities and HV types was assessed by calculating the Variance Inflation Factor (VIF), and no evidence of multicollinearity was found (VIF < 2). We calculated standard errors, taking into account the complex sample design, which involved adjusting for stratification by city and clustering of HVs at the kebele/cluster level. *P<0.05* was used for statistical significance. Data were analyzed with SPSS version 26 (IBM SPSS Statistics for Windows, Armonk, NY, USA).

## Ethics considerations

The study protocol for the project titled ‘‘Compliance with Smoke-free Laws and Tobacco Advertisement, Promotion and Sponsorship Bans in Ethiopia” was approved by the Institutional Review Board (IRB) of the Ethiopian Public Health Association (EPHA) (Ref. no. EPHA/OG/201/22; dated November 18, 2022). Due to the covert observational nature of the study, informed consent from the HVs or individuals was not obtained. The IRB granted a waiver of informed consent. The observation checklists did not include names, addresses, or any other identifying information, ensuring anonymity, privacy, and data confidentiality. No minors were involved in the study.

**Results**

## Characteristics of hospitality venues

Data were collected from a total of 1,370 HVs in 10 cities. About 76% (n=1,041) of the 1,370 HVs only had indoor spaces, whereas 23.9% (n=327) had both indoor and outdoor spaces, while two (0.1%) had only outdoor areas. Addis Ababa had the highest number of venues (20.8%), followed by Adama (11.4%), Hawassa (11.3%), and Bahir Dar (10.7%) (Table 1). The remaining six cities were represented by between 7.4% and 7.9% of the HVs. Hotels accounted for 19.3% of all venues, followed by bars and restaurants (17.4%), cafés and restaurants (14.8%), and restaurants (14.7%).

**Table 1. Types of hospitality venues by city**

| **City** | **Venue type, n (%)** | | | | | | | | **Total, n (%)** |
| --- | --- | --- | --- | --- | --- | --- | --- | --- | --- |
|  | **Hotel** | **Bar and restaurant** | **Café and restaurant** | **Restaurant** | **Grocery** | **Butcher house and restaurant** | **Bar** | **Nightclub /lounge** |  |
| Addis Ababa | 27 (9.5) | 73 (25.6) | 37 (13.0) | 42 (14.8) | 30 (10.5) | 4 (1.4) | 45 (15.8) | 27 (9.5) | 285 (20.8) |
| Adama | 28 (17.9) | 38 (24.4) | 32 (20.5) | 10 (6.4) | 16 (10.3) | 22 (14.1) | 7 (4.5) | 3 (1.9) | 156 (11.4) |
| Hawassa | 23 (14.9) | 53 (34.4) | 17 (11.0) | 18 (11.7) | 21 (13.6) | 1 (0.6) | 14 (9.1) | 7 (4.5) | 154 (11.3) |
| Bahir Dar | 21 (14.3) | 9 (6.1) | 17 (11.6) | 38 (25.9) | 19 (12.9) | 13 (8.8) | 18 (12.2) | 12 (8.2) | 147 (10.7) |
| Jigjiga | 46 (42.6) | 0 | 22 (20.4) | 22 (20.4) | 7 (6.5) | 11 (10.2) | 0 | 0 | 108 (7.9) |
| Semera-Logia | 23 (21.3) | 1 (0.9) | 47 (43.5) | 37 (34.3) | 0 | 0 | 0 | 0 | 108 (7.9) |
| Harar | 14 (13.3) | 11 (10.5) | 19 (18.1) | 0 | 4 (3.8) | 49 (46.7) | 2 (1.9) | 6 (5.7) | 105 (7.7) |
| Dire Dawa | 18 (17.5) | 21 (20.4) | 2 (1.9) | 5 (4.9) | 29 (28.2) | 8 (7.8) | 12 (11.7) | 8 (7.8) | 103 (7.5) |
| Assosa | 33 (32.0) | 15 (14.6) | 8 (8.8) | 4 (3.9) | 25 (24.3) | 10 (9.7) | 1 (1.0) | 7 (6.8) | 103 (7.5) |
| Gambella | 31 (30.7) | 17 (16.8) | 3 (3.0) | 25 (24.8) | 16 (15.8) | 6 (5.9) | 3 (3.0) | 0 | 101 (7.4) |
| **Total, n (%)** | **264 (19.3)** | **238 (17.4)** | **204 (14.8)** | **201 (14.7)** | **167 (12.2)** | **124 (9.0)** | **102 (7.4)** | **70 (5.1)** | **1,370 (100.0)** |

**Indoor active smoking and enabling factors**

Active smoking was observed in indoors of 441 [32.2% (95% CI:30-35)] HVs, with 97.3% (n=430) from cigarettes. Although shisha use was rare overall in indoor spaces (1.5%), but it was predominantly observed in indoor areas of nightclubs/lounges (52.4%). Active smoking was the highest in the indoor spaces of HVs in Semera-Logia (70.4%) and Dire Dawa (61.2%). The highest rates of observed active smoking were in the nightclubs/lounges (68.6%) and bars (65.7%). The proportion of HVs that posted ‘no smoking signage’ in indoor spaces was 35.2% (95% CI:33.0-38.0), with the highest proportion in Semera-Logia (66.7%), whilst this was lowest in Jigjiga (8.3%). Ashtrays, lighters, and shisha equipment were rarely found in the indoor venues across cities, although lighters were observed in 34% of venues in Dire Dawa. The presence of DSAs was generally low, with 8.2% of the HVs in Adama, 5.1% in Semera-Logia, and 2.1% in Bahir Dar.

### Compliance and adherence with smoke-free laws in indoor areas

Table 2 shows compliance and adherence with indoor smoke-free indicators across cities and HVs. The highest adherence was for the absence of DSAs (98.2%) and shisha equipment (98%), while compliance was lower for no active smoking (67.8%). Absence of cigarette butts (64.0%). 'No smoking' signage (64.8%) were the least adhered, and half of the venues had no outdoor smoking within 10-meter range of doors or windows. Adherence with no tobacco product sales was generally high, with Jigjiga showing full adherence, but it was lower in Semera-Logia (46.3%) and among nightclubs/lounges (50%). Most HVs showed high adherence for the absence of ashtrays and lighters, but these enabling aids were more common in bars and nightclubs/lounges. Overall, compliance varied by venue type, with cafés and restaurants showing high adherence, while bars and nightclubs/lounges had the lowest compliance or adherence rates for key indicators.

**Table 2. Compliance and adherence with smoke-free laws indicators in indoor areas by cities and HVs**

| **City (n=1368)** | **Indoor smoke-free indicator, n (%)** | | | | | | | | |
| --- | --- | --- | --- | --- | --- | --- | --- | --- | --- |
|  | **No active smoking** | **‘No smoking’ signage** | **No ashtray** | **No lighter** | **No DSA^a^** | **No cigarette butts** | **No shisha equipment** | **No tobacco product sale** | **No smoking within 10m** |
| Addis Ababa (n=284) | 209 (73.6) | 152 (53.5) | 271 (95.4) | 255 (89.8) | 284 (100.0) | 211 (74.3) | 276 (97.2) | 279 (98.2) | 131 (46.1) |
| Adama (n=156) | 117 (75.0) | 50 (32.4) | 145 (92.9) | 148 (94.9) | 143 (91.7) | 130 (83.3) | 154 (98.7) | 139 (89.1) | 100 (64.1) |
| Hawassa (n=154) | 112 (72.7) | 45 (29.2) | 141 (91.6) | 121 (78.6) | 152 (98.7) | 111 (72.1) | 149 (96.8) | 128 (83.1) | 68 (44.2) |
| Bahir Dar (n=146) | 113 (77.4) | 19 (13.0) | 137 (93.8) | 130 (89.0) | 143 (97.9) | 111 (76.0) | 141 (96.6) | 88 (60.3) | 111 (76.0) |
| Jigjiga (n=108) | 100 (92.6) | 9 (8.3) | 108 (100.0) | 108 (100.0) | 108 (100.0) | 90 (83.3) | 108 (100.0) | 108 (100.0) | 93 (86.1) |
| Semera-Logia (n=108) | 32 (29.6) | 72 (66.7) | 95 (88.0) | 106 (98.1) | 102 (94.4) | 33 (30.6) | 107 (99.1) | 50 (46.3) | 16 (14.8) |
| Dire Dawa (n=103) | 40 (38.8) | 28 (26.9) | 99 (96.1) | 68 (66.0) | 103 (100.0) | 54 (52.4) | 99 (96.1) | 84 (81.6) | 43 (41.7) |
| Harar (n=105) | 70 (66.7) | 57 (54.3) | 104 (99.0) | 105 (100.0) | 105 (100.0) | 57 (54.3) | 105 (100.0) | 104 (99.0) | 33 (31.4) |
| Assosa (n=103) | 69 (67.0) | 33 (32.0) | 103 (100.0) | 90 (87.4) | 102 (99.0) | 30 (22.1) | 102 (99.0) | 86 (83.5) | 31 (30.4) |
| Gambella (n=101) | 65 (63.7) | 16 (15.8) | 94 (93.1) | 89 (88.1) | 101 (100.0) | 48 (47.5) | 99 (98.0) | 84 (83.2) | 61 (60.4) |
| P-value | <0.001 | <0.001 | <0.001 | <0.001 | <0.001 | <0.001 | 0.260 | <0.001 | <0.001 |
| **Hospitality venue (n=1368)** |  | | | | | | | | |
| Hotel (n=264) | 181 (68.6) | 124 (47.0) | 254 (96.2) | 241 (91.3) | 253 (95.8) | 179 (67.8) | 262 (99.2) | 227 (86.0) | 147 (55.7) |
| Bar and restaurant (n=237) | 173 (73.0) | 96 (40.5) | 229 (96.6) | 216 (91.1) | 233 (98.3) | 157 (66.2) | 234 (98.7) | 219 (92.4) | 112 (47.2) |
| Café and restaurant (n=204) | 166 (81.4) | 87 (42.6) | 198 (97.1) | 201 (98.5) | 204 (100.0) | 155 (76.0) | 204 (100.0) | 176 (86.3) | 137 (67.2) |
| Restaurant (n=201) | 148 (73.6) | 52 (25.9) | 190 (94.5) | 195 (97.0) | 198 (98.5) | 128 (63.7) | 201 (100.0) | 168 (83.6) | 113 (56.2) |
| Grocery (n=167) | 108 (64.7) | 36 (21.6) | 162 (97.0) | 142 (85.0) | 166 (99.4) | 99 (59.3) | 166 (99.4) | 118 (95.9) | 70 (41.9) |
| Butcher house and restaurant (n=123) | 94 (76.4) | 36 (29.3) | 120 (97.6) | 121 (98.4) | 123 (100.0) | 68 (55.3) | 123 (100.0) | 143 (85.6) | 63 (51.2) |
| Bar (n=102) | 35 (34.7) | 30 (29.7) | 85 (83.3) | 62 (61.4) | 99 (97.1) | 55 (53.9) | 94 (92.2) | 64 (62.7) | 29 (28.7) |
| Nightclub/lounge (n=70) | 22 (31.4) | 20 (28.6) | 59 (84.3) | 42 (60.0) | 67 (95.7) | 34 (48.6) | 56 (80.0) | 35 (50.0) | 16 (22.9) |
| P-value | <0.001 | <0.001 | <0.001 | <0.001 | 0.009 | <0.001 | <0.001 | <0.001 | <0.001 |
| **Overall compliance, n (%)** | **927 (67.8)** | **887 (64.8)** | **1297 (94.8)** | **1220 (89.2)** | **1343 (98.2)** | **875 (64.0)** | **1340 (98.0)** | **1150 (84.1)** | **687 (50.2)** |

^a^DSA - Designated Smoking Area

Only 12.5% of the indoor areas were fully compliant, and full compliance was the highest in Addis Ababa (21.8%) and cafés and restaurant (23.5%), while poor compliance was the highest at Semera-Logia (49.1%) and nightclubs/lounges (40%) (Table 3).

**Table 3. Indoor compliance level with smoke-free laws by cities and HVs**

| **City** | **Indoor compliance level, n (%)^a^** | | | | **Total venues** |
| --- | --- | --- | --- | --- | --- |
|  | **Fully compliant** | **Good** | **Moderate** | **Poor** |  |
| Addis Ababa | 62 (21.8) | 149 (52.5) | 55 (19.4) | 18 (6.3) | 284 |
| Adama | 27 (17.3) | 93 (59.6) | 25 (16.0) | 11 (7.1) | 156 |
| Hawassa | 28 (18.2) | 76 (49.4) | 21 (13.6) | 29 (18.8) | 154 |
| Bahir Dar | 6 (6.4) | 89 (61.0) | 39 (26.7) | 12 (8.2) | 146 |
| Jigjiga | 8 (7.4) | 89 (82.4) | 11 (10.2) | 0 | 108 |
| Semera-Logia | 4 (37) | 28 (25.9) | 53 (49.1) | 23 (21.3) | 108 |
| Dire Dawa | 8 (7.8) | 39 (37.9) | 30 (29.1) | 26 (25.2) | 103 |
| Harar | 13 (12.4) | 56 (53.3) | 36 (34.3) | 0 | 105 |
| Assosa | 10 (9.7) | 38 (36.9) | 45 (43.7) | 10 (9.7) | 103 |
| Gambella | 5 (5.0) | 49 (48.5) | 39 (38.6) | 8 (7.9) | 101 |
| **Hospitality venue** |  | | | | |
| Hotel | 44 (16.7) | 137 (51.9) | 65 (24.6) | 18 (6.8) | 264 |
| Bar and restaurant | 36 (15.2) | 133 (56.1) | 53 (22.4) | 15 (6.3) | 237 |
| Café and restaurant | 48 (23.5) | 113 (55.4) | 33 (16.2) | 10 (4.9) | 204 |
| Restaurant | 17 (8.5) | 122 (60.7) | 46 (22.9) | 16 (8.0) | 201 |
| Grocery | 9 (5.4) | 85 (50.9) | 55 (32.9) | 18 (10.8) | 167 |
| Butcher house and restaurant | 12 (9.8) | 69 (56.1) | 40 (32.5) | 2 (1.6) | 123 |
| Bar | 4 (3.9) | 27 (26.5) | 41 (40.2) | 30 (29.4) | 102 |
| Nightclub/lounge | 1 (1.4) | 20 (28.6) | 21 (30.0) | 28 (40.0) | 70 |
| **Total, n (%)** | **171 (12.5)** | **706 (51.6)** | **354 (25.9)** | **137 (10.0)** | **1368** |

^a^Fully compliant: compliance with nine indicators; Good compliance: compliance with 7-8 indicators; Moderate compliance: compliance with

5-6 indicators; Poor compliance: compliance with ≤4 indicators.

### Outdoor active smoking and enabling factors

Active smoking was observed in the outdoor space of 46.5% (95% CI:41.0-52.0) of the venues; and this was true for 65.7% of HVs in Semera-Logia and 51.6% of HVs in Adama, compared to 37.9% of HVs in Addis Ababa and 13.8% of HVs in Jigjiga. Active smoking in outdoor spaces was observed in 71.4% of the bars, 57.1% of the nightclubs/lounges, and 56.9% the restaurants. ‘No smoking’ signage was observed in the outdoor spaces of 33.1% (95% CI:28.0-38.0) of the HVs, with the highest proportions found in Semera-Logia (59.3%) and Addis Ababa (44.8%). Bars, groceries, nightclubs/lounges, and butcher shops and restaurants had the lowest rates of outdoor ‘no smoking’ signage. The presence of ashtrays (1.2%) and DSAs (3.6%) was very low in outdoor spaces.

### Compliance and adherence with smoke-free laws in outdoor spaces

Table 4 shows high outdoor adherence for the absence of ashtrays (98.8%) and DSAs (96.4%). However, lower compliance rates were observed for no active smoking (53.5%), ‘no smoking’ signage (33.1%), and absence of cigarette butts (38.6%). City comparisons show significant variations: Bahir Dar reported 100% compliance for no active smoking and ashtrays, but only 8.3% had ‘no smoking’ signage. Jigjiga demonstrated strong compliance (86.2%) for no active smoking, but there was no ‘no smoking’ signage observed. Semera-Logia had the lowest rates for no active smoking (34.3%) although it had the highest rate for signage (59.3%). Hotels and cafés also showed relatively high compliance and adherence, unlike bars and nightclubs/lounges, which had very low no active smoking and ‘no smoking’ signage.

**Table 4. Compliance and adherence with smoke-free laws indicators in outdoor spaces by cities and HVs**

| **City (n=329)** | **Outdoor smoke-free indicator, n (%)** | | | | |
| --- | --- | --- | --- | --- | --- |
|  | **No active smoking** | **‘No smoking’ signage** | **No cigarette butts** | **No ashtray** | **No DSA^a^** |
| Addis Ababa (n=29) | 18 (62.3) | 13 (44.8) | 17 (58.6) | 27 (96.4) | 29 (100.0) |
| Adama (n=31) | 15 (48.4) | 7 (22.6) | 22 (71.0) | 31 (100.0) | 26 (83.9) |
| Hawassa (n=37) | 21 (56.8) | 9 (24.3) | 18 (48.6) | 36 (97.3) | 34 (91.9) |
| Bahir Dar (n=12) | 12 (100.0) | 1 (8.3) | 10 (83.3) | 12 (100.0) | 12 (100.0) |
| Jigjiga (n=29) | 25 (86.2) | 0 | 10 (34.5) | 29 (100.0) | 29 (100.0) |
| Semera-Logia (n=108) | 37 (34.3) | 64 (59.3) | 21 (19.4) | 107 (99.1) | 106 (98.1) |
| Dire Dawa (n=32) | 18 (56.3) | 3 (9.4) | 17 (53.1) | 32 (100.0) | 32 (100.0) |
| Harar (n=13) | 7 (53.8) | 1 (7.7) | 1 (7.7) | 13 (100.0) | 13 (100.0) |
| Assosa (n=21) | 12 (57.8) | 9 (42.9) | 4 (19.0) | 21 (100.0) | 21 (100.0) |
| Gambella (n=17) | 11 (64.7) | 2 (11.8) | 7 (41.2) | 17 (100.0) | 15 (88.2) |
| *P-value* | <0.001 | <0.001 | <0.001 | 0.844 | 0.003 |
| **Hospitality venue (n=329)** |  | | | | |
| Hotel (n=102) | 58 (56.9) | 38 (37.3) | 38 (37.3) | 100 (98.0) | 95 (93.1) |
| Bar and restaurant (n=51) | 29 (58.0) | 12 (23.5) | 27 (52.9) | 51 (100.0) | 48 (94.1) |
| Café and restaurant (n=63) | 34 (54.0) | 33 (52.4) | 19 (30.2) | 63 (100.0) | 62 (98.4) |
| Restaurant (n=58) | 25 (43.1) | 21 (36.2) | 22 (37.9) | 57 (98.3) | 57 (98.3) |
| Grocery (n=20) | 12 (60.0) | 1 (5.0) | 8 (40.0) | 20 (100.0) | 20 (100.0) |
| Butcher house and restaurant (n=21) | 13 (61.9) | 3 (10.0) | 7 (33.3) | 21 (100.0) | 21 (100.0) |
| Bar (n=7) | 2 (28.6) | 0 | 3 (42.9) | 7 (100.0) | 7 (100.0) |
| Nightclub/lounge (n=7) | 3 (42.9) | 1 (14.3) | 3 (42.9) | 7 (100.0) | 7 (100.0) |
| *P-value* | 0.514 | <0.001 | <0.001 | 0.942 | 0.411 |
| **Overall compliance, n (%)** | **176 (53.5)** | **109 (33.1)** | **127 (38.6)** | **325 (98.8)** | **317 (96.4)** |

^a^DSA - Designated Smoking Area

About 10.3% of outdoor spaces in the HVs were fully compliant, whereas about a quarter (26.4%) were found to be in poor compliance with smoke-free laws (Table 5).

**Table 5. Outdoor compliance level with smoke-free laws by cities and HVs**

| **City** | **Outdoor compliance level, n (%)^a^** | | | | **Total venues** |
| --- | --- | --- | --- | --- | --- |
|  | **Fully compliant** | **Good** | **Moderate** | **Poor** |  |
| Addis Ababa | 8 (27.6) | 8 (27.6) | 6 (20.7) | 7 (24.1) | 29 |
| Adama | 2 (6.5) | 9 (29.0) | 15 (48.4) | 5 (16.1) | 31 |
| Hawassa | 5 (13.5) | 10 (27.0) | 10 (27.0) | 12 (32.4) | 37 |
| Bahir Dar | 1 (8.3) | 9 (75.0) | 2 (16.7) | 0 | 12 |
| Jigjiga | 0 | 10 (34.5) | 15 (51.7) | 4 (13.8) | 29 |
| Semera-Logia | 13 (12.0) | 16 (14.8) | 50 (46.3) | 29 (26.9) | 108 |
| Dire Dawa | 2 (6.3) | 14 (43.8) | 4 (12.5) | 12 (37.5) | 32 |
| Harar | 0 | 1 (7.7) | 7 (53.8) | 5 (38.5) | 13 |
| Assosa | 3 (14.3) | 5 (23.8) | 6 (28.6) | 7 (33.3) | 21 |
| Gambella | 0 | 7 (41.2) | 4 (23.5) | 6 (35.3) | 17 |
| **Hospitality venue** |  | | | | |
| Hotel | 9 (8.8) | 31 (30.4) | 36 (35.3) | 26 (25.5) | 102 |
| Bar and restaurant | 7 (13.7) | 14 (27.5) | 16 (31.4) | 14 (27.5) | 51 |
| Café and restaurant | 9 (14.3) | 15 (23.8) | 29 (46.0) | 10 (15.9) | 63 |
| Restaurant | 7 (12.1) | 13 (22.4) | 20 (34.5) | 18 (31.0) | 58 |
| Grocery | 0 | 8 (40.0) | 5 (25.0) | 7 (35.0) | 20 |
| Butcher house and restaurant | 1 (4.8) | 5 (23.8) | 10 (47.6) | 5 (23.8) | 21 |
| Bar | 0 | 1 (14.3) | 3 (42.9) | 3 (42.9) | 7 |
| Nightclub/lounge | 1 (14.3) | 2 (28.6) | 0 | 4 (57.1) | 7 |
| **Total, n (%)** | **34 (10.3)** | **89 (27.1)** | **119 (36.2)** | **87 (26.4)** | **329** |

^a^Fully compliant: compliance with five indicators; Moderate compliance: compliance with 3-4 indicators;

Poor compliance: compliance with <3 indicators.

**Factors associated with indoor active smoking and presence of cigarette butts**

Table 6 presents results from a bivariate and multivariable Poisson regression model examining predictors of ‘active smoking’ and presence of ‘cigarette butts’ in indoor HVs. The prevalence of active smoking and cigarette butts were significantly higher in Semera-Logia and Dire Dawa, both before and after adjustment. After adjustment, venues in Semera-Logia were over two times more likely to have ‘active smoking’ (aPR: 2.71; 95% CI: 2.00–3.66), while cigarette butts prevalence was about three times higher (aPR: 2.96; 95% CI: 2.23–3.93) than Addis Ababa. Groceries, bars, and nightclubs/lounges had significantly higher prevalence of active smoking and cigarette butts than cafés/restaurants, both before and after adjustment. ‘No smoking’ signs were associated with reduced prevalence of ‘active smoking’ (aPR: 0.77; 95% CI: 0.67–0.89). The presence of lighters was strongly associated with higher prevalence of both active smoking (aPR: 1.69; 95% CI: 1.41–2.02 and cigarette butts (aPR: 1.90; 95% CI: 1.58–2.27), before and after adjustment. The presence of tobacco products for sale (aPR: 1.58; 95% CI: 1.34–1.86) and someone smoking within 10-meter range of air intake (aPR: 2.67; 95% CI: 2.13–3.32) strongly associated with higher prevalence of active smoking, before and after adjustment.

**Table 6. Bivariate and multivariable Poisson regression predictors of presence of ‘active smoking’ and ‘cigarette butts’ in indoor places**

| **City** | **Active smoking** | | **Cigarette butts** | |
| --- | --- | --- | --- | --- |
|  | **cPR (95% CI)^a^** | **aPR^b^ (95% CI)** | **cPR (95% CI)** | **aPR (95% CI)** |
| Addis Ababa | 1^c^ | 1 | 1 | 1 |
| Adama | 0.95 (0.68-1.32) | 1.10 (0.81-1.49) | 0.65 (0.43-0.97) | 0.73 (0.50-1.07) |
| Hawassa | 1.03 (0.75-1.43) | 0.75 (0.58-0.97) | 1.09 (0.79-1.50) | 0.92 (0.70-1.22) |
| Bahir Dar | 0.86 (0.60-1.22) | 0.83 (0.59-1.16) | 0.93 (0.66-1.32) | 1.10 (0.76-1.59) |
| Jigjiga | 0.28 (0.14-0.56) | 0.55 (0.28-1.08) | 0.65 (0.41-1.03) | 1.08 (0.68-1.70) |
| Semera-Logia | 2.67 (2.12-3.35) | 2.71 (2.00-3.66) | 2.70 (2.14-3.41) | 2.96 (2.23-3.93) |
| Dire Dawa | 2.32 (1.81-2.97) | 1.47 (1.15-1.88) | 1.85 (1.39-2.46) | 1.39 (1.07-1.80) |
| Harar | 1.26 (0.91-1.76) | 1.53 (1.10-2.15) | 1.78 (1.33-2.37) | 1.76 (1.29-2.40) |
| Assosa | 1.25 (0.89-1.75) | 0.98 (0.72-1.34) | 2.76 (2.18-3.48) | 2.57 (2.02-3.27) |
| Gambella | 1.35 (0.97-1.87) | 1.51 (1.11-2.07) | 2.04 (1.56-2.68) | 2.27 (1.73-2.97) |
| **Hospitality venue** |  | | | |
| Café and restaurant | 1 | 1 | 1 | 1 |
| Hotel | 1.69 (1.21-2.37) | 1.96 (1.51-2.54) | 1.34 (0.99-1.81) | 1.20 (0.92-1.56) |
| Bar and restaurant | 1.45 (1.02-2.07) | 1.86 (1.36-2.56) | 1.41 (1.04-1.90) | 1.61 (1.22-2.13) |
| Restaurant | 1.42 (0.98-2.05) | 1.36 (1.04-1.77) | 1.51 (1.11-2.05) | 1.39 (1.08-1.78) |
| Grocery | 1.90 (1.33-2.70) | 1.99 (1.43-2.76) | 1.70 (1.25-2.30) | 1.53 (1.14-2.06) |
| Butcher house and restaurant | 1.27 (0.83-1.94) | 1.55 (1.02-2.36) | 1.86 (1.36-2.55) | 1.86 (1.36-2.53) |
| Bar | 3.53 (2.56-4.85) | 2.82 (2.06-3.87) | 1.92 (1.39-2.65) | 1.65 (1.21-2.25) |
| Nightclub/lounge | 3.68 (2.65-5.11) | 2.44 (1.76-3.39) | 2.14 (1.53-2.99) | 1.38 (0.97-1.97) |
| **Enabling factors** |  |  |  |  |
| ‘No smoking’ sign (yes/no) | 0.90 (0.77-1.07) | 0.77 (0.67-0.89) | 0.96 (0.83-1.12) | 0.91 (0.79-1.05) |
| Presence of ashtray (yes/no) | 2.50 (2.13-2.93) | 1.11 (0.92-1.34) | 1.97 (1.65-2.35) | 1.20 (0.97-1.49) |
| Presence of lighter (yes/no) | 3.33 (2.97-3.74) | 1.69 (1.41-2.02) | 2.37 (2.09-2.69) | 1.90 (1.58-2.27) |
| Presence of shisha equipment (yes/no) | 2.75 (2.32-3.27) | 1.01 (0.77-1.34) | 2.02 (1.58-2.59) | 1.27 (0.92-1.76) |
| Presence of DSA (yes/no) | 2.29 (1.77-2.96) | 1.06 (0.77-1.47) | 1.80 (1.33-2.44) | 1.44 (0.97-2.13) |
| Tobacco product sale (yes/no) | 3.25 (2.86-3.68) | 1.58 (1.34-1.86) | 1.97 (1.72-2.23) | 1.03 (0.87-1.21) |
| Smoking within 10m (yes/no) | 4.29 (3.47-5.30) | 2.67 (2.13-3.32) | 2.79 (2.35-3.30) | 1.94 (1.63-2.32) |

^a^Crude Prevalence Ratio and 95% Confidence Interval; ^b^Adjusted Prevalence Ratio; ^c^Reference

**Discussion**

The findings of this study highlight significant discrepancies in compliance with smoke-free laws across various indicators. Compliance was lower for indoor active smoking (67.8%), while adherences were notably low for cigarette butts (64%) and the ‘no smoking’ signage (47%). In contrast, adherence was highest for the absence of DSAs (98.2%) and shisha equipment (98%), along with ashtrays (94.8%) and lighters (89.2%). Outdoor compliance regarding active smoking was concerning at only 53.5%, with adherence to signage even lower (33.1%). Only 12.5% of venues were fully compliant in indoor areas and 10.3% in outdoor spaces.

Active smoking was observed in 32.2% of indoor areas and 49.5% of outdoor spaces across various HVs and cities, which is higher than a previous Ethiopian study from 2021 that reported active smoking in only 7% of bars, restaurants, and cafés, and 10% in hotels [10]. This discrepancy may arise from differences in observation timing and variations in venue types. A study in Ghana found active smoking in 34.5% of restaurants and 63.6% of bars [11], while in Uganda, it was 18% in HVs [8]. These variations underscore the inconsistent implementation and enforcement of smoke-free laws across HV types and regions within and between countries. The higher prevalence of outdoor smoking in our study suggests that smokers may be shifting to outdoor areas in response to indoor smoking bans. Furthermore, HV managers may encourage outdoor smoking if indoor smoking makes customers uncomfortable. A review of compliance facilitators and barriers highlights that smokers with greater knowledge of smoking harms and supportive attitudes toward bans are more likely to adhere to them, while heavier nicotine dependence and negative attitudes can impede compliance [25].

Although the presence of ‘no smoking’ signage was low in our study (35% in indoors and 33% in outdoor spaces), it was still higher than a previous study in Ethiopia, which reported only 17% of bars and restaurants, and 29% of hotels posted ‘no smoking’ signage [10]. In Ghana, the presence of such signage was 49.5% [11], with similar findings in other studies [26, 27]. Interestingly, our study found exceptionally high adherence rates regarding the absence of DSAs and shisha equipment, indicating that smoke-free legislation is effective in specific contexts where enforcement and public health campaigns are prioritized. However, these findings must be viewed alongside lower compliance rates for key indicators like active smoking and cigarette butts, indicating that while venues may be successful in removing smoking aids, challenges remain in ensuring comprehensive adherence to all smoke-free laws [10, 28]. Thus, strategies to maintain high levels of compliance for all aspects of smoke-free legislation are essential to protect public health effectively.

Our study revealed regional differences in compliance with smoke-free laws at HVs in Ethiopia, with higher rates in cities like Addis Ababa, Jigjiga, Adama, and Harar, and lower rates in Dire Dawa and Semera-Logia. These differences may be attributed to variations in the local tobacco control environment and the implementation of smoke-free laws. Compliance rates also varied across venue types, with cafés and restaurants demonstrating the highest adherence, while bars and nightclubs/lounges consistently displayed the lowest. This aligns with findings from other studies, indicating that HVs like bars and nightclubs often show low compliance [29]. In Uganda, compliance was particularly low in bars and restaurants, highlighting ongoing challenges in these venues [30]. Thus, targeted interventions in high-risk venues and cities where smoking is normalized are essential. Implementing stronger penalties, consistent monitoring, and removing enabling factors such as tobacco product sales and lighters could enhance compliance.

The presence of 'no smoking' signage was significantly associated with reduced active smoking rates and the prevalence of cigarette butts. In contrast, the presence of lighters, tobacco product sales, and outdoor smoking within 10-meter range of indoor air intakes were associated with increased smoking rates and cigarette butt prevalence. This aligns with research from Turkey, where indoor cigarette sales and ashtrays were strongly associated with higher indoor smoking rates and cigarette butt availability [31]. In Greece, ashtrays were strongly associated with increased particulate matter (PM_2.5_) levels, indicating their role in encouraging smoking behaviors [32]. However, our research found that ashtrays did not predict non-compliance with smoke-free laws. Notably, while the Turkish study indicated that 'no smoking' signage did not associate with reduced smoking prevalence, our study showed a significant association between such signage and decreased active smoking rates and cigarette butt prevalence. This finding is supported by research other studies [10, 27], where a significant association was found between the presence of 'no smoking' signage and reduced active smoking. These results highlight the necessity for context-specific enforcement strategies and enhanced visual enforcement, such as prominent ‘no smoking’ signage.

Smoking within a 10-meter radius of indoor air intakes poses a significant risk for SHS exposure in HVs, as outdoor tobacco smoke concentrations can easily reach indoors [33]. Our findings show that smoking within this proximity is associated with increased indoor smoking activity and a higher prevalence of cigarette butts, undermining smoke-free policies designed to safeguard indoor air quality. Studies confirm that outdoor tobacco smoke levels remain hazardous even at distances up to nine meters from the source and can infiltrate indoor environments via air intakes [33, 34]. Implementing buffer zones around entrances and ventilation intakes could help mitigate SHS infiltration, ensuring better protection for patrons and staff in smoke-free venues.

In our study, full compliance with smoke-free laws was observed in only 12.5% of indoor venues and 10.3% of outdoor spaces, highlighting the significant challenges in enforcing smoke-free regulations, particularly in bars and nightclubs/lounges, and in cities such as Semera-Logia, where adherence was notably low. These findings are consistent with a recent study across four regions in Ethiopia, which reported 12% full compliance in public places [10]. However, the public places assessed in that study, such as government buildings, educational facilities, and transport hubs, differ from the HVs included in our research. Similar variability in compliance has been noted in other countries; for example, Ghana reported a 70% compliance rate [35], while Bangladesh showed 58% compliance indoors and only 27% outdoors [28]. Meanwhile, studies from Nepal [27] and India [26] reported compliance rates of 26% and 80% in HVs, respectively. These differences emphasize the variability of compliance across settings and suggest that targeted interventions are essential, particularly in environments where social activities and tobacco use are prevalent.

Strict enforcement significantly boosts compliance with smoking bans, as evidenced by a very high compliance rate of 98% in Punjab, India [26]. High compliance levels are often achieved through robust enforcement infrastructure, regular inspections, and penalties [25, 36]. Countries that actively involve local authorities, strong political support for smoke-free legislation, and implement prominent ‘no smoking’ signage see substantial compliance improvements [25, 36]. Involving local jurisdictions in enforcement, particularly through training for inspections, is crucial for maintaining high compliance rates [36]. This localized involvement enables authorities to address specific community dynamics, enhancing enforcement effectiveness. Efficient smoking ban implementation, including clear rewards and punishments, reduces non-compliance [25]. Conversely, inadequate surveillance leads to higher smoking rates, underscoring the need for consistent monitoring. Furthermore, enabling factors like ashtrays in public spaces contribute to increased smoking rates even after smoke-free laws are enacted [25].

The findings of this study have significant policy implications for enhancing tobacco control efforts in Ethiopia, particularly in cities and HVs with lower compliance. The observed variability in compliance across different locations underscores the necessity of tailoring strategies to fit local contexts and dynamics. Strengthening the implementation of Proclamation No. 1112/2019 [18] and Directive No. 771/2021 [22] is crucial for establishing a robust enforcement framework. Increasing the visibility and enforcement of ‘no smoking’ signage can serve as an effective policy measure, as evidenced by successful practices in other countries where clear signage and local authority involvement significantly boosted compliance rates [25, 36]. Future research should investigate the underlying factors contributing to the low compliance rates observed in HVs, such as bars and nightclubs/lounges, as well as in Semera-Logia city. This exploration will help identify targeted strategies for enhancing enforcement and adherence to smoke-free laws.

**Strengths and limitations**

This study is the first large-scale investigation of HVs across 10 major cities in Ethiopia, ensuring diverse context representations. The use of covert observations enhanced data authenticity, providing accurate compliance assessments. The inclusion of multiple indoor and outdoor smoke-free laws indicators offers a detailed understanding of compliance and adherence levels. However, the study has limitations. Incomplete lists of registered HVs may have led to sampling bias, and the cross-sectional design limits causal conclusions. Despite these limitations, the study provides valuable data and highlights areas needing targeted efforts to improve compliance with smoke-free laws.

**Conclusions**

Compliance with smoke-free laws in HVs remains low, particularly in bars, nightclubs/lounges, and Semera-Logia, with high rates of active smoking both indoors and outdoors. Stronger enforcement and targeted interventions are needed to address the low compliance with smoke-free laws. Collaboration among tobacco control stakeholders and the active engagement of local authorities is essential to educate the public and HV owners about the risks of SHS and foster a healthier community. Enhanced monitoring of compliance, improved enforcement of 'no smoking’ signage, and stricter penalties for violations are crucial. Expanding efforts to include informal venues could also provide a more comprehensive understanding of compliance.

**Acknowledgements**

We would like to thank the study team members from Addis Ababa University School of Public Health, Development Gateway: An IREX Venture, Tobacco Control Data Initiative Ethiopia, Ethiopian Food and Drug Authority, and Regional Tobacco Control Regulatory Offices for their valuable support in this study. We are also grateful to all of our data collectors, supervisors, and coordinators who actively participated in this study.

**Author contributions**

**Conceptualization**: Selam Abraham Kassa, Terefe Gelibo Argefa, Noreen Dadirai Mdeg, Rachel Kitonyo-Devotsu, Winnie Awuor, Wakgari Deressa, Selamawit Hirpa

**Data curation:** Wakgari Deressa, Selamawit Hirpa, Terefe Gelibo Argefa

**Formal analysis:** Wakgari Deressa

**Funding acquisition:** Selam Abraham Kassa, Terefe Gelibo Argefa, Noreen Dadirai Mdeg, Rachel Kitonyo-Devotsu, Winnie Awuor

**Investigation:** Wakgari Deressa, Selamawit Hirpa, Yifokire Tefera

**Methodology:** Wakgari Deressa, Selamawit Hirpa, Terefe Gelibo Argefa, Selam Abraham Kassa, Noreen Dadirai Mdeg, Rachel Kitonyo-Devotsu, Winnie Awuor, Yifokire Tefera, Baharu Zewdie

**Project administration:** Wakgari Deressa, Selamawit Hirpa

**Supervision:** Wakgari Deressa, Selamawit Hirpa, Yifokire Tefera, Baharu Zewdie

**Writing – original draft:** Wakgari Deressa

**Writing – review and editing:** Wakgari Deressa, Selamawit Hirpa, Noreen Dadirai Mdeg, Terefe Gelibo Argefa, Yifokire Tefera, Rachel Kitonyo-Devotsu, Winnie Awuor, Selam Abraham Kassa, Baharu Zewdie.

**Funding**

This study was supported with funding from the Bill and Melinda Gates Foundation (grant number INV-009670). The findings and conclusions contained in the study are those of the authors and do not necessarily reflect the positions and policies of the donor. The funder had no role in study design, data collection and analysis, decision to publish, or preparation of the manuscript.

**Data availability statement**

All relevant data are within the paper and it’s Supporting Information (S3_dataset).

## Consent for publication

Not applicable

**Competing interests**

The authors declare no competing interests.

**References**

1. Ethiopian Public Health Institute. Global Adult Tobacco Survey: Country Report 2016. 2016. Addis Ababa, Ethiopia.
2. Defar A, Getachew T, Teklie H, Bekele A, Gonfa G, Gelibo T, et al. Tobacco use and its predictors among Ethiopian adults: A further analysis of Ethiopian NCD STEPS survey-2015. Ethiopian J Health Dev. 2017;31(Special Issue):331-339.
3. Central Statistical Agency and ICF International. Ethiopia Demographic and Health Survey 2016. Addis Ababa, Ethiopia.
4. Mengesha SD, Teklu KT, Weldetinsae A, Serte MG, Kenea MA, Dinsa DA, et al*.* Tobacco use prevalence and its determinate factor in Ethiopia- finding of the 2016 Ethiopian GATS. BMC Public Health 2022; **22:**555. <https://doi.org/10.1186/s12889-022-12893-8>.
5. World Health Organization. Guidelines for implementation of Article 8 of the WHO Framework Convention on Tobacco Control. WHO, 2013. Geneva, Switzerland.
6. Garritsen HH, da Costa Senior YY, Rozema AD, Kunst AE, Kuipers MAG. Association Between Smoke-Free Legislation in Hospitality Venues and Smoking Behavior of Young People: A Systematic Review. Nicotine Tob Res. 2022;24(6):807-812. doi: 10.1093/ntr/ntab129.
7. Hawkins SS, Bach N, Baum CF. Impact of Tobacco Control Policies on Adolescent Smoking. J Adolesc Health. 2016;58(6):679-85. doi: 10.1016/j.jadohealth.2016.02.014.
8. Gravely S, Nyamurungi KN, Kabwama SN, Okello G, Robertson L, Heng K, et al. (2018). Knowledge, opinions and compliance related to the 100% smoke-free law in hospitality venues in Kampala, Uganda: cross-sectional results from the *KOMPLY* Project. BMJ Open, 2018;8(1), e017601. [doi.org/10.1136/bmjopen-2017-017601](https://doi.org/10.1136/bmjopen-2017-017601).
9. Singh A, Dobbie F, Kinnunen TI, Okello G, Semple S, Okyere PB, et al. Adherence to smoke-free policies in Ghana: Findings from a cross-sectional survey of hospitality venue owners and staff. Tob Prev Cessat. 2021;7:4. doi: 10.18332/tpc/131058.
10. Mengesha SD, Shimeles B, Zewdie B, Alermu A, Gerba H, Gartner CE. Smoke-free law compliance and predictive factors in Ethiopia: observational assessment of public places and workplaces. Tob Control. 2023:tc-2022-057750. doi: 10.1136/tc-2022-057750.
11. Singh A, Okello G, Semple S, Dobbie F, Kinnunen TI, Lartey KF, et al. Exposure to secondhand smoke in hospitality settings in Ghana: Evidence of changes since implementation of smoke-free legislation. Tob Induc Dis. 2020;18:44. doi: 10.18332/tid/120934.
12. Karimi KJ, Ayah R, Olewe T. Adherence to the Tobacco Control Act, 2007: presence of a workplace policy on tobacco use in bars and restaurants in Nairobi, Kenya. BMJ Open. 2016;6(9):e012526. doi: 10.1136/bmjopen-2016-012526.
13. Maskarinec MP, Jenkins RA, Counts RW, Dindal AB. Determination of exposure to environmental tobacco smoke in restaurant and tavern workers in one US city. J Expo Anal Environ Epidemiol. 2000;10(1):36-49. doi: 10.1038/sj.jea.7500069.
14. Jones MR, Wipfli H, Shahrir S, Avila-Tang E, Samet JM, Breysse PN, et el. Secondhand tobacco smoke: an occupational hazard for smoking and non-smoking bar and nightclub employees. Tob Control. 2013;22(5):308-14. doi: 10.1136/tobaccocontrol-2011-050203.
15. Siegel M, Skeer M. Exposure to secondhand smoke and excess lung cancer mortality risk among workers in the "5 B's": bars, bowling alleys, billiard halls, betting establishments, and bingo parlours. Tob Control. 2003;12(3):333-8. doi: 10.1136/tc.12.3.333.
16. Lewis SA, Antoniak M, Venn AJ, Davies L, Goodwin A, Salfield N, et al. Secondhand smoke, dietary fruit intake, road traffic exposures, and the prevalence of asthma: a cross-sectional study in young children. Am J Epidemiol. 2005;161(5):406-11. doi: 10.1093/aje/kwi059.
17. Tanaka K, Miyake Y, Arakawa M, Sasaki S, Ohya Y. Prevalence of asthma and wheeze in relation to passive smoking in Japanese children. Ann Epidemiol. 2007;17(12):1004-10. doi: 10.1016/j.annepidem.2007.07.108.
18. The Federal Democratic Republic of Ethiopia (FDRE), Federal Negarit Gazette Proclamation No.1112/2019. A Proclamation to Provide for Food and Medicine Administration. 28^th^ February 2019. Addis Ababa, Ethiopia.
19. Argefa TG, Kassa SA, Mdege ND. Second-Hand Tobacco Smoke Exposure and Smoke-Free Environments in Ethiopia: A Scoping Review and Narrative Synthesis. Int. J. Environ. Res. Public Health 2022, 19, 8404. https://doi.org/10.3390/ijerph19148404.
20. Tadesse, T. and B. Zawdie, Non-compliance and associated factors against smoke-free legislation among health care staff in governmental hospitals in Addis Ababa, Ethiopia: An observational cross-sectional study. BMC Public Health, 2019. 19(1):1-11.
21. International Union Against Tuberculosis and Lung Disease, Campaign for Tobacco-Free Kids, and Johns Hopkins Bloomberg School of Public Health. Assessing Compliance with Smoke-Free Laws: A”How-to” Guide for Conducting Compliance Studies. Second Edition. 2014; 1–36. https://theunion.org/technical-publications/assessing-compliance-with-smoke-free-laws.
22. Ethiopian Food Medicine and Healthcare Administration and Control Authority (FMHACA). Tobacco Control Directive No. 771/2021. 2021. Addis Ababa, Ethiopia.
23. Petticrew M, Semple S, Hilton S, Creely KS, Eadie D, Ritchie D, et al. Covert observation in practice: lessons from the evaluation of the prohibition of smoking in public places in Scotland. BMC Public Health. 2007;7:204. doi: 10.1186/1471-2458-7-204.
24. Barros AJ, Hirakata VN. Alternatives for logistic regression in cross-sectional studies: an empirical comparison of models that directly estimate the prevalence ratio. BMC Med Res Methodol. 2003;3:21. doi: 10.1186/1471-2288-3-21.
25. Zhou L, Niu L, Jiang H, Jiang C, Xiao S. Facilitators and Barriers of Smokers' Compliance with Smoking Bans in Public Places: A Systematic Review of Quantitative and Qualitative Literature. Int J Environ Res Public Health. 2016;13(12):1228. doi: 10.3390/ijerph13121228.
26. Goel S, Sharma D, Gupta R, Mahajan V. Compliance with smoke-free legislation and smoking behaviour: observational field study from Punjab, India. Tob Control. 2018;27(4):407-413. doi: 10.1136/tobaccocontrol-2016-053559.
27. Basnet LB, Budhathoki SS, Adhikari B, Thapa J, Neupane B, Moses T, et al. Compliance with the smoke-free public places legislation in Nepal: A cross-sectional study from Biratnagar Metropolitan City. PLoS One. 2022;17(3):e0264895. doi: 10.1371/journal.pone.0264895.
28. Chowdhury SR, Sunna TC, Das DC, Chowdhury MR, Mahmud HMM, Hossain A. Compliance with smoke-free legislation in public places: An observational study in a northeast city of Bangladesh. PLoS One. 2023;18(4):e0283650. doi: 10.1371/journal.pone.0283650.
29. Barnoya J, Navas-Acien A. Protecting the world from secondhand tobacco smoke exposure: where do we stand and where do we go from here? Nicotine Tob Res. 2013;15(4):789-804. doi: 10.1093/ntr/nts200.
30. Kabwama SN, Kadobera D, Ndyanabangi S, Nyamurungi KN, Gravely S, Robertson L, et al. Practices related to tobacco sale, promotion and protection from tobacco smoke exposure in restaurants and bars in Kampala before implementation of the Uganda tobacco control Act 2015. Tob Induc Dis. 2017 May 2;15:24. doi: 10.1186/s12971-017-0129-8.
31. Navas-Acien A, Çarkoğlu A, Ergör G, Hayran M, Ergüder T, Kaplan B, et al. Compliance with smoke-free legislation within public buildings: a cross-sectional study in Turkey. Bull World Health Organ. 2016;94(2):92-102. doi: 10.2471/BLT.15.158238.
32. Vardavas CI, Agaku I, Patelarou E, Anagnostopoulos N, Nakou C, Dramba V, et al. Hellenic Air Monitoring Study Investigators. Ashtrays and signage as determinants of a smoke-free legislation's success. PLoS One. 2013;8(9):e72945. doi: 10.1371/journal.pone.0072945.
33. Klepeis NE, Ott WR, Switzer P. Real-time measurement of outdoor tobacco smoke particles. J Air Waste Manag Assoc. 2007;57(5):522-34. doi: 10.3155/1047-3289.57.5.522.
34. Hwang J, Lee K. Determination of outdoor tobacco smoke exposure by distance from a smoking source. Nicotine Tob Res. 2014;16(4):478-84. doi: 10.1093/ntr/ntt178.
35. Singh A, Dobbie F, Kinnunen TI, Okello G, Semple S, Okyere PB, et al. Adherence to smoke-free policies in Ghana: Findings from a cross-sectional survey of hospitality venue owners and staff. Tob Prev Cessat. 2021;7:4. doi: 10.18332/tpc/131058.
36. Peruga A, Hayes LS, Aguilera X, Prasad V, Bettcher DW. Correlates of compliance with national comprehensive smoke-free laws. Tob Control. 2017:tobaccocontrol-2017-053920. doi: 10.1136/tobaccocontrol-2017-053920.

**Supporting information**

**S1 Fig.** **Multistage schematic sampling technique**

**S1 File. Smoke-free study tool**

**S2 File. Operational definitions**

**S3 File. Dataset**
